# Assessment of the Red Cell Proteome of Young Patients with Unexplained Hemolytic Anemia by Two-Dimensional Differential In-Gel Electrophoresis (DIGE)

**DOI:** 10.1371/journal.pone.0034237

**Published:** 2012-04-03

**Authors:** Katharina von Löhneysen, Thomas M. Scott, Katrin Soldau, Xiuling Xu, Jeffrey S. Friedman

**Affiliations:** Department of Molecular and Experimental Medicine, The Scripps Research Institute, La Jolla, California, United States of America; Shantou University Medical College, China

## Abstract

Erythrocyte cytosolic protein expression profiles of children with unexplained hemolytic anemia were compared with profiles of close relatives and controls by two-dimensional differential in-gel electrophoresis (2D-DIGE). The severity of anemia in the patients varied from compensated (i.e., no medical intervention required) to chronic transfusion dependence. Common characteristics of all patients included chronic elevation of reticulocyte count and a negative workup for anemia focusing on hemoglobinopathies, morphologic abnormalities that would suggest a membrane defect, immune-mediated red cell destruction, and evaluation of the most common red cell enzyme defects, glucose-6-phosphate dehydrogenase and pyruvate kinase deficiency. Based upon this initial workup and presentation during infancy or early childhood, four patients classified as hereditary nonspherocytic hemolytic anemia (HNSHA) of unknown etiology were selected for proteomic analysis. DIGE analysis of red cell cytosolic proteins clearly discriminated each anemic patient from both familial and unrelated controls, revealing both patient-specific and shared patterns of differential protein expression. Changes in expression pattern shared among the four patients were identified in several protein classes including chaperons, cytoskeletal and proteasome proteins. Elevated expression in patient samples of some proteins correlated with high reticulocyte count, likely identifying a subset of proteins that are normally lost during erythroid maturation, including proteins involved in mitochondrial metabolism and protein synthesis. Proteins identified with patient-specific decreased expression included components of the glutathione synthetic pathway, antioxidant pathways, and proteins involved in signal transduction and nucleotide metabolism. Among the more than 200 proteins identified in this study are 21 proteins not previously described as part of the erythrocyte proteome. These results demonstrate the feasibility of applying a global proteomic approach to aid characterization of red cells from patients with hereditary anemia of unknown cause, including the identification of differentially expressed proteins as potential candidates with a role in disease pathogenesis.

## Introduction

Red blood cells (RBC), the most abundant cell type in the human body, are highly specialized structurally and functionally to supply oxygen to tissues via the circulatory system. Erythrocyte development begins with marrow progenitors under the influence of lineage specific hematopoietic growth factors, with erythropoietin being the critical growth factor governing RBC production. Marrow RBC development progresses until immature RBC extrude their nuclei and exit the bone marrow as newly formed reticulocytes [1]. Reticulocytes circulate for a few days during which organelles including mitochondria, Golgi apparatus and the endoplasmic reticulum are lost [2], allowing mature RBC maximal flexibility to squeeze though narrow capillaries and providing room to pack the cell with hemoglobin, ultimately making up 90% of the dry weight of the cell [3]. Because of their extreme specialization, mature RBC have little capacity to repair and no ability to replace damaged proteins. RBC are routinely exposed to high oxygen concentrations and are vulnerable to the accumulation of damage caused by oxidative stress - with much of their metabolic activity devoted to reducing oxidative damage. Therefore a highly reducing milieu and a stable redox balance are paramount for proper function and cell survival. Major components of RBC defense against reactive oxygen species (ROS) include reduced glutathione (GSH) and enzymatic antioxidants such as catalase, peroxiredoxins and superoxide dismutase. Glycolysis and the pentose phosphate pathway are the only sources for NADH and NADPH (respectively) needed to protect RBC from oxidative damage. NADH is required for reduction of methemoglobin; NADPH is utilized primarily for the reduction of oxidized glutathione (GSSG ⇒ 2GSH). Disruptions in redox balance and increased oxidative damage are characteristic of many RBC pathologies including hemoglobinopathies such as sickle cell anemia [4] and thalassemia [5], as well as enzyme defects such as glucose-6-phosphate dehydrogenase (G6PD) deficiency [6] and pyruvate kinase (PK) deficiency [7].

Hereditary non-spherocytic hemolytic anemias (HNSHA) are a heterogeneous group of RBC enzymatic disorders with PK and G6PD deficiencies being the most common lesions [8]. While G6PD deficiency is the most common enzyme deficiency in humans, clinical phenotypes are quite variable depending upon the severity of the underlying mutation, and are related to residual enzymatic activity [9]. Specific diagnosis of HNSHA is approached through testing activity of enzymes involved in glycolysis and other RBC metabolic pathways. Unfortunately, no enzymatic abnormality is found in up to 70% of cases, highlighting the need for new approaches to understanding the etiology of this disorder [10], [11]. In order to better characterize HNSHA of unknown etiology we have focused upon proteomic analysis of RBC. We have employed a 2D-DIGE (2-dimensional difference gel electrophoresis) approach to identify changes in expression patterns of RBC cytosolic proteins from anemic patients when compared with those of healthy relatives (parents and siblings) and unaffected controls. The application of a comprehensive, unbiased method to asses relative protein abundance broadens the spectrum of knowledge of the RBC proteome and identifies candidate genes, proteins and pathways that may play a role in abnormal RBC turnover.

**Figure 1:**
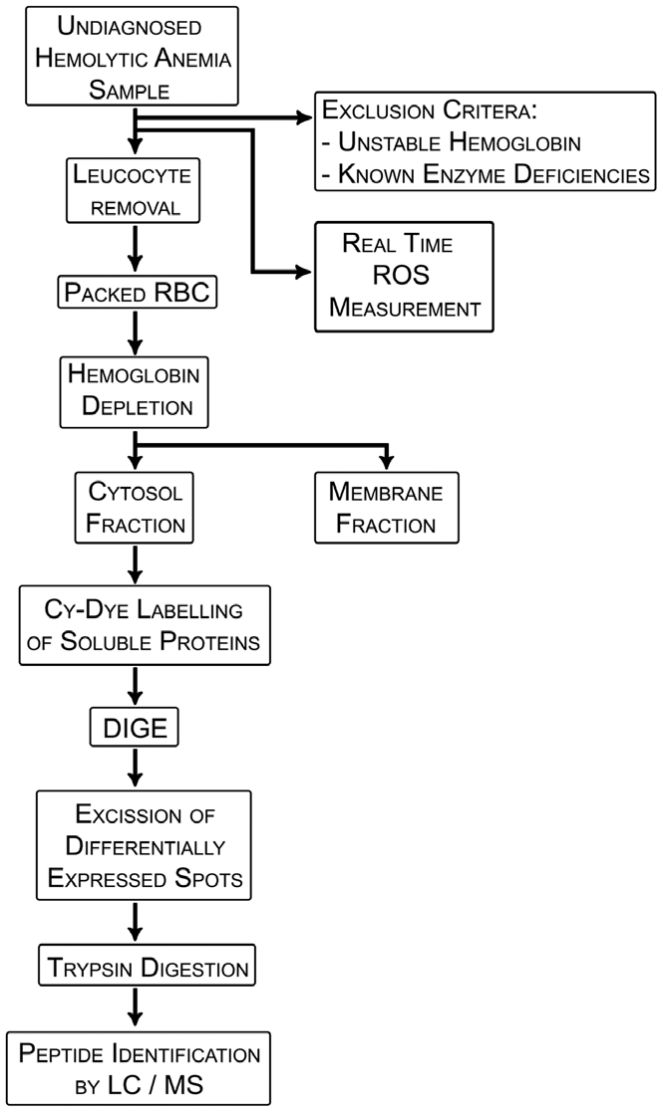
Schematic illustrating the experimental work flow from diagnosis of an anemia patient as HNSHA of unknown etiology to identifications of proteins potentially involved in the pathology of the disease.

## Results

Figure 1 illustrates the experimental scheme for the 2D-DIGE approach used to identify proteins differentially expressed in RBC cytosols. Routine evaluation of samples began with non-proteomic approaches including testing for unstable hemoglobin and evaluation of enzymatic activity for the most common causes of HNSHA (G6PD, PK, and Hexokinase deficiency), if not previously reported in the patient’s record. RBC preparation included passage of cells over microcrystalline cellulose to remove leukocytes followed by hypotonic lysis of RBC, removal of membranes and hemoglobin depletion from the resulting hemolysate. The resulting cytosolic protein fraction was then prepared for DIGE. Gel images were evaluated, and differentially expressed ‘spots’ were excised and processed for protein identification by LC/MS.

Table 1 summarizes relevant laboratory evaluation of the 4 HNSHA patients included in this study: HA09, HA19, HA21 and HA24 (HA for hemolytic anemia). HA09 is a 10-year old male who presented with severe chronic hemolytic anemia at birth, and who remains transfusion dependent. Bone marrow aspirate revealed erythroid hyperplasia with prominent basophilic stippling. Prior workup was negative for hemoglobinopathy, common enzyme defects or RBC membrane abnormalities. Lab values concurrent with sampling for this study included hematocrit (27%), hemoglobin 8.9 g/dl, RBC count 3.09x1012/l, reticulocyte count of 3.5%, bilirubin (11.0 mg/dl) and ferritin (639 ng/ml) levels. Because of the patient’s transfusion dependence, the sample was obtained at the nadir just prior to transfusion (in this case 6 weeks following the most recent transfusion) to maximize the fraction of patient derived RBC for analysis. Glucose-6-phosphate dehydrogenase (G6PD), pyruvate kinase (PK), hexokinase (HK), gluthathione peroxidase (GPx), glutamate-oxaloacetate transaminase (GOT) and glucose phosphate isomerase (GPI) activity as well as a screening test for pyrimidine 5' nucleotidase (P5'N-1) were evaluated to rule out known enzyme defects. Reduced Glutathione (GSH) level was normal. Lacking a clear diagnosis, this sample was selected for proteomic analysis comparing HA09 (patient), his mother, one unrelated control, and a common pooled control sample. The same, pooled control sample (designated ‘standard’) was used in each subsequent HA DIGE experiment as a common control for comparison between experiments.

**Table 1:** Sample characterization (Blood Parameters and Enzyme Assays).

	Normal Range	HA09**	HA19	HA21	HA24**
Age (years)		9.75	2	16.25	0.5
Sex		m	f	f	f
	**Blood Parameters**				
Hemoglobin (Hb)/(g/dl)	m: 13–18f: 12–16	8.9	7.3	12.7	7.4
Hematocrit (HTC)/(%)	m: 45–52f: 37–48	26.9	23	34	22.3
Red cell count (x 10^12^/l)	m: 4.2–5.9f: 3.8–5.5	3.09	3.1	3.57	2.49
Reticulocytes (%)	m: 0.92–2.71f: 0.61–2.2	3.45	11.6	18.4	6.21
Unstable Hemoglobin	0 = negative1 = positive	0	0	0	0
	**Enzyme Activity (UI/g Hb)**				
Pyruvate Kinase	11.1– 18.9	13	23.2	11.8	5.3
G6PD	7.9 – 16.3	9.8	25.9	14.9	ND
Hexokinase	1.02– 2.54	1.62	5.17	1.9	3.45
GPI	38.8– 82.2	49	86.3	60.6	ND
TPI	1317– 2905	ND	1602	1387	ND
Glutathione Peroxidase	21.3– 40.3	30.2	ND	44	ND
Reduced Glutathione	4.5 – 8.7	6.14	7.78	ND	8.19

** blood transfusion prior to drawing blood: HA09 42 days, HA24 37 days.

m: male.

f: female.

UI: International Unit.

G6PD: Glucose -6- Phosphate Dehydrogenase.

TPI: Triose Phosphate Isomerase.

GPI: Glucose Phosphate Isomerase.

ND: No Data.

The complete panel of enzyme assays performed on all samples in this study including controls can be found in Supplementary Table 1.

HA19 is a 2-year old female who presented with chronic, hemolytic anemia accompanied by splenomegaly, leucopenia and thrombocytopenia. Initial work up was negative for common enzyme deficiencies (G6PD and PK), hemoglobinopathy or membrane defects, and there was no family history of chronic anemia. Lab values concurrent with sampling for this study include hematocrit of 23%, hemoglobin of 7.3, RBC count of 3.1x10^12^/l and reticulocytes of 11.6%. Evaluation in our lab revealed no abnormalities in G6PD, HK, PK, GOT, GPI, GPx, triose phosphate isomerase (TPI) or phosphoglycerate kinase (PGK), with GSH in the normal range (Table S1). Because no diagnosis for the etiology of anemia was made, this sample was selected for proteomic analysis comparing HA19 (patient), both parents and one unrelated control sample.

HA21 is a 15-year-old female with mild chronic anemia of unknown cause and a history of a previous acute hemolytic event. Prior evaluation showed no evidence of common enzyme deficiency (G6PD, PK, HK, GPI, TPI and GPx reported as normal), hemoglobinopathy or membrane defect, with normal osmotic fragility. Lab values concurrent with sampling for this study include hematocrit of 34%, hemoglobin of 12.7 g/dl, RBC count of 3.57x10^12^/l and reticulocytes of 18%. Enzyme analyses in our laboratory were negative for deficiencies, but showed elevated GPx in both the patient and her mother. In the absence of an identified defect, proteomic analysis was performed comparing HA21 (patient), her mother and two unrelated control samples.

HA24 is a 6-month old female with severe, chronic anemia requiring transfusion approximately every 5 weeks. As with HA09, a sample for analysis was obtained at the nadir, just prior to transfusion for analysis. At the time of sampling, hematocrit was 22.3%, hemoglobin 7.4 g/dl, RBC count 2.49x10^12^/l and reticulocytes 6.21%. Prior workup for common enzyme deficiencies, hemoglobinopathy and membrane defects were negative. HK activity and GSH levels, were in the normal range. Despite a report of normal PK activity as part of the patient’s original evaluation, enzyme analysis in our laboratory was suggestive of PK deficiency, particularly considering the patient’s reticulocyte count and history of transfusion, with patient, mother and father all showing decreased activity (Table S1). Despite this ambiguity, proteomic analysis was begun in this case comparing HA24 (patient), mother, father, sister and one unrelated control. Subsequent sequence analysis of the PKLR gene revealed that HA24 is homozygous for the R479H mutation [12]; the parents are heterozygous carriers, while the sister is unaffected. A subsequent western blot analysis utilizing a PK specific antibody demonstrated low, but detectable PK expression in the patient, and intermediate expression in both parents compared to unaffected controls (Figure S1). Proteomic analysis of these samples was completed, providing a comparison of differential protein expression in the context of a defined molecular cause of HNSHA.

### Dige Analysis

Table 2 provides an overview of DIGE experiments, including a summary of the number of spots detected on gels from each sample set after spot filtering, and the number of filtered spots differentially expressed by analysis of variance (ANOVA). We excluded spots from further evaluation when they were not matched correctly between the gels, when spot intensity appeared too low to allow identification by mass spec, and when a spot contours drawn by software corresponded to a smear rather than a spot with defined boundaries. A total of 243 spots were picked and a total of 213 proteins were identified (several proteins being repeatedly identified) when pooling data from all four experiments (Table S2). 21 of these proteins have not been previously identified in RBC proteome databases [13], [14], [15] (listed in Table S3). Despite good resolution on 2-D gels, proteins with similar molecular weight and isoelectric point (pI) comigrate, resulting in identification of more than one protein in some spots analyzed.

**Table 2:** Summary of spots detected by Same Spots Software.

Spots	HA09	HA19	HA21	HA24
Total number	963	656	956	1042
Differentially expressed (ANOVA≤0.05)	488	411	687	580
Excluded	291	256	477	471
Included	197	155	210	109
Picked	48	48	99	48
w/o ID	7	1	1	2
Proteins identified	53	63	145	72

Total number: Number of spots detected by Same Spots Software after filtering (area size and minimum volume).

Differentially expressed: Number of spots where difference of expression (normalized volume) of two samples in the experimental set meet the statistical criteria of ANOVA≤0.05.

Excluded: Number of spots that were excluded.

Included: Number of “Differentially expressed” minus “Excluded”.

Picked: Number of spots picked and trypsinized for mass spec analysis.

w/o ID: Number of spots where no peptide could be identified.

Proteins Identified: Total number of Proteins identified in all spots picked in experimental set. Note that more than one protein can be identified in a single picked spot.

**Figure 2:**
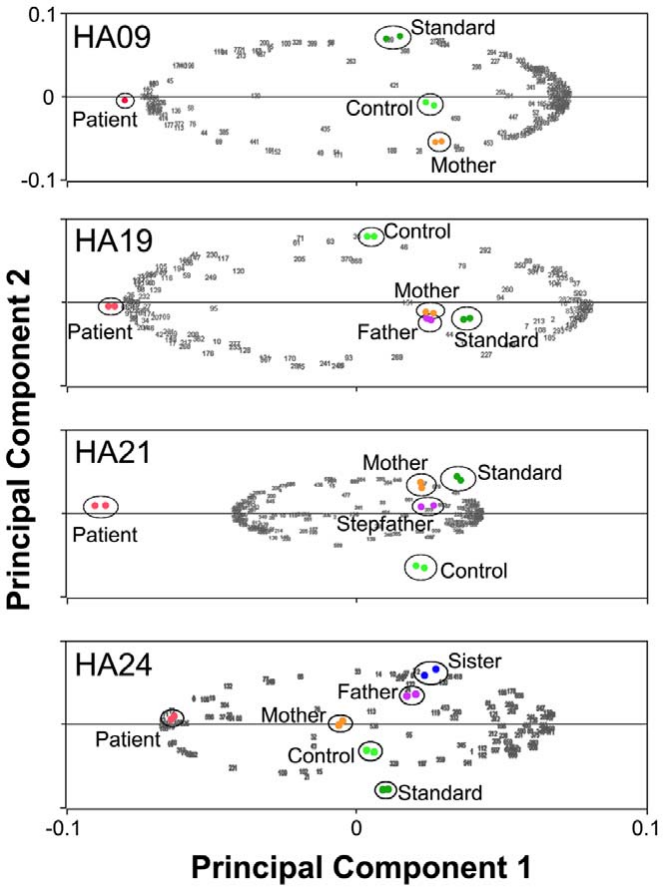
Principal Component Analysis was calculated by Same Spots software. Dot plot of all spots included in analysis (see Table 2) shows that in all four experimental sets (HA09, HA19, HA21 and HA24), replicate samples group closely together and patient samples differ significantly from both familial and unrelated control samples.

As an overall data quality metric, Principal Component Analysis (PCA, Figure 2) of all spots included (Table 2) shows that replicate samples (run with dye reversal) are highly similar in all cases, and that protein expression patterns in HA patient samples are easily distinguished from control samples run in the same experiment. Replicate samples remained tightly grouped when PCA was performed on all detected spots or on all differentially expressed spots (ANOVA). Plotting of the spots showed that in all cases the patient was an “outlier”, separated from more closely grouped family members and controls.

**Figure 3:**
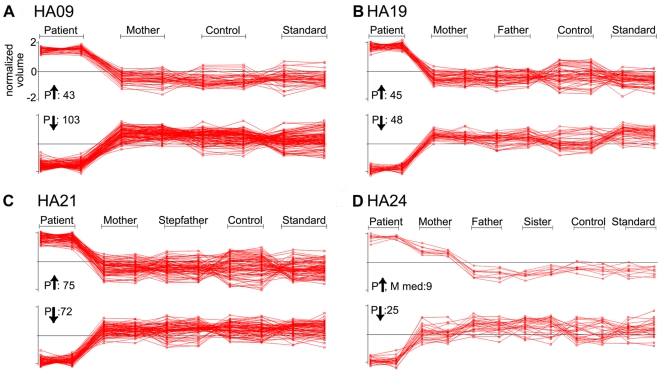
Expression profiles of spots showing up- or down-regulation in patient samples. Of all spots included in the analyses (see Table 2), those showing a distinct up-regulation (P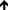, upper panels) or down-regulation (P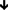, lower panels) in the patient sample when compared with all other samples of the same experimental sets are shown in A: HA09, B: HA19, C: HA21 and D: HA24. Graphic depictions of expression levels include both replicates run per sample comparing normalized volumes.

Assessment of expression profiles of HA09 showed down-regulation of proteins to be more common than up regulation, with more than twice as many spots showing lower expression in the patient (Figure 3, A). For HA19 and HA21 we found a comparable number of differentially expressed protein spots with elevated or reduced expression in the patient versus controls (Figure 3, B-C). 25 spots showed reduced expression in HA24 compared to control samples in this set (Figure 3, D, lower panel). We found 9 spots with increased expression in the patient accompanied by intermediate expression levels in the mother (Figure 3, D, upper panel). In each DIGE comparison, we observed several protein spots with expression intensities for one or both parents intermediate between patient and control samples (as in Figure 3, D above), a pattern that might be expected if parents are heterozygous for a polymorphism affecting expression of the respective protein. Spots with this pattern are considered in more detail below (see Table 3).

**Table 3:** Proteins with parental expression intermediate between patient and unrelated control samples.

ID	Spot rank	Fold change	Gene		Protein
HA09					
	82	- 2.63		G6PD	Glucose-6-phosphate 1-dehydrogenase
				CAP1	Adenylyl cyclase-associated protein 1
	160	- 1.93		G6PD	Glucose-6-phosphate 1-dehydrogenase
	232	- 1.73		PA2G4	Proliferation-associated protein 2G4
	307	- 1.61		PA2G4	Proliferation-associated protein 2G4
HA19					
	89	- 2.14		HSPA8	Isoform 1 of Heat shock cognate 71kD
	94	- 1.92		LOC44091	Similar to 14-3-3 protein epsilon
	231	+1.68		PITHD1	PITH domain-containing protein 1
HA21					
	28	+3.27		EIF2S3	Eukaryotic translation initiation factor 2, subunit 3
	61	+2.58		PSMB4	Proteasome subunit beta type-4 (precursor)
	67	+3.12		ACTR1A	Alpha-centractin
	162	+4.04		VCP	Valosin-containing protein
	200	+2.01		VCP	Valosin-containing protein
	204	-1.93		SOD1	Superoxide dismutase 1, soluble
	258	+1.89		HDHD2	Haloacid dehalogenase-like hydrolase domain containing 2
HA24					
	4	+4.57		EEF2	Elongation factor 2
	13	+4.33		RPSA	Ribosomal protein SA
	14	+3.03		XPO7	Exportin 7
	36	+2.28		BPGM	Bisphosphoglycerate mutase
	43	+2.28		BPGM	Bisphosphoglycerate mutase
	55	+1.86		XPO7	Exportin 7
	97	-1.76		CCT8	Chaperonin containing TCP1, subunit 8
	132	+1.60		ADSL	Isoform 1 of Adenylosuccinate lyase
	159	+1.55		PRDX2	Peroxiredoxin 2
	167	+1.61		PGAM1	Phosphoglycerate mutase 1

ID: Name of sample set (HA09, HA19, HA21, HA24).

Spot rank: Rank of spot as assigned by Same Spots Software depending on fold change (normalized volume) comparing the highest to lowest sample,

Fold Change: Comparison of normalized volume in Patient sample with average of Control and Standard sample.

Gene: HGNC Symbol for coding human gene.

Protein: HGNC Symbol for protein identified.

By superimposing images of Coomassie stained gels from all four experiments including the positions of the picked spots, it was possible to identify differentially expressed spots that were picked in more than one experiment. Across all gels, protein identification was overlapping with the same major protein species present in all cases, and additional peptides from minor species being variably present. As an initial method of protein ID verification the location of the excised spot was compared with the molecular weight (kD) and presumed pI of the identified protein(s). When the discrepancy was > 20% of theoretical molecular mass or > 1 pI unit, it was often found that peptides were derived from the previously picked spot (representing contamination at the level of the spot picking head) and could thus be excluded from further analysis. In some instances we found multiple peptides mapping to a protein larger than the spot location could only be aligned to one part of that protein, suggesting that the protein fragmented prior to gel separation, either as an artifact of processing or a physiologic cleavage prior to sample processing. A representative Coomassie stained 2-D gel is shown in Figure S2.

**Figure 4:**
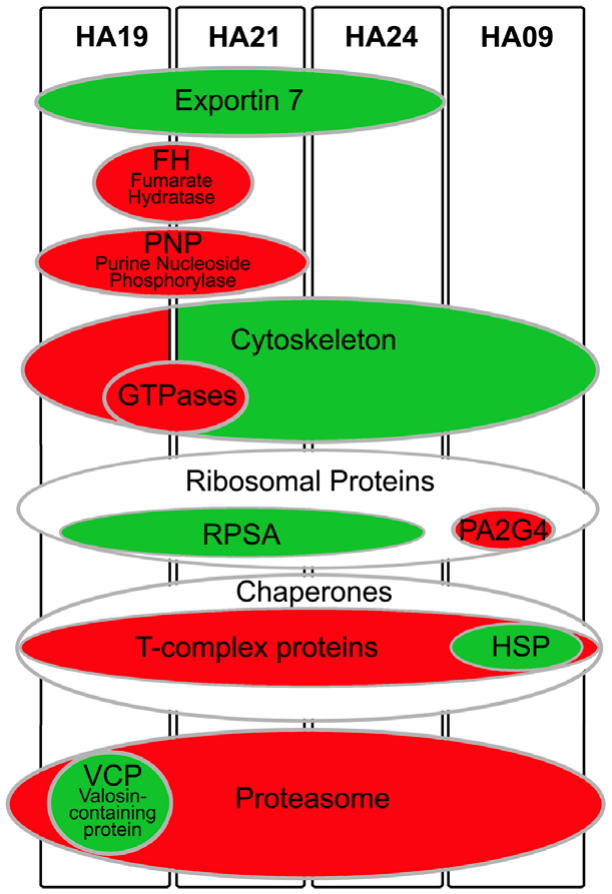
Schematic depiction summarizing proteins and groups of proteins found to be differentially expressed in patients HA09, HA19, HA21 and HA24. Proteins that were expressed at higher levels in patients are represented by green ovals, while down-regulated proteins are represented by red ovals.

Figure 4 presents an overview of this data highlighting proteins that were most highly or most frequently differentially expressed. These include exportin 7 (XPO7), fumarate hydratase (FH), purine nucleoside phosphorylase (PNP), chaperones, cytoskeletal and ribosomal proteins, proteasome subunits and additional proteins involved in protein degradation. Proteins showing lower expression in individual patients are listed in Supplementary Table S4, B.

**Figure 5:**
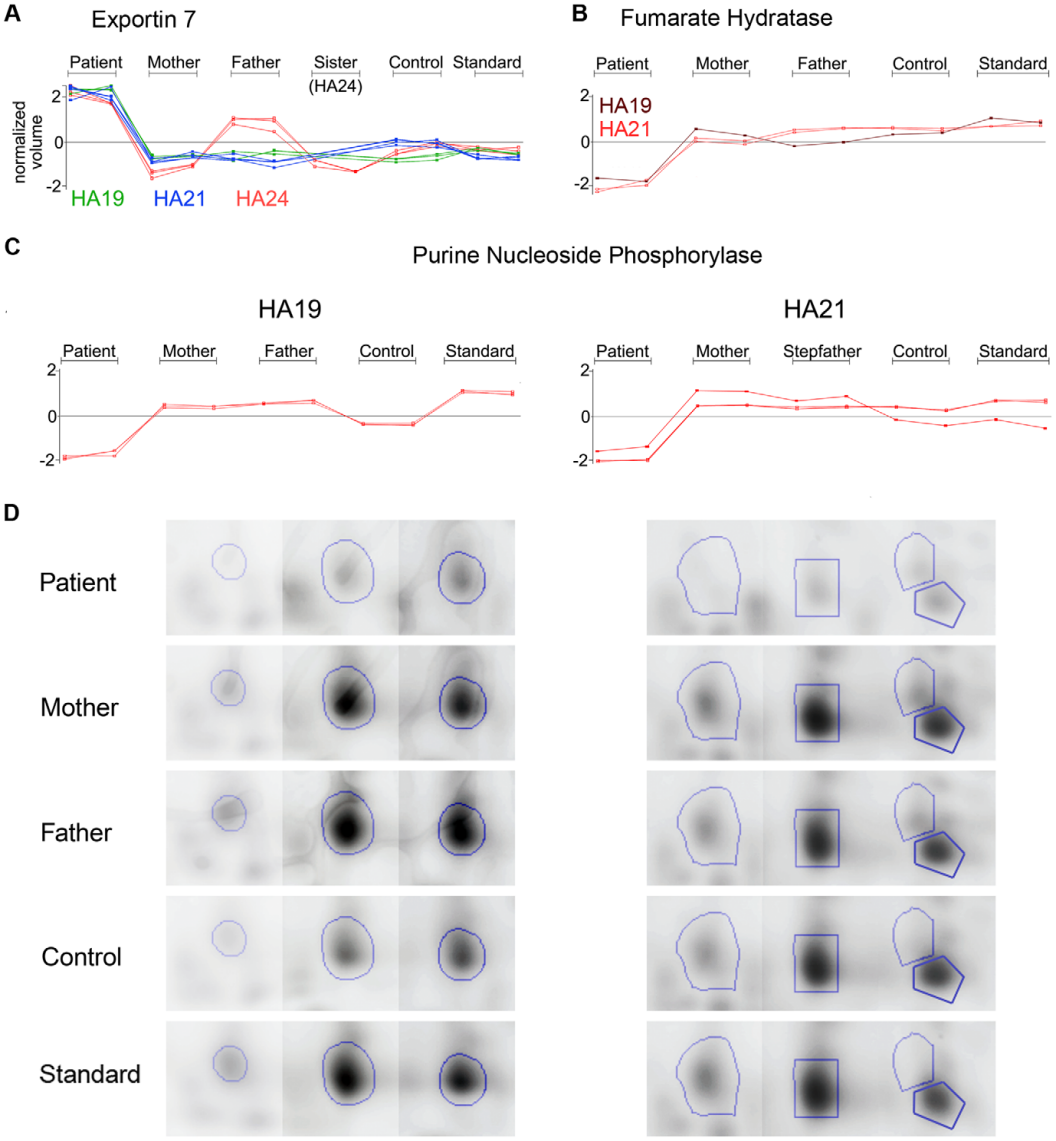
Exportin 7, Fumarate Hydratase and Purine Nucleoside Phosphorylase protein expression. Graphs shows expression levels of Exportin 7 (A), Fumarate Hydratase (B) and Purine Nucleoside Phosphorylase (C) in normalized volume for both replicates run per indicated sample. Included are all spots in which the indicated proteins were identified as the predominant protein. D: Images of gel sections (three for HA19, left panel; four images for HA24, right panel) were merged to show the area where spots were excised. Blue lines enclose spot areas.

XPO7 is a member of the importin-beta superfamily of nuclear transport receptors thought to cycle between the cytoplasm and the nucleus [16]. XPO7 was identified in multiple co-migrating spots in three experiments (Figure 5, A and Figure S3) presumably representing isoforms with distinct post-translational modifications. Patients HA19, HA21 and HA24, all showed consistently increased levels of XPO7 relative to control samples.

The tricarboxylic acid cycle (TCA) enzyme fumarate hydratase, which normally localizes to mitochondria, was unexpectedly found to be present in RBC cytosol. A possible explanation for the presence of this enzyme would be as a component of residual mitochondria in circulating reticulocytes. In patients HA19 and HA21, this explanation appears incorrect, as fumarate hydratase protein was reduced more than 2 fold in these patients compared to controls (Figure 5, B and Figure S4) despite these patients having the highest reticulocyte counts among samples examined.

Purine nucleoside phosphorylase (PNP) was identified in several spots in all experiments. PNP deficiency is a rare autosomal recessive disorder characterized by autoimmunity that may include hemolytic anemia [17], [18]. Patients HA19 and HA21 both show lower PNP abundance in several spots when compared to control samples. Interestingly, the extent of decreased expression varies among the spots: in HA19 from 3.1 fold to 7.7 fold and in HA21 from 2.23, to 9.1 fold, respectively (Table S4, B), while the overall expression pattern remains similar (Figure 5, C, D).

Abundance of specific cytoskeletal proteins differed in all four patients (Supplementary Table S5–and Figure 4) compared to controls. Actin was increased in patients HA09 and HA24, while tubulin was increased in HA21, and a subunit of the dynactin/dynein complex (ACTR1A) [19] showed higher levels of expression HA09. Expression of an actin filament capping protein (CAPZB), a regulator of actin filament growth [20] was found to be lower in HA19. A small GTPase, RhoA, a regulator of actin dynamics [21] showed lower expression in HA19 and HA21. We also found diminished expression levels in these patients for two GDP dissociation inhibitors that influence the GDP-GTP exchange of rab GTPases, known to be involved in vesicle trafficking [22].

Two proteins thought to be involved in ribosome assembly and/or stability were differentially expressed in all four patients. Proliferation-associated protein 2G4 (P2G4) is part of a pre-ribosomal ribonucloeprotein complex and has been implicated in growth regulation in human fibroblasts [23] and cancer cells [24], [25]. In HA09 PA2G4 expression is reduced 1.5 to 1.61 fold (Table S4, B). Ribosomal protein SA (RPSA), also called Laminin-receptor 1, was more highly expressed in HA19, HA21 and HA24 (Table S4, A). In addition to being a receptor for laminin, RPSA is required for assembly and stability of the 40S ribosomal subunit.

**Figure 6:**
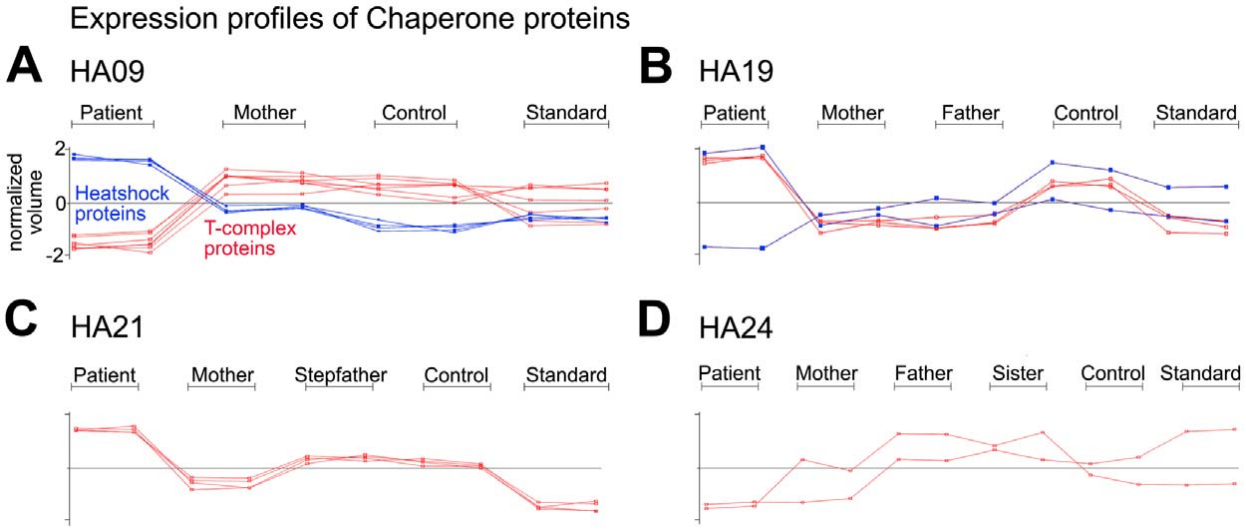
Expression profiles of spots predominantly containing Chaperone proteins are shown (see also Supplementary Table S6). Profiles for HA09 are depicted in (A), HA19 (B), HA21(C) and HA24 (D). Graphs show expression levels in normalized volume for both replicates run per indicated sample. Red lines represent expression patterns of T-complex protein subunits; Heatshock proteins are in blue.

Chaperones were also differentially expressed in all patients (Table S6 and Figure 4). Conspicuously, in HA09 multiple T-complex subunits (TCP) showed reduced expression, while several heat shock proteins (HSP) were present in higher amounts when compared with control samples (Figure 6, A). In HA19 and HA21 several TCP subunits were increased; HA19 also had decreased expression of a single heat shock related protein (HSPA8, Figure 6, B). Several chaperone proteins showed reduced expression in HA19 and HA24, although only two spots in HA21 and one spot in HA24 met the threshold for a change above 1.5-fold.

**Figure 7:**
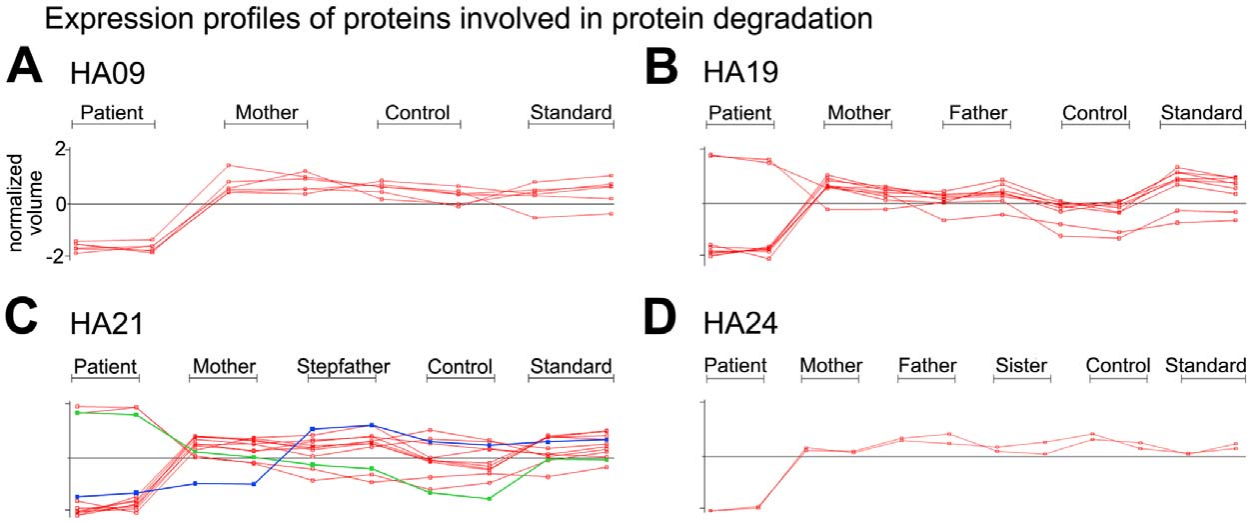
Expression profiles of proteins involved in protein degradation (see also Supplementary Table S7). Graphs show expression levels in normalized volume for both replicates run per indicated sample (A: HA09, B: HA19, C: HA21 and D: HA24). Two spot expression patterns in HA21 differ from the others: PSMB4 is expressed at reduced levels in both patient and her mother (blue line) and PSMC5 is expressed at increased levels in the patient (green line).

A prominent common pattern observed in all patient samples was reduced abundance of multiple proteasome subunits as well as reduced expression of additional proteins involved in protein degradation (Figure 7, Table S7), indicating differences in protein turnover between HA patient RBC and control samples. There was a single exception to this pattern among the proteasome subunits (increased expression of PSMC5 in patient HA21, Figure 7, C green line). In HA19 and HA21 two additional proteins associated with the proteasome, thioredoxin-like 1 [26] and PITHD1 (C-terminal proteasome-interacting domain of thioredoxin-like) domain-containing protein 1, showed higher expression levels compared to controls.

**Figure 8:**
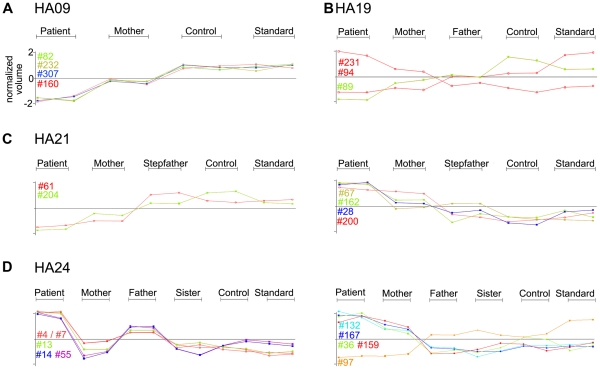
Examples of protein expression patterns where one or both parents are intermediate between the levels measured in the patient sample and those measured in the controls (see also **Table 3**). Shown are Graphs (in normalized volume) for both replicates run per indicated sample in experimental set HA09 (A), HA19 (B), HA21 (C, left panel: patient lower expressing, right panel: patient higher expressing) and HA24 (D, left panel: patient lower expressing, right panel: patient higher expressing). Numbers (#) refer to Spot ranks in Table 3.

In order to focus upon protein expression changes that could play a role in etiology of HNSHA, we searched for spot patterns where the volume measured in the patient sample was most distinct from the control samples, and one or both parents showed intermediate expression. We reasoned that such a pattern might be indicative of recessive inheritance of a lesion for which both parents were heterozygous (Table 3 and Figure 8)–particularly in those cases where expression was decreased in the HA patient sample. Such patterns of possible recessive inheritance were observed in patients HA09, HA21, and HA24.

In HA09 (Figure 8, A) two spots containing G6PD (#82 and #160) and two spots identified as PA2G4 (#232 and #307), were identified with the lowest expression measured in the patient sample, with mother or mother and father showing intermediate expression relative to unrelated controls. Expression profiles for HA19 are shown in Figure 8, B. 14-3-3 epsilon (#94) showed reduced expression in both the patient and mother, and HSPA8 (#89) showed low expression in the patient with intermediate expression in both parents. PITHD1 (#231) showed increased expression in the patient, and intermediate expression in the mother. In HA21, proteasome subunit PSMB4 (#61) and superoxide dismutase 1 (#204) showed low expression in the patient and intermediate expression in the mother (Figure 8, C, left panel). Four spots with higher expression in the patient and intermediate expression in the mother compared to controls (Figure 8, C, right panel) were identified: EIF2S3 (eukaryotic translation initiation factor, #28), ACTR1A (alpha-centractin, #67), and two spots both identified as VCP (valosin containing protein, #162 and #200).

In HA24, CCT8 (T-complex chaperone subunit, #97) was identified as a protein with low expression in both the patient and mother (Figure 8, D, left panel). 8 spots with increased expression in the patient and intermediate expression in one parent were identified: RPSA (ribosomal protein SA, #13), XPO7 (exportin 7, #14 and #55), EEF2 (elongation factor 2, #4) BPGM (bisphosphoglycerate mutase, #36 and #43), ADSL (adenylosuccinate lyase, #132), PRDX2 (peroxiredoxin 2, #159) and PGAM1 (phosphoglycerate mutase 1, #167).

## Discussion

Known causes of hereditary non-spherocytic hemolytic anemia (HNSHA) include lesions affecting enzymes involved in glycolysis, the pentose phosphate pathway, gluthathione metabolism, and nucleotide homeostasis [11], [27]. However, arriving at a molecular diagnosis in patients with HNSHA remains a challenge, with the majority of clinical evaluations failing to identify a causal lesion–indicating that novel approaches to diagnosis are required. In this study, we tested whether a quantitative proteomic approach utilizing 2-D gels could be used to discriminate between anemic and control RBC proteomes in four patients with HNSHA, and provide identification of specific proteins and pathways that may be involved in pathogenesis in individual patients. We found several proteins that might serve as biomarkers for HNSHA, however, our findings are preliminary and further validation is needed in order to establish a clear link between those proteins and the etiology of anemia.

Each sample was evaluated on 2 separate gels using a dye reversal approach [28], [29] to avoid systematic bias in labeling or detection of protein spots, and to allow each patient sample to be compared against parental samples (when available) within the same gel. Principal component analysis showed a close grouping of all replicate samples with patient samples clearly distinct from controls and healthy family members (Figure 2). Several spots were picked independently as differentially expressed in more than one patient/family comparison. In all such instances, the same protein(s) was identified, showing that our peptide identification was reliable and reproducible (Figure S2 and corresponding Tables S8, S9, S10, S11, S12, S13, S14, S15). Low abundance spots were not chosen for picking because we could not reliably identify them based upon the sensitivity of our mass spec. In total we identified 213 proteins in 243 spots picked for analysis (Table 2), 21 of which are not included in previously published RBC proteome databases (Table S3) [13], [14], [15].

### Reticulocytosis

An obvious difference between control and HNSHA RBC samples is the increased reticulocyte count in patient samples. HA09 and HA24 differ from HA19 and HA21 in that they are both transfusion dependent–indicating their ability to produce RBC is more significantly impaired. Because reticulocytosis by itself will increase the abundance of protein species that are normally lost during erythroid maturation, differential expression of mitochondrial proteins, components of protein synthetic machinery and heme biosynthetic pathway, as elucidated in a recent analysis of a mouse hemolytic anemia model [30], may simply be a reflection of reticulocytosis and not be directly related to the underlying pathology causing hemolysis. While reticulocytes were elevated in all HA patients examined relative to controls, HA19 and HA21 had the highest reticulocyte counts at 11.6% and 18.4%, respectively–and these two samples had the highest number of spots showing increased expression relative to controls (Table S4, A). Included in the list of proteins with increased expression in both patients were several involved in protein synthesis, the heme biosynthetic enzyme uroporphyrinogen decarboxylase (UROD), autophagy related 3 (ATG3) protein that is an important regulator of mitochondrial homeostasis [31], and several subunits of the TCP1 chaperone complex that aids in folding actin and tubulin among other targets [32], [33].

Exportin 7 (XPO7), a protein involved in shuttling macromolecules across the nuclear envelope [16], was also increased in both patient samples. Interestingly, XPO7 was recently identified as a protein essential for proper erythroid maturation in mammals [34]. Deficiency of XPO7 could thus presumably lead to inferior RBC integrity caused by a defective maturation process, and low expression of this protein could play a role in the etiology of hemolytic anemia.

RPSA, a protein important for ribosome assembly and stability of the 40S ribosome, was increased in patients HA19, HA21 and HA24. RPSA, also named laminin receptor 1 [35], shows increased expression in cancer cells where expression correlates with invasiveness and metastasis [36].

Eukaryotic elongation factor 2 (eEF2), a protein that mediates mRNA translation at ribosomes, was increased in patients HA21 and HA24 (Tables S12, S13, S14 and S15). In contrast, HA09, the patient with the lowest reticulocyte count, showed decreased expression of many of the same proteins when compared with control samples (Table S4, B).

Patient HA09 is also transfusion dependent, and the combination of low endogenous RBC production in a background of normal, transfused RBC likely explains why this sample had fewer proteins with significantly increased expression. This supports the conjecture that the observed increase in abundance of many proteins in HA patient samples is a marker of the relative increase in reticulocyte count.

### Protein Quality Control

Endogenous protein quality control is a critical process, especially for a cell such as the erythrocyte with limited capacity to replace or repair damaged proteins. The degradation of damaged or defective proteins is an important homeostatic mechanism to avoid protein aggregation, membrane damage and cell death [37]. The observation of differential expression of proteins involved in endogenous protein quality control and degradation pathways in HA patient samples may be related to increased reticulocytosis (as discussed above), or may be a response to an increased load of misfolded or damaged proteins that accumulate in developing RBC of HA patients. Chaperones, like heatshock proteins (HSP), can refold polypeptides and salvage them; when proteins are irreparably damaged they ensure their degradation by the ubiquitin-proteasome pathway [38]. HSP play a key role in the triage of proteins with oxidative damage [39], [40], are normally downregulated during erythroid maturation [41], but show increased expression in the context of RBC defects such as thalassemia [42]. Interestingly, we identified multiple differentially expressed protein species in all HA patient samples that are components of both endogenous protein quality control and protein degradation systems (Figure 4, Tables S6, S7).

Reduced expression of multiple subunits of the 26S proteasome as well as other proteins of the ubiquitin-proteasome system was found in all HA patient samples (Table S7), and represents perhaps the clearest evidence of phenotypic similarity between samples. Among the 4 HA samples analyzed, there was a divergence in the pattern of differential expression of HSP and chaperone proteins–most notably HA19 and HA21 showed increased expression of T-complex chaperone proteins while HA24 and in particular HA09 showed decreased expression of T-complex proteins and increased expression of several HSP (Table S6). The cytosolic TCP-1 ring complex (TRiC; also called CCT, for chaperonin containing TCP-1) consists of 8 subunits [43], assists in the folding of numerous proteins, and is often found associated with nascent polypeptides at ribosomes [44]. In addition, we found increased expression of an additional protein associated with the ubiquitin-proteasome- system that also exhibits chaperone activity, valosin containing protein (VCP) [45], in HA21 (Figure 7, C). Increased expression of VCP has been shown to counteract the toxicity of both bortezomib on proteasome activity and geldanomycin on HSP function [46]. Higher expression of HSPs and VCP may be a compensatory response to decreased activity of the proteasome machinery; HSPs and VCP may function together to enhance chaperone activity and avoid accumulation of misfolded proteins. Compounds that increase chaperone activity have potential as therapeutic agents by increasing the fraction of properly folded target proteins during RBC development.

### Cytoskeleton

During maturation from reticulocyte to erythrocyte extensive reorganization of the cytoskeleton and membrane occur in order to maximize cell malleability and shear resistance. Microfilamants and microtubles are essential for cell motility and the extrusion of the nucleus [47]; tubulin and actin are degraded by the ubiquitin – proteasome pathway and are absent from the cytosol of mature RBC in mice [48]. We found both cytoskeletal proteins to be increased in HA patients compared to controls. We also observed a lower abundance of RhoA and RhoC as well as Rab GDP dissociation inhibitor 1 and 2 (GDI 1/2) in patients HA19 and HA21. Rho GTPases control actin dynamics [49], Rab GTPases coordinate vesicle transport [22] and membrane trafficking [50]. Diminished availability of regulatory GTPases can interfere with membrane reorganization and vesicle trafficking and could alter actin dynamics in the maturation process from reticulocyte to mature RBC and thus give rise to RBC with shortened life span.

### Furamate Hydratase

Fumarate hydratase (FH) is a tricarboxylic cycle (TCA) enzyme with reduced expression in patients HA19 and HA21 (Figure 5, B, Supplementary Figure S4) even though both patients had a high reticulocyte count (Table 1). Because of its mitochondrial function and localization, FH is not expected to be part of the mature erythrocyte’s proteome, yet it was previously identified in RBC proteome studies [13], [14], [15], suggesting an unknown function outside of the TCA cycle. A relationship between heme synthesis, heme catabolism and FH activity has been identified in tumor cells where loss of FH activity is synthetically lethal with defects in heme catabolism [51]. In this context, heme synthesis utilizes TCA substrates proximal to FH, overcoming a TCA cycle defect allowing production of required NADP and maintenance of mitochondrial membrane potential. Heme catabolism provides a sink allowing this bypass to continue. The function of FH in the cytosol of mature RBC, and the functional impact of the decline in FH expression found in HA19 and HA21 is unclear. One possibility is that during RBC development, reduced FH activity slows distal metabolite flow in the TCA cycle, thus making succinyl-CoA available for heme biosynthesis. The observed reduction in FH protein in peripheral RBC of two patients in this study may thus be a downstream consequence of altered development. This would indicate an even more pronounced reduction in FH expression in marrow progenitors of these patients.

### Purine Nucleoside Phosphorylase

HA19 and HA21 also showed reduction in expression of purine nucleoside phosphorylase (PNP, Figure 5, C and D), an enzyme highly expressed in early erythroid precursors and erythrocytes, that is an integral part of the purine salvage pathway (http://biogps.org/#goto=genereport&id=4860). PNP deficiency is life-threatening condition resulting in severe T-cell dysfunction associated with hypouricemia [52], [53], [54] and anemia [17], [55], [56]. Erythrocytes from patients with PNP deficiency show a depletion of GTP and disruption of nucleotide pools [57]. While neither HA19 nor HA21 show evidence of immune dysfunction characteristic of PNP deficiency, the degree of reduction of PNP expression in these patients is dramatic, and may be related to the etiology of their hemolytic anemia.

### Limitations of the DIGE approach

Although outside screening had been negative, we suspected patient HA24 could be PK deficient based upon borderline low PK activity and below control values in both parents when assayed in our laboratory (Table S1). Sequencing confirmed that HA24 was homozygous and both parents heterozygous for the R479H mutation, previously identified as a cause of severe HNSHA [12], [58]. The mutation, located at the end of exon 10, appears to interfere with mRNA splicing, decreasing protein production, resulting in PK deficiency [59]. Low PK activity in HA24 was corroborated by low protein on Western-blot analysis (Figure S1). However, we did not independently identify PK as a differentially expressed protein using the DIGE approach, providing a clear example of the limitations of this methodology.

Lack of sensitivity of the DIGE approach reflects a number of technical choices/challenges in carrying out this study. First, we limited analysis to cytosolic proteins, excluding the membrane fraction because of poor resolution of membrane proteins on 2D gels. Second, we ran the cytosolic fractions over DEAE in order to remove hemoglobin and thus increase sensitivity for lower abundance cytosolic proteins. It is likely that some cytosolic proteins were removed in this step along with hemoglobin. Finally, proteins with extreme isoelectric points (below 4.0 and above 8.5) were outside the range of resolution of our 2D gels. While the above comments relate to technical limitations of 2-D DIGE, there are additional limitations inherent in experimental design when comparing single clinical samples against related and unrelated controls. Here, in order to allow meaningful statistical comparisons, each sample was run twice using a dye-reversal strategy that also corrects for biases in the protein labeling or fluorescence detection steps. While this approach controls for experimental variations, when differential protein expression between patients and controls is observed, we cannot discriminate between anemia-related differential protein expression and differences in expression that represent a polymorphism between the patient and controls that is not disease associated.

In addition to gel separation problems, we encountered single proteins that ran in several spots next to each other, raising problems for relative quantification. Examples include G6PD, BPGM, eEF2, XPO 7 (Figure S3), FH (Figure S4) and PNP (Figure 5, D). While changes in molecular mass were minor, the isoelectric points were different enough to create distinct spots. Modifications such as phosphorylation, acetylation, oxidation and proteolytic cleavage are presumed to underlie the observed differences in isoelectric point [60]. Shifting between different isoforms creates a situation where overall expression of a protein might not be changed, but volume measured could shift between spots. Finally, altered protein expression does not necessarily translate to changes in measured enzymatic activity. Bisphosphoglycerate mutase (BPGM) is an example for this disconnect between measured expression levels and enzyme activity. BPGM catalyses the synthesis of 2,3-disphosphoglycerate (2,3-DPG), that binds to and regulates hemoglobin oxygen affinity. We found BPGM protein levels were increased in samples from patient HA24 and her mother (Table 3, Figure 8). However, when assaying hemolysates derived from these samples, we found no corresponding changes in BPGM enzymatic activity (data not shown).

### Outlook

Evaluation and treatment of patients with hereditary nonspherocytic hemolytic anemia continues to be a challenge. By using a proteomic approach to evaluate four patients with this condition we found a common pattern of altered expression of proteins involved in protein quality control and degradation, raising the possibility that interventions to increase chaperone activity may benefit some patients by diminishing accumulation of damaged or misfolded proteins. We also identified several patient-specific alterations in protein expression that require further investigation (Table S4, A, B). While DIGE is a powerful tool to identify differential protein expression, drawbacks of this approach include labor and resource intensity, as well as non-comprehensive nature of the data obtained–in this case failure to identify PK as a differentially expressed protein in patient HA 24. As such, this approach is better suited to identifying qualitative differences between samples, such as altered expression of proteins involved in protein homeostatic systems, rather than as a specific diagnostic tool for individual patients.

## Materials and Methods

### Sample Collection and Ethics Statement

Peripheral blood samples from HA patients, unaffected family members and controls were collected after obtaining informed consent at the referring institution, using consent documentation approved by the Scripps Research Institute IRB (La Jolla, CA) that specifically approved this study. Because each HA patient was a minor, consent documentation was signed by parent/guardian, and, when age-appropriate, by participating patient. Control samples obtained from family members utilized the same informed consent documentation and procedure. Non-familial control samples were obtained from the Scripps Normal Blood Donor pool, again after obtaining informed consent using documentation of the Scripps Research Institute IRB that specifically approved this study. 7–10cc of whole blood was collected into EDTA tubes and shipped overnight on wet ice.

### Packed Red Blood Cells

White cells were removed from whole blood by passage over microcrystalline cellulose columns and washed 3 times in 0.9% saline solution. RBC were frozen at -80°C prior to fractionation for proteomic analysis. Reticulocyte count was assessed using BD Retic-Count™ stain according to the manufacture’s protocol (BD Bioscience).

### Enzyme Assays

Assays to measure activity of pyruvate kinase, hexokinase, glucose-6-phosphate dehydrogenase, glucose phosphate isomerase, triose phosphate isomerase, glutathione peroxidase as well as concentration of reduced glutathione were performed as described by Beutler [61]. Unstable hemoglobin was determined by as described by Carrell and Kay [62]. A screening test for pyrimidine 5' nucleotidase deficiency consisting of measurement of the OD260/280 ratio of a perchloric acid extract of RBC was performed as described by Beutler [61] on sample HA09 due to the presence of basophilic stippling on peripheral smear. The ratio was within the normal range.

### Preparation of Cytosolic Proteins

Packed RBC were thawed on ice by addition of 3 volumes buffer (10 mM TRIS, pH 6.5; 2 mM EDTA; 10 mM dithiothreitol (DTT)) containing proteasome inhibitors (Complete ULTRA, Roche) for hypotonic lysis. After centrifugation, TRIS (pH6.5) was added to a final concentration of 100mM followed by passage over DEAE sephadex A-50 (GE Healthcare) to remove hemoglobin. Following three washes with 100 mM TRIS, pH 6.5, cytosolic proteins were removed from the column with 100 mM TRIS, pH 6.5, 0.5 M NaCl. The resulting protein solution was concentrated and subjected to buffer exchange with 10 mM TRIS pH6.5, 2 mM EDTA using centrifugal filter units (Millipore Ultracel 10k). Protein concentration was measure using BCA™ Protein Assay (Thermo Scientific) following the manufacturer’s protocol.

### Protein Preparation and Labeling for DIGE

400 µg of cytosolic proteins were prepared for 2D electrophoresis using the Ready-Prep 2-D clean up Kit (Bio-Rad Laboratories), proteins were resuspended in 7 M urea (Sigma), 2 M thiourea (Sigma) 2% m/v ASB-14 (G-Bioscience) and 30 mM TRIS; protein concentration was assessed using 2-D Quant Kit (GE Healthcare). 300 µg of each sample were labeled using the Amersham™ 5 nmol CyDye Fluors minimal Dye labeling Kit according to the manufacturer’s protocol (GE Healthcare). We used a dye reversal labeling strategy: each sample was run twice in any given experiment labeled with Cy-3 and Cy-5, respectively. When possible the patient was paired with either parent to run on a gel for direct comparison. An equal amount of each sample in any given experiment was also pooled and labeled with Cy-2 to serve as an internal standard used to normalize expression levels. This also allowed us to compare between gels of a sample set. A standard sample was created as a bridge between experiments by pooling 10 blood samples from healthy donors. This common standard sample was included within each patient-specific DIGE experiment.

### Iso-Electric Focusing (IEF)

Using passive rehydration loading, 300 µl (300 µg pooled sample) were applied to 17 cm 3–10 NL immobilized pH gradient strips (Bio-Rad Laboratories) after adding carrier ampholytes (IPG buffer 3–10 NL, GE Healthcare) and 2 mM DTT (Bio-Rad Laboratories). Labeled proteins were isoelectrically focused using the PROTEAN IEF Cell (Bio-Rad Laboratories). Focusing parameters were 50–250V linear gradient for 1 hour, 250–4000 V linear gradient for two hours, and then hold at 8000V and hold for an accumulation of 56000 V-hours. IEF was temperature and current limited at 25°C and 50 µAmp per strip [63].

### Second-Dimension Polyacrylamide Gel Electrophoresis (PAGE)

Focused IEF strips were reduced and carbamidomethylated for 10 minutes at each step using 10 mg/ml DTT and 25 mg/ml iodoacetamide solutions (Equilibration Solutions I & II, Bio-Rad Laboratories) formulated in a 6 M urea/30% (v/v) glycerol/2% SDS 50 mM TRIS buffer at pH 8.8. The IEF strips were rinsed briefly in SDS-PAGE electrophoresis buffer (25 mM TRIS, 192 mM glycine, 0.1% SDS), and then placed on 10–14% gradient polyacrylamide gels cast in low-fluorescence glass plates (Jule Technologies). We removed 1 cm from the basic end of the strips to accommodate the dimensions of the gel chambers. Each IEF strip was fixed to the gel with warm 0.5% (w/v) agarose sealing solution (Bio-Rad Laboratories). PAGE was run overnight in a PROTEAN IIxi Cell at 14°C at 8 mA/gel for the first hour and 12 mA/gel for 15 hours. Power was continuously supplied until the bromophenol blue front migrated out the end of each gel.

### Gel Documentation

Gels were documented with a Typhoon Trio imager (GE Healthcare). Fluorescence images were analyzed with Progenesis SameSpots software (version 3.2, Nonlinear Dynamics), differentially expressed spots were identified and a pick list was created. Spots with an average normalized volume (ANV)≤5000 or a spot area (SA) below 400 for HA09, SA≤250 for HA19 and SA≤300 for HA21 and HA24 were excluded from analysis based upon difficulty picking and identifying low abundance proteins. Between 50 - 69% of spots remaining had a variance between the sample groups (ANOVA)≤0.05 (see Table 2). Spots were also excluded from analysis when they were not exactly matched between gels or were too close to the edge of the gels (and thus not reliably focused). Spots were sorted by ascending intensity to reduce carry over from robotic gel excision. Gels were subsequently fixed in 10% methanol, 7% acetic acid (Fisher Scientific) and stained with BioSafe Coomassie (Bio-Rad). Selected spots were picked with a ProPicII picking robot (Genomic Solutions), followed by a sequencing grade trypsin digest (Promega) in a ProGest digestion station (Genomic Solutions).

### Identification of Peptides by Liquid Chromatography - Mass Spectrometry (LC-MS)

Gel spot digests in 96-well plates were dried, and resuspended 50 µl/well using 5% (v/v) acetonitrile, 0.1% (v/v) formic acid (Buffer A). An Agilent dual pump system with a 2/6 switching valve was used to inject and elute the tryptic peptide solutions to a Thermo Finnegan LCQ Deca XP MAX ion trap mass spectrometer for protein identification. Agilent pump system components are an 1100 series Quaternary Pump, an 1100 series micro well plate sampler, a 2-position 6-port switching valve, and a 1200 series NanoFlow binary pump. During the sampling phase, 40 µl aliquots were injected to a 5 mm C18 trap column loop on the switching valve. The trapped peptides were then eluted with the NanoFlow pump at 0.5 µl/min to a custom-built in-lab 7 cm C18 (5 µm particle size, Jupiter C18, Phenomenex Corporation) reversed-phase 100 µm I.D. micro column. Peptides were resolved with linear gradient elution using 95% (v/v) acetonitrile, 0.1% (v/v) formic acid (Buffer B) as the organic phase: from 0% to 40% Buffer B over 30 minutes [64]. To minimize sample carry-over, the trap and column were cleaned by a 5-minute wash of Buffer B after every injection. Tandem mass spectrometry was used to characterize the peptides. The three highest abundance MS1 peaks were selected for CID fragmentation and MS2 for sequencing.

### Bioinformatic Processing and Analysis of MS Data

Xcalibur 2.0 SR2 generated RAW files were extracted for MS2 information using RAWXtract version 1.9.9.1 [65]. MS2 files were searched using the SEQUEST algorithm [66]. SEQUEST searches were performed using a concatenated target/decoy non-redundant variant of the human IPI database version 3.33 (available for download at http://www.scripps.edu/chemphys/cravatt/protomap) allowing for differential methionine oxidation (+16 amu) and requiring cysteines to be carbamidomethylated (+57 amu) [67]. SEQUEST data from each gel spot were filtered and sorted with DTASelect2 [68]. Using DTASelect2 spectral counting functions, identifications were checked for protein co-location in gel spots. Additionally, sample carry-over effects (generally due to contamination at the gel picker excision head) could be monitored.

### Evaluation of Data

Peptide identification was validated first by matching location on the gel (pH and molecular weight) with the theoretical isoelectric point (pI) and mass of the identified protein. Superimposing gel images (based on Coomassie stain) from all experiments showed that some spots had been picked in separate experiments, resulting in consistent protein identification(s). Spots with multiple IDs were dismissed when no clear predominant protein could be determined; when spectral counts of peptides from one protein exceeded 60% of total spectral counts in a spot, it was considered to be the predominant protein. Fold change was calculated by comparing measured expression level (normalized volume) of patient to the average of all other samples run in a set. For protein identification, we focused upon spots that showed≥1.5 fold difference (in either direction) between the patient and all other samples run in the experiment combined (“P to all”).

### Western Blot Analysis

Cytosolic proteins were separated on 11% polyacrylamide gels and electro blotted onto nitrocellulose (BioRad, CA). Membranes were blocked in Odyssey® blocking buffer (LI-COR Biosciences). Primary antibodies used were as follows: rabbit polyclonal anti-actin (Sigma, MO), mAb anti PKLR (Santa Cruz Biotechnology, CA). Secondary antibodies used were IRDye® 800 goat anti-mouse and IRDye® 680 goat anti-rabbit, followed by detection with Odyssey® infrared imaging system and analysis with the application software version 3.0.21 (LI-COR Biosciences). This software was also used to perform densitometry analysis to normalize PKLR signal to loading control (actin).

## Supporting Information

Figure S1 Western blot analysis of PK expression in sample set HA24. Hemoglobin depleted RBC lysates from HA24 patient (P), mother (M), father (F), sister (S) and control sample (C) were analyzed for PK expression. Densitometry was calculated as percentage (%) of signal compared to the average of sister and control samples after normalizing to loading control actin.
(TIF)

Figure S2 Prototypical image of a Coomassie stained polyacrylamide gel includes protein localization markers for protein mass in kilodalton (Marker/kD) and isoelectric point (pI). All 243 picked spots were superimposed to create this image; spots picked in HA09 are shown in green, HA19 in purple, HA21 in blue and HA24 in red. Multicolored circles indicate spots that were picked in more than one experiment. Numbers refer to spot ranks in Supplementary Tables 3 and 4 (HA09, green), Supplementary Tables 5 and 6 (HA19, purple), Supplementary Tables 7 and 8 (HA21, blue) and Supplementary Tables 9 and 10 (HA24, red).
(TIF)

Figure S3 Exportin 7 protein expression in HA19, HA21 and HA24. Included are all spots in which Exportin-7 was identified as the predominant protein. A: Graph shows expression levels in normalized volume for both replicates run per indicated sample. B: Images show sections of the gels where Exportin 7 containing spots were excised (blue lines enclose spot areas; upper panel: HA19, middle panel: HA21, lower panel HA24). Pictures were exported from Same Spots and merged to show all relevant spots in one picture.
(TIF)

Figure S4 Fumarate Hydratase expression in HA19 and HA21. Included are all spots in which Fumarate Hydratase (FH) was identified as the predominant protein. A: Graph shows expression levels in normalized volume for both replicates run per sample. B: Images show sections of the gels where FH containing spots were excised (blue lines enclose spot areas; upper panel: HA19, middle panel: HA21). Pictures were exported from Same Spots and merged to show all relevant spots in one image per sample.
(TIF)

Table S1 Complete Summary of Enzyme Assays performed on samples included in this study.
(DOCX)

Table S2 Complete list of all Proteins identified.
(DOCX)

Table S3 Proteins not listed in Red Blood Cell protein databases.
(DOCX)

Table S4 A: Differentially expressed spots with expression level in Patient sample higher compared to controls. B: Differentially expressed spots with expression level in Patient sample lower compared to controls.
(DOCX)

Table S5 Expression of Cytoskeleton Proteins.
(DOCX)

Table S6 Expression levels of Chaperones.
(DOCX)

Table S7 Expression of proteins involved in Protein Degradation/Proteasome/Ubiquitin pathway.
(DOCX)

Table S8 Complete list of all spots picked and proteins identified in HA09.
(DOCX)

Table S9 Complete list of all spots picked in HA19, fold change comparing patient to all other samples run in the experiment.
(DOCX)

Table S10 Complete list of all spots picked and Proteins identified in HA19.
(DOCX)

Table S11 Complete list of all spots picked in HA19, fold change comparing patient to all other samples run in the experiment.
(DOCX)

Table S12 Complete list of all spots picked and Proteins identified in HA21.
(DOCX)

Table S13 Complete list of all spots picked in HA21, fold change comparing patient to all other samples run in the experiment.
(DOCX)

Table S14 Complete list of all spots picked and Proteins identified in HA24.
(DOCX)

Table S15 Complete list of all spots picked in HA24, fold change is comparing patient to all other samples run in the experiment.
(DOCX)
