# Supplementary figures and images for: Assessment of the Red Cell Proteome of Young Patients with Unexplained Hemolytic Anemia by Two-Dimensional Differential In-Gel Electrophoresis (DIGE)
Source: PLoS One. 2012 Apr 3;7(4):e34237. doi: 10.1371/journal.pone.0034237 (PMC3317954; doi:10.1371/journal.pone.0034237)

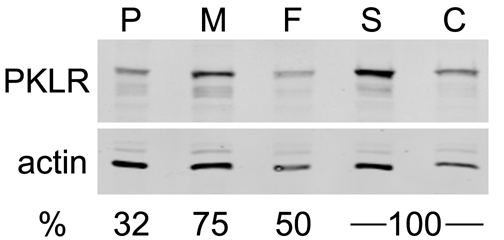

Supplement: Figure S1 — Western blot analysis of PK expression in sample set HA24. Hemoglobin depleted RBC lysates from HA24 patient (P), mother (M), father (F), sister (S) and control sample (C) were analyzed for PK expression. Densitometry was calculated as percentage (%) of signal compared to the average of sister and control samples after normalizing to loading control actin. (TIF) [file pone.0034237.s001.tif]

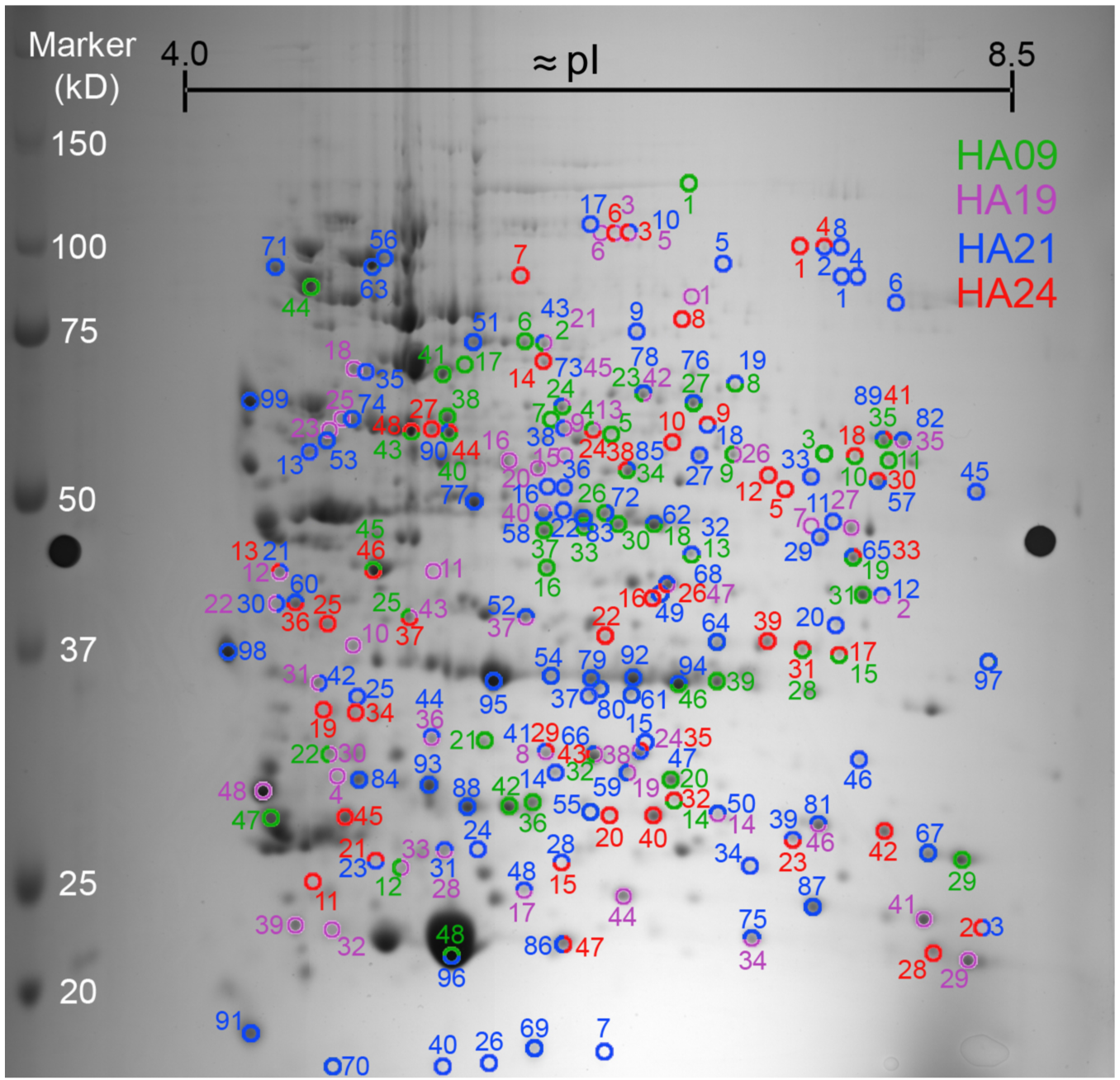

Supplement: Figure S2 — Prototypical image of a Coomassie stained polyacrylamide gel includes protein localization markers for protein mass in kilodalton (Marker/kD) and isoelectric point (pI). All 243 picked spots were superimposed to create this image; spots picked in HA09 are shown in green, HA19 in purple, HA21 in blue and HA24 in red. Multicolored circles indicate spots that were picked in more than one experiment. Numbers refer to spot ranks in Supplementary Tables 3 and 4 (HA09, green), Supplementary Tables 5 and 6 (HA19, purple), Supplementary Tables 7 and 8 (HA21, blue) and Supplementary Tables 9 and 10 (HA24, red). (TIF) [file pone.0034237.s002.tif]

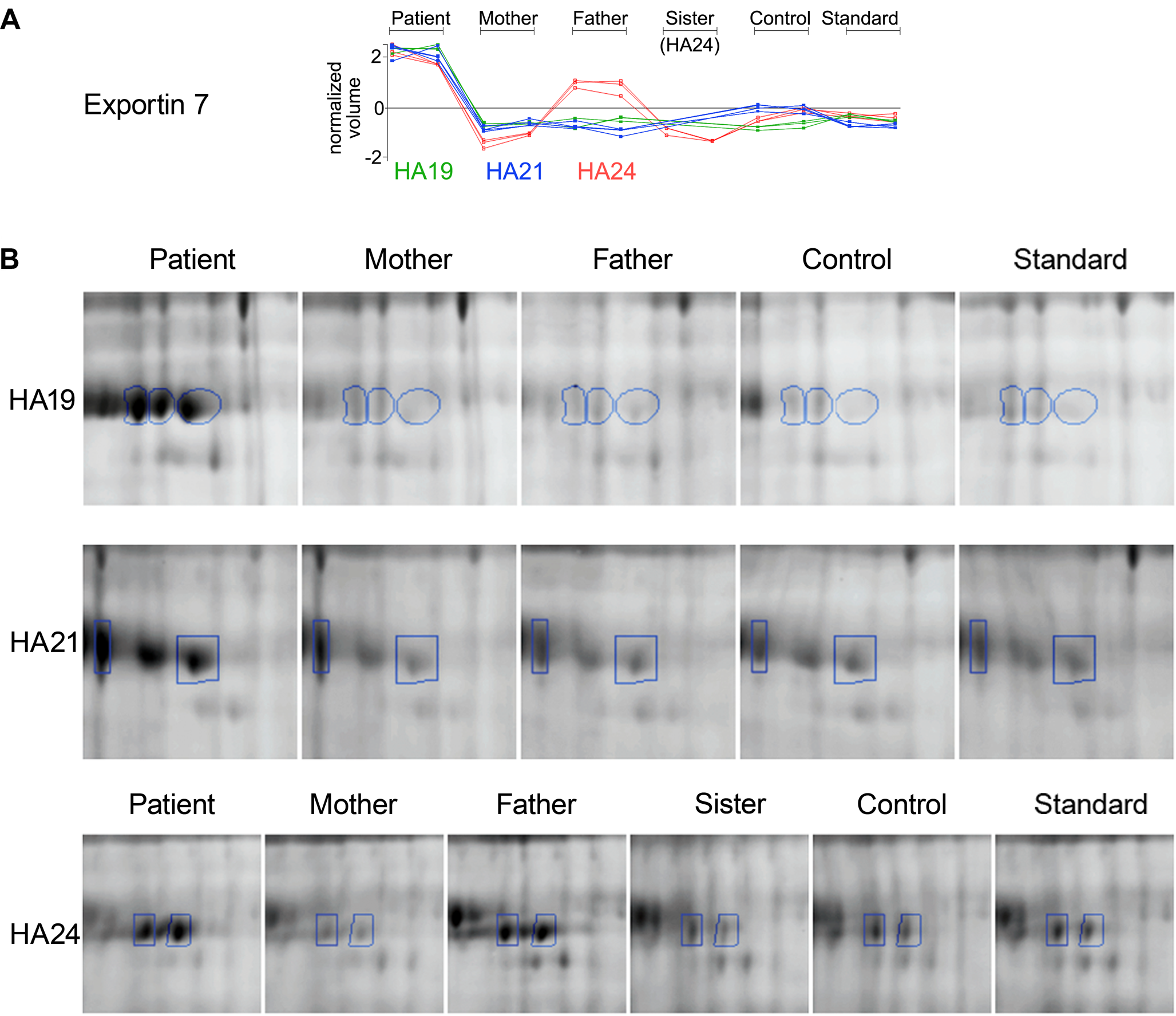

Supplement: Figure S3 — Exportin 7 protein expression in HA19, HA21 and HA24. Included are all spots in which Exportin-7 was identified as the predominant protein. A: Graph shows expression levels in normalized volume for both replicates run per indicated sample. B: Images show sections of the gels where Exportin 7 containing spots were excised (blue lines enclose spot areas; upper panel: HA19, middle panel: HA21, lower panel HA24). Pictures were exported from Same Spots and merged to show all relevant spots in one picture. (TIF) [file pone.0034237.s003.tif]

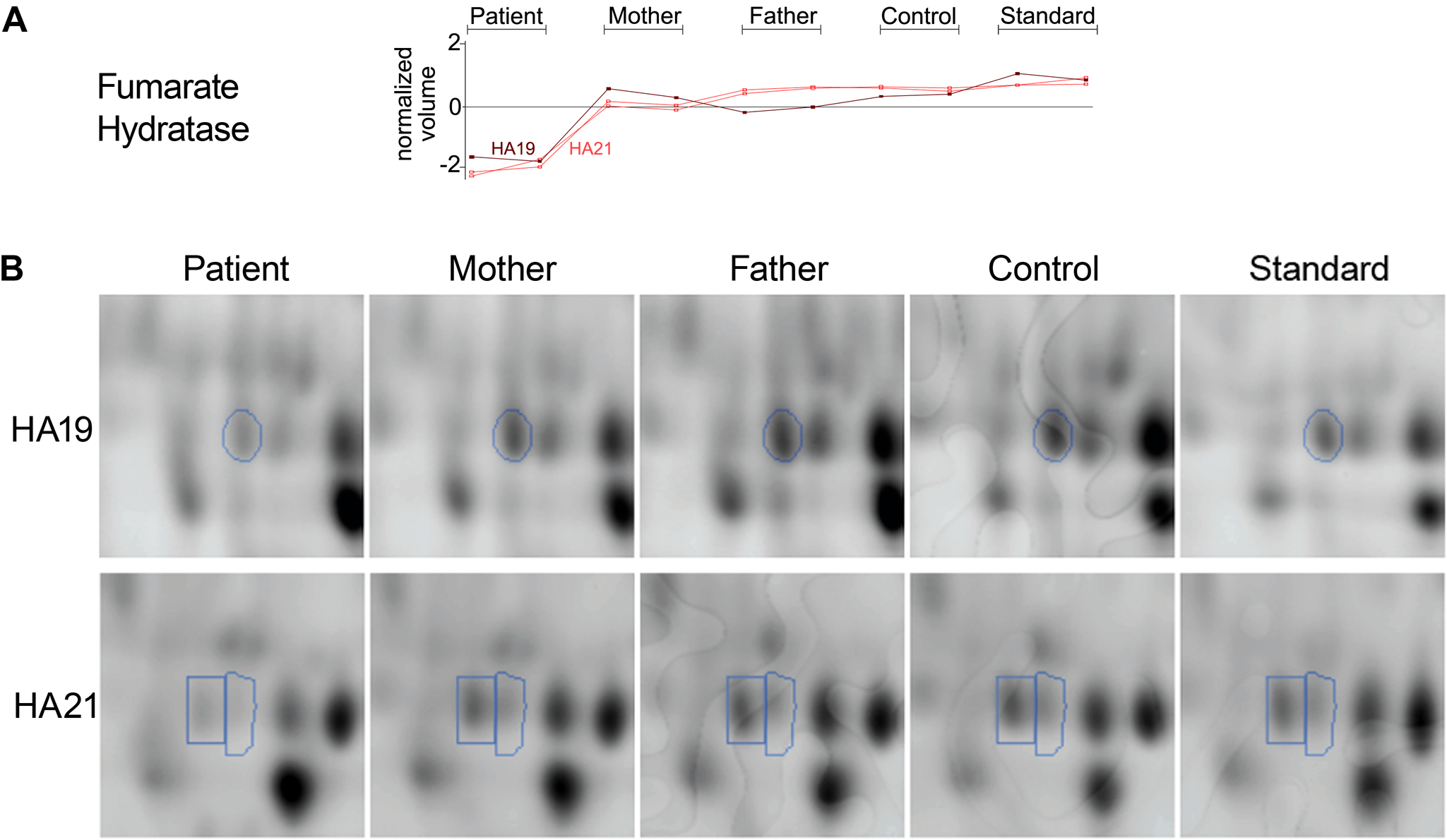

Supplement: Figure S4 — Fumarate Hydratase expression in HA19 and HA21. Included are all spots in which Fumarate Hydratase (FH) was identified as the predominant protein. A: Graph shows expression levels in normalized volume for both replicates run per sample. B: Images show sections of the gels where FH containing spots were excised (blue lines enclose spot areas; upper panel: HA19, middle panel: HA21). Pictures were exported from Same Spots and merged to show all relevant spots in one image per sample. (TIF) [file pone.0034237.s004.tif]
