# Supplementary material for: Assessment of the Red Cell Proteome of Young Patients with Unexplained Hemolytic Anemia by Two-Dimensional Differential In-Gel Electrophoresis (DIGE)
Source: PLoS One. 2012 Apr 3;7(4):e34237. doi: 10.1371/journal.pone.0034237 (PMC3317954; doi:10.1371/journal.pone.0034237)
Supplement: Table S1 — Complete Summary of Enzyme Assays performed on samples included in this study. (DOCX) [file pone.0034237.s005.docx]

|  |  | HA09 | | | HA19 | | | | HA21 | | | | HA24 | | | | |
| --- | --- | --- | --- | --- | --- | --- | --- | --- | --- | --- | --- | --- | --- | --- | --- | --- | --- |
| (IU / g Hb) | Normal range | P | M | C | P | M | F | C | P | M | F | C | P | M | F | S | C |
| Pyruvate Kinase | 11.1- 18.9 | 13.0 | 12.2 | 18.6 | 23.2 |  | 10 | 18.5 | 11.8 | 11.2 | 7.9 | 8.9 | 5.3 | 6.9 | 7.8 | 14.3 | 20.4 |
| Hexokinase | 1.02- 2.54 | 1.62 | 0.93 | 1.72 | 5.17 |  | 0.97 | 3.95 | 1.9 |  |  | 1.72 | 3.45 | 2.21 | 1.12 | 1.41 | 1.03 |
| G6PD | 7.9 - 16.3 | 9.8 | 11.0 | 11.0 | 25.9 |  | 9.94 | 17.7 | 14.9 |  |  |  |  |  |  |  |  |
| GPI | 38.8- 82.2 | 49 | 42.1 | 40.5 | 86.3 |  | 58.6 | 72.8 | 60.6 |  |  |  |  |  |  |  |  |
| Low system | 41- 51 % |  |  |  | 52.1 |  | 52.3 | 49.8 |  |  |  |  |  |  |  |  |  |
| TPI | 1317- 2905 |  |  |  | 1602 |  | 1240 | 1348 | 1387 |  |  |  |  |  |  |  |  |
| Glutathione Peroxidase | 21.3- 40.3 | 30.2 | 35.2 | 49.6 |  |  |  |  | 44 | 43.2 | 22.3 | 18 |  |  |  |  |  |
| Reduced Glutathione | 4.5 - 8.7 | 6.14 | 6.93 | 7.09 | 7.78 |  | 6.11 | 5.49 |  |  |  |  | 8.19 | 6.66 | 3.17 | 4.94 | 6.28 |
| Reticulocyes (%) | m 0.92-2.71 | 3.45 | 1.27 | 1.06 | 11.6 | 4.2 | 2.2 | 3.6 | 18.4 | 1.65 | 1.14 | 1.06 | 6.21 | 2.5 | 4.73 | 4.28 | 2.12 |
|  | f 0.61-2.20 |  |  |  |  |  |  |  |  |  |  |  |  |  |  |  |  |

Table S1: Complete Summary of Enzyme Assays performed on samples included in this study

P: Patient sample M: Mother F: Father S: Sister C: Control

m: male f:female

UI: International Unit Hb: Hemoglobin

G6PD: Glucose -6- Phosphate Dehydrogenase

GPI: Glucose Phosphate Isomerase

TPI: Triose Phosphate Isomerase
