# Supplementary material for: Assessment of the Red Cell Proteome of Young Patients with Unexplained Hemolytic Anemia by Two-Dimensional Differential In-Gel Electrophoresis (DIGE)
Source: PLoS One. 2012 Apr 3;7(4):e34237. doi: 10.1371/journal.pone.0034237 (PMC3317954; doi:10.1371/journal.pone.0034237)
Supplement: Table S2 — Complete list of all Proteins identified. (DOCX) [file pone.0034237.s006.docx]

# Table S2: Complete list of all Proteins identified

| Gene | Protein | Spectral count | Total of hits |
| --- | --- | --- | --- |
| ACOT7 | Isoform 1 of Cytosolic acyl coenzyme A thioester hydrolase | 2 | 1 |
|  | (41.7kD, pI 8.5) [IPI00010415](http://srs.ebi.ac.uk/srsbin/cgi-bin/wgetz?-e+[IPI-acc:IPI00010415]+-vn+2) |  |  |
| *ACTB* | Actin, cytoplasmic 2 (45kD, pI 6.2) [IPI00003269](http://srs.ebi.ac.uk/srsbin/cgi-bin/wgetz?-e+[IPI-acc:IPI00003269]+-vn+2) | 115 | 3 |
| *ACTBL2* | Actin, beta-like 2, DKFZp686D0972 hypothetical protein LOC345651 (42kD, pI 5.6) | 19 | 1 |
| *Actin* | Actin (42kD, pI 5.4) no unique peptides: ACTA2 or ACTG2 or ACTC1 or ACTA1 | 23 | 1 |
| ACTR1A | Alpha-centractin (42.6kD, pI 6.6) [IPI00029468](http://srs.ebi.ac.uk/srsbin/cgi-bin/wgetz?-e+[IPI-acc:IPI00029468]+-vn+2) | 72 | 5 |
| *ADSL* | Isoform 1 of Adenylosuccinate lyase (55kD, pI 7.1) [IPI00220887](http://srs.ebi.ac.uk/srsbin/cgi-bin/wgetz?-e+[IPI-acc:IPI00220887]+-vn+2) | 61 | 3 |
| AHSA1 | Activator of 90kDa heat shock protein ATPase homolog 1 | 14 | 2 |
|  | (38.2kD, pI 5.5) [IPI00030706](http://srs.ebi.ac.uk/srsbin/cgi-bin/wgetz?-e+[IPI-acc:IPI00030706]+-vn+2) |  |  |
| [*AIDA*](http://www.proteinatlas.org/ENSG00000186063) | Isoform 1 of axin interactor, dorsalisation –associated protein | *17* | *3* |
|  | (35kD, pI 6.6) [IPI00303602](http://srs.ebi.ac.uk/srsbin/cgi-bin/wgetz?-e+[IPI-acc:IPI00303602]+-vn+2) |  |  |
| AKR7A2 | Aflatoxin B1 aldehyde reductase member 2 (39.5kD, pI 7.2) [IPI00305978](http://srs.ebi.ac.uk/srsbin/cgi-bin/wgetz?-e+[IPI-acc:IPI00305978]+-vn+2) | 3 | 1 |
| *ALAD* | Delta-aminolevulinic acid dehydratase isoform a (39kD, pI 7.6) [IPI00790373](http://srs.ebi.ac.uk/srsbin/cgi-bin/wgetz?-e+[IPI-acc:IPI00790373]+-vn+2) | 385 | 17 |
| *ALB* | Serum Albumin (71.5kD, pI 6.7) [IPI00022434](http://srs.ebi.ac.uk/srsbin/cgi-bin/wgetz?-e+[IPI-acc:IPI00022434]+-vn+2) | 82 | 4 |
| *ALDH1A1* | Retinal dehydrogenase 1 (54.8kD, pI 6.7) [IPI00218914](http://srs.ebi.ac.uk/srsbin/cgi-bin/wgetz?-e+[IPI-acc:IPI00218914]+-vn+2) | 9 | 3 |
| *AP2M1* | AP-2 complex subunit mu-1 (59.6kD, pI 9.5) [IPI00022256](http://srs.ebi.ac.uk/srsbin/cgi-bin/wgetz?-e+[IPI-acc:IPI00022256]+-vn+2) | 3 | 1 |
| *APEH* | Acylamino-acid-releasing enzyme (81.2kD, pI 5.5) [IPI00337741](http://srs.ebi.ac.uk/srsbin/cgi-bin/wgetz?-e+[IPI-acc:IPI00337741]+-vn+2) | 43 | 2 |
| *APOA1BP* | Isoform 1 of Apolipoprotein A-I-binding protein precursor | 53 | 5 |
|  | (31.6kD, pI 7.7) [IPI00941988](http://srs.ebi.ac.uk/srsbin/cgi-bin/wgetz?-e+[IPI-acc:IPI00941988]+-vn+2) |  |  |
| ARCN1 | Archain 1 (61.6kD, pI 5.8) coatomer protein delta-COP [IPI00915422](http://srs.ebi.ac.uk/srsbin/cgi-bin/wgetz?-e+[IPI-acc:IPI00915422]+-vn+2) IPI00514053 | 3 | 1 |
| ARHGAP1 | Rho GTPase-activating protein 1 (50.4kD, 6.3) [IPI00020567](http://srs.ebi.ac.uk/srsbin/cgi-bin/wgetz?-e+[IPI-acc:IPI00020567]+-vn+2) | 11 | 2 |
| *ASNA1* | ATPase ASNA1 (40.2kD, pI 5.1) [IPI00013466](http://srs.ebi.ac.uk/srsbin/cgi-bin/wgetz?-e+[IPI-acc:IPI00013466]+-vn+2) IPI00783136 | 79 | 5 |
| ASPSCR1 | Isoform 2 of Tether containing UBX domain for GLUT4 | 2 | 1 |
|  | (69.9kD, pI 8.1) [IPI00065276](http://srs.ebi.ac.uk/srsbin/cgi-bin/wgetz?-e+[IPI-acc:IPI00065276]+-vn+2) IPI00065276 |  |  |
| ATG3 | ATG3 autophagy related 3 homolog (35.8kD, pI 4.7) [IPI00022254](http://srs.ebi.ac.uk/srsbin/cgi-bin/wgetz?-e+[IPI-acc:IPI00022254]+-vn+2) | 16 | 3 |
| *ATIC* | Bifunctional purine biosynthesis protein PURH (64.6kD, pI 6.7) [IPI00289499](http://srs.ebi.ac.uk/srsbin/cgi-bin/wgetz?-e+[IPI-acc:IPI00289499]+-vn+2) | 6 | 1 |
| ATP6V1E1 | Vacuolar ATP synthase subunit E 1 (26.1kD, pI 8.0) [IPI00003856](http://srs.ebi.ac.uk/srsbin/cgi-bin/wgetz?-e+[IPI-acc:IPI00003856]+-vn+2) | 22 | 2 |
| BAG1 | Isoform 1 of BAG family molecular chaperone regulator 1 | 3 | 1 |
|  | (38.8kD, pI 8.1) [IPI00218546](http://srs.ebi.ac.uk/srsbin/cgi-bin/wgetz?-e+[IPI-acc:IPI00218546]+-vn+2) IPI00300531 |  |  |
| BLMH | Bleomycin hydrolase (52.5kD, pI 6.3) [IPI00219575](http://srs.ebi.ac.uk/srsbin/cgi-bin/wgetz?-e+[IPI-acc:IPI00219575]+-vn+2) | 109 | 7 |
| *BPGM* | Bisphosphoglycerate mutase (30kD, pI 6.5) [IPI00215979](http://srs.ebi.ac.uk/srsbin/cgi-bin/wgetz?-e+[IPI-acc:IPI00215979]+-vn+2) | 52 | 5 |
| BTF3L4 | Transcription factor BTF3 homolog 4 (17.2kD, pI 6.4) [IPI00412792](http://srs.ebi.ac.uk/srsbin/cgi-bin/wgetz?-e+[IPI-acc:IPI00412792]+-vn+2) | 3 | 1 |
| C1orf123 | UPF0587 protein C1orf123 (18kD, pI 5.0) [IPI00016605](http://srs.ebi.ac.uk/srsbin/cgi-bin/wgetz?-e+[IPI-acc:IPI00016605]+-vn+2) | 10 | 2 |
| CA1 | Carbonic anhydrase 1 (28.8kD, pI 7.1) [IPI00215983](http://srs.ebi.ac.uk/srsbin/cgi-bin/wgetz?-e+[IPI-acc:IPI00215983]+-vn+2) | 12 | 1 |
| CAP1 | Adenylyl cyclase-associated protein 1 (51.9kD, pI 8.1) [IPI00008274](http://srs.ebi.ac.uk/srsbin/cgi-bin/wgetz?-e+[IPI-acc:IPI00008274]+-vn+2) | 23 | 2 |
| *CAPN1* | Calpain-1 catalytic subunit (81.8k, pI 5.7) [IPI00011285](http://srs.ebi.ac.uk/srsbin/cgi-bin/wgetz?-e+[IPI-acc:IPI00011285]+-vn+2) | 3 | 1 |
| *CAPZB* | Capping protein (31.3kD, pI 6.4) [IPI00026185](http://srs.ebi.ac.uk/srsbin/cgi-bin/wgetz?-e+[IPI-acc:IPI00026185]+-vn+2) | 61 | 4 |
| CASP8 | Isoform 9 of Caspase-8 precursor (61.8kD, pI 5.3) [IPI00220726](http://srs.ebi.ac.uk/srsbin/cgi-bin/wgetz?-e+[IPI-acc:IPI00220726]+-vn+2) IPI00000149 | 4 | 1 |
| CAST | Calpastatin isoform a (84.9kD, pI 5.4) [IPI00761160](http://srs.ebi.ac.uk/srsbin/cgi-bin/wgetz?-e+[IPI-acc:IPI00761160]+-vn+2) IPI00220857 | 10 | 1 |
| *CCS* | Copper chaperone for superoxide dismutase (29kD, pI 5.6) [IPI00021389](http://srs.ebi.ac.uk/srsbin/cgi-bin/wgetz?-e+[IPI-acc:IPI00021389]+-vn+2) | 2 | 1 |
| *CCT2* | T-complex protein 1 subunit beta (57.5kD, pI 6.4) [IPI00297779](http://srs.ebi.ac.uk/srsbin/cgi-bin/wgetz?-e+[IPI-acc:IPI00297779]+-vn+2) | 257 | 7 |
| CCT3 | T-complex protein 1 subunit gamma (60.5kD, pI 6.5) [IPI00553185](http://srs.ebi.ac.uk/srsbin/cgi-bin/wgetz?-e+[IPI-acc:IPI00553185]+-vn+2) IPI00290770 | 119 | 4 |
| *CCT4* | T-complex protein 1 subunit delta (58kD, pI 7.8) [IPI00302927](http://srs.ebi.ac.uk/srsbin/cgi-bin/wgetz?-e+[IPI-acc:IPI00302927]+-vn+2) | 190 | 7 |
| CCT5 | T-complex protein 1 subunit epsilon (59.6kD, pI 5.6) [IPI00010720](http://srs.ebi.ac.uk/srsbin/cgi-bin/wgetz?-e+[IPI-acc:IPI00010720]+-vn+2) | 27 | 3 |
| *CCT6A* | T-complex protein 1 subunit zeta (58kD, pI 6.7) [IPI00027626](http://srs.ebi.ac.uk/srsbin/cgi-bin/wgetz?-e+[IPI-acc:IPI00027626]+-vn+2) | 69 | 6 |
| *CCT6B* | T-complex protein 1 subunit zeta-2 (57.7kD, pI 7.1) [IPI00220656](http://srs.ebi.ac.uk/srsbin/cgi-bin/wgetz?-e+[IPI-acc:IPI00220656]+-vn+2) | 9 | 1 |
| *CCT7* | T-complex protein 1 subunit eta (59.3kD, pI 7.6) [IPI00018465](http://srs.ebi.ac.uk/srsbin/cgi-bin/wgetz?-e+[IPI-acc:IPI00018465]+-vn+2) | 212 | 8 |
| CCT8 | T-complex protein 1 subunit theta (59.8kD, pI 5.6) [IPI00794673](http://srs.ebi.ac.uk/srsbin/cgi-bin/wgetz?-e+[IPI-acc:IPI00794673]+-vn+2) IPI00784090 | 226 | 9 |
| *CLIC1* | Chloride intracellular channel protein 1 (29.9kD, pI 5.2) [IPI00010896](http://srs.ebi.ac.uk/srsbin/cgi-bin/wgetz?-e+[IPI-acc:IPI00010896]+-vn+2) | 72 | 3 |
| *CNDP2* | CNDP dipeptidase 2 (52.9kD, pI 6.0) [IPI00177728](http://srs.ebi.ac.uk/srsbin/cgi-bin/wgetz?-e+[IPI-acc:IPI00177728]+-vn+2) | 19 | 1 |
| *COASY* | Bifunctional coenzyme A synthase (62.3kD, pI 7.0) [IPI00184821](http://srs.ebi.ac.uk/srsbin/cgi-bin/wgetz?-e+[IPI-acc:IPI00184821]+-vn+2) | 4 | 1 |
| *COL3A1* | Isoform 1 of Collagen alpha-1(III) chain precursor (136kD, pI 6.6) [IPI00021033](http://srs.ebi.ac.uk/srsbin/cgi-bin/wgetz?-e+[IPI-acc:IPI00021033]+-vn+2) | 2 | 1 |
| COPS5 | COP9 signalosome complex subunit 5 (37.6kD, 6.5) [IPI00009958](http://srs.ebi.ac.uk/srsbin/cgi-bin/wgetz?-e+[IPI-acc:IPI00009958]+-vn+2) | 26 | 2 |
| COPS7A | COP9 signalosome complex subunit 7a (30.2kD, pI 8.2) [IPI00301419](http://srs.ebi.ac.uk/srsbin/cgi-bin/wgetz?-e+[IPI-acc:IPI00301419]+-vn+2) | 4 | 1 |
| CRKL | Crk-like protein (33.7kD, pI 6.7) IPI00004839 | 2 | 1 |
| CRLF3 | Cytokine receptor-like factor 3 (49.7kD, pI 5.1) [IPI00295387](http://srs.ebi.ac.uk/srsbin/cgi-bin/wgetz?-e+[IPI-acc:IPI00295387]+-vn+2) | 4 | 1 |
| CSNK2A1 | Casein kinase 2, alpha 1 polypeptide (45.9kD, pI 7.9) [IPI00744507](http://srs.ebi.ac.uk/srsbin/cgi-bin/wgetz?-e+[IPI-acc:IPI00744507]+-vn+2) IPI00016613 | 26 | 2 |
| *CTPS* | CTP synthase 1 (66.7kD, pI 6.5) [IPI00290142](http://srs.ebi.ac.uk/srsbin/cgi-bin/wgetz?-e+[IPI-acc:IPI00290142]+-vn+2) IPI00645702 | 15 | 1 |
| CUL4A | Cullin-4A (88kD, pI 8.1) [IPI00419273](http://srs.ebi.ac.uk/srsbin/cgi-bin/wgetz?-e+[IPI-acc:IPI00419273]+-vn+2) | 10 | 1 |
| *CUL5* | Cullin-5 (96.7kD, pI 8.4) [IPI00216003](http://srs.ebi.ac.uk/srsbin/cgi-bin/wgetz?-e+[IPI-acc:IPI00216003]+-vn+2) | 10 | 1 |
| *DARS* | Aspartyl-tRNA synthetase, cytoplasmic (57.1kD, pI 6.5) [IPI00216951](http://srs.ebi.ac.uk/srsbin/cgi-bin/wgetz?-e+[IPI-acc:IPI00216951]+-vn+2) | 18 | 5 |
| DCAF11 | DDB1 and CUL4 associated factor 11 (61.6kD, pI 6.4) [IPI00178972](http://srs.ebi.ac.uk/srsbin/cgi-bin/wgetz?-e+[IPI-acc:IPI00178972]+-vn+2) | 2 | 1 |
| DHRS11 | Isoform 1 of Dehydrogenase/reductase SDR family member 11 precursor | 15 | 1 |
|  | (28.3kD, 6.6) IPI00034280 |  |  |
| EEF1G | Elongation factor 1-gamma (50.1kD, pI 6.7) [IPI00937615](http://srs.ebi.ac.uk/srsbin/cgi-bin/wgetz?-e+[IPI-acc:IPI00937615]+-vn+2) IPI00000875 | 4 | 1 |
| EEF2 | Elongation factor 2 (95kD, pI 6.8) [IPI00186290](http://srs.ebi.ac.uk/srsbin/cgi-bin/wgetz?-e+[IPI-acc:IPI00186290]+-vn+2) | 20 | 3 |
| *EIF2S1* | Eukaryotic translation initiation factor 2 subunit 1 (36.1kD, pI 5.1) [IPI00219678](http://srs.ebi.ac.uk/srsbin/cgi-bin/wgetz?-e+[IPI-acc:IPI00219678]+-vn+2) | 35 | 1 |
| EIF2S3 | Eukaryotic translation initiation factor 2 subunit 3 (51.1kD, pI 8.4) [IPI00297982](http://srs.ebi.ac.uk/srsbin/cgi-bin/wgetz?-e+[IPI-acc:IPI00297982]+-vn+2) | 26 | 1 |
| EIF5 | Eukaryotic translation initiation factor 5 (49.2kD, pI 5.6) [IPI00022648](http://srs.ebi.ac.uk/srsbin/cgi-bin/wgetz?-e+[IPI-acc:IPI00022648]+-vn+2) | 18 | 2 |
| *EIF5A* | Isoform 2 of Eukaryotic translation initiation factor 5A-1 | 17 | 3 |
|  | (20.1kD, pI 7.0) [IPI00376005](http://srs.ebi.ac.uk/srsbin/cgi-bin/wgetz?-e+[IPI-acc:IPI00376005]+-vn+2) |  |  |
| FBXO7 | F-box only protein 7 (58.2kD, pI 6.8) [IPI00294567](http://srs.ebi.ac.uk/srsbin/cgi-bin/wgetz?-e+[IPI-acc:IPI00294567]+-vn+2) | 71 | 8 |
| *FDPS* | Farnesyl diphosphate synthase (48.2kD, pI 6.2) [IPI00914566](http://srs.ebi.ac.uk/srsbin/cgi-bin/wgetz?-e+[IPI-acc:IPI00914566]+-vn+2) IPI00101405 | 25 | 2 |
| FGD1 | FYVE, RhoGEF and PH domain-containing protein 1 (106kD, pI 6.6) [IPI00024312](http://srs.ebi.ac.uk/srsbin/cgi-bin/wgetz?-e+[IPI-acc:IPI00024312]+-vn+2) | 2 | 1 |
| *FH* | Fumarate hydratase, mitochondrial (54.6kD, pI 8.8) [IPI00296053](http://srs.ebi.ac.uk/srsbin/cgi-bin/wgetz?-e+[IPI-acc:IPI00296053]+-vn+2) | 49 | 4 |
| FLAD1 | Isoform 1 of FAD synthetase (65kD, pI 6.9) [IPI00220299](http://srs.ebi.ac.uk/srsbin/cgi-bin/wgetz?-e+[IPI-acc:IPI00220299]+-vn+2) | 2 | 1 |
| *G6PD* | Isoform Long of Glucose-6-phosphate 1-dehydrogenase | 169 | 6 |
|  | (63.8kD, pI 6.9) [IPI00216008](http://srs.ebi.ac.uk/srsbin/cgi-bin/wgetz?-e+[IPI-acc:IPI00216008]+-vn+2) |  |  |
| *GCLC* | Glutamate--cysteine ligase catalytic subunit (72.7kD, pI 6.1) [IPI00215768](http://srs.ebi.ac.uk/srsbin/cgi-bin/wgetz?-e+[IPI-acc:IPI00215768]+-vn+2) | 87 | 5 |
| *GCLM* | Glutamate--cysteine ligase regulatory subunit (30.7kD, pI 6.0) [IPI00010090](http://srs.ebi.ac.uk/srsbin/cgi-bin/wgetz?-e+[IPI-acc:IPI00010090]+-vn+2) | 5 | 1 |
| *GDI1* | Rab GDP dissociation inhibitor alpha (50.5kD, pI 5.1) [IPI00010154](http://srs.ebi.ac.uk/srsbin/cgi-bin/wgetz?-e+[IPI-acc:IPI00010154]+-vn+2) | 11 | 2 |
| *GDI2* | Rab GDP dissociation inhibitor beta (50.6kD, pI 6.5) [IPI00940148](http://srs.ebi.ac.uk/srsbin/cgi-bin/wgetz?-e+[IPI-acc:IPI00940148]+-vn+2) IPI00031461 | 139 | 8 |
| GLRX3 | Glutaredoxin-3 (37kD, pI 5.4) [IPI00008552](http://srs.ebi.ac.uk/srsbin/cgi-bin/wgetz?-e+[IPI-acc:IPI00008552]+-vn+2) | 60 | 4 |
| *GMPR* | GMP reductase 1 (37.4kD, pI 7.1) [IPI00304803](http://srs.ebi.ac.uk/srsbin/cgi-bin/wgetz?-e+[IPI-acc:IPI00304803]+-vn+2) | 164 | 21 |
| *GMPR2* | GMPR2 protein (45kD, pI 8.3) [IPI00449197](http://srs.ebi.ac.uk/srsbin/cgi-bin/wgetz?-e+[IPI-acc:IPI00449197]+-vn+2) | 20 | 3 |
| *GPS1* | Isoform 3 of COP9 signalosome complex subunit 1 (60.4kD, pI 6.9) [IPI00156282](http://srs.ebi.ac.uk/srsbin/cgi-bin/wgetz?-e+[IPI-acc:IPI00156282]+-vn+2) | 6 | 2 |
| *GSN* | Isoform 1 of Gelsolin precursor (85.6kD, pI 6.3) [IPI00026314](http://srs.ebi.ac.uk/srsbin/cgi-bin/wgetz?-e+[IPI-acc:IPI00026314]+-vn+2) IPI00641047 | 11 | 1 |
| GSS | Glutathione synthetase (52.3kD, pI 5.9) [IPI00010706](http://srs.ebi.ac.uk/srsbin/cgi-bin/wgetz?-e+[IPI-acc:IPI00010706]+-vn+2) | 47 | 4 |
| *GSTM3* | Glutathione S-transferase mu 3 (brain) (26.5kD, pI 5.5) [IPI00246975](http://srs.ebi.ac.uk/srsbin/cgi-bin/wgetz?-e+[IPI-acc:IPI00246975]+-vn+2) | 41 | 3 |
| HARS | Histidyl-tRNA synthetase, cytoplasmic (57.4kD, pI 6.9) [IPI00021808](http://srs.ebi.ac.uk/srsbin/cgi-bin/wgetz?-e+[IPI-acc:IPI00021808]+-vn+2) | 23 | 1 |
| *HDHD2* | Haloacid dehalogenase-like hydrolase domain containing 2 | 15 | 2 |
|  | (29.6kD, pI 6.4) [IPI00783874](http://srs.ebi.ac.uk/srsbin/cgi-bin/wgetz?-e+[IPI-acc:IPI00783874]+-vn+2) |  |  |
| *HERC1* | Guanine nucleotide exchange factor p532 (532kD, pI 6.0) [IPI00022479](http://srs.ebi.ac.uk/srsbin/cgi-bin/wgetz?-e+[IPI-acc:IPI00022479]+-vn+2) | 4 | 2 |
| *HPRT1* | Hypoxanthine phosphoribosyltransferase 1 (24.6kD, pI 7.0) [IPI00218493](http://srs.ebi.ac.uk/srsbin/cgi-bin/wgetz?-e+[IPI-acc:IPI00218493]+-vn+2) | 19 | 2 |
| *HSP90AA1* | Heat shock protein HSP 90-alpha (98kD, pI 5.2) [IPI00382470](http://srs.ebi.ac.uk/srsbin/cgi-bin/wgetz?-e+[IPI-acc:IPI00382470]+-vn+2) | 42 | 3 |
| *HSP90AB1* | Heat shock protein HSP 90-beta (84.7kD, pI 5.4) IPI00414676 | 4 | 1 |
| *HSPA1B* | Heat shock 70kDa protein 1A/1B (70kD, pI 5.6) [IPI00304925](http://srs.ebi.ac.uk/srsbin/cgi-bin/wgetz?-e+[IPI-acc:IPI00304925]+-vn+2) | 102 | 2 |
| *HSPA1L* | Heat shock 70kDa protein 1 like (70.4kD, pI 6.0) [IPI00939442](http://srs.ebi.ac.uk/srsbin/cgi-bin/wgetz?-e+[IPI-acc:IPI00939442]+-vn+2) IPI00301277 | 8 | 1 |
| *HSPA8* | Isoform 1 of Heat shock cognate 71kDa protein (70.8kD, pI 5.5) [IPI00003865](http://srs.ebi.ac.uk/srsbin/cgi-bin/wgetz?-e+[IPI-acc:IPI00003865]+-vn+2) | 130 | 4 |
| HSPB1 | Heat shock protein beta-1 (22.7kD, pI 6.4) [IPI00025512](http://srs.ebi.ac.uk/srsbin/cgi-bin/wgetz?-e+[IPI-acc:IPI00025512]+-vn+2) | 9 | 1 |
| IDE | Insulin-degrading enzyme (118kD, pI 6.8) [IPI00220373](http://srs.ebi.ac.uk/srsbin/cgi-bin/wgetz?-e+[IPI-acc:IPI00220373]+-vn+2) | 9 | 1 |
| *KPNB1* | Importin subunit beta-1 (97kD, pI 4.8) [IPI00001639](http://srs.ebi.ac.uk/srsbin/cgi-bin/wgetz?-e+[IPI-acc:IPI00001639]+-vn+2) | 57 | 3 |
| *LCP1* | L-plastin, Plastin-2 (70.2kD, pI 5.3) [IPI00010471](http://srs.ebi.ac.uk/srsbin/cgi-bin/wgetz?-e+[IPI-acc:IPI00010471]+-vn+2) | 77 | 2 |
| *LDHA* | Isoform 1 of L-lactate dehydrogenase A chain (36.6kD, pI 8.3) IPI00217966 | 37 | 2 |
| *LDHB* | L-lactate dehydrogenase B chain (36.6kD, pI 6.1) IPI00219217 | 66 | 4 |
| LETM1 | Leucine zipper-EF-hand-containing transmem.protein 1 | 2 | 1 |
|  | mitochondrial precursor (83.3kD, pI 6.7) IPI00017592 |  |  |
| LOC440917 | Similar to 14-3-3 protein epsilon (29.6kD, pI 5.0) | 29 | 1 |
|  | from 14-3-3 alpha or sigma or beta, gamma, theta… |  |  |
| *LOC653781* | Similar to Prostate, ovary, testis expressed protein on chromosome 2 (117kD, pI 6.0) | 4 | 1 |
| MAPK1 | Mitogen-activated protein kinase 1 (41.4kD, pI 7.0) IPI00003479 | 4 | 1 |
| MAPRE1 | Microtubule-associated protein, RP/EB family, member 1 (29.9kD, pI 5.1) IPI00017596 | 6 | 1 |
| MAT2A | S-adenosylmethionine synthetase isoform type-2 (43.6kD, 6.5) IPI00010157 | 6 | 1 |
| *MGC3207 / MRI1* | Hypothetical protein LOC84245 isoform 1 (39.1kD, 6.3) | 5 | 1 |
|  | Methylthioribose-1-phosphate isomerase IPI00005948 |  |  |
| *NAE1* | NEDD8 activating enzyme E1 subunit 1 (60.5kD, pI 5.4) [IPI00018968](http://srs.ebi.ac.uk/srsbin/cgi-bin/wgetz?-e+[IPI-acc:IPI00018968]+-vn+2) | 4 | 1 |
| NAP1L4 | Nucleosome assembly protein 1-like 4 (43kD, pI 4.7) IPI00941463 IPI00017763 | 3 | 1 |
| NAPA | Alpha-soluble NSF attachment protein (33.2kD, pI 5.4) IPI00009253 | 109 | 5 |
| *NAPRT1* | Nicotinate phosphoribosyltransferase domain-cont.prot. 1 (60.2kD, pI 5.8) IPI00465085 | 13 | 3 |
| NIF3L1 | Isoform 1 of NIF3-like protein 1 (42kD, pI 6.7) IPI00604624 | 9 | 2 |
| NME1;NME2 | Nucleoside diphosphate kinase (32.6kD, pI 8.5) IPI00604590 | 15 | 1 |
| NMI | N-myc (and STAT) interactor (35kD, pI 5.3) IPI00012450 | 2 | 1 |
| *NSF* | Vesicle-fusing ATPase (82.5kD, pI 7.0) IPI00006451 | 15 | 1 |
| *OXSR1* | Serine/threonine-protein kinase OSR1 (58kD, pI 6.4) IPI00010080 | 39 | 5 |
| PA2G4 | Proliferation-associated protein 2G4 (43,6kD, pI 6.1) IPI00299000 | 51 | 5 |
| PAFAH1B1 | Isoform 1 of Platelet-activating factor acetylhydrolase IB subunit alpha | 18 | 2 |
|  | (46.6kD, pI 7.4) IPI00218728 |  |  |
| PAFAH1B2 | Platelet-activating factor acetylhydrolase IB subunit beta (25.6kD, pI 5.9) IPI00026546 | 3 | 1 |
| PDE12 | Phosphodiesterase (67kD, pI 6.6) IPI00174390 | 12 | 2 |
| PEPD | Xaa-Pro dipeptidase (peptidase D) (54.5kD, pI 6.0) IPI00257882 | 2 | 1 |
| PGAM1 | Phosphoglycerate mutase 1 (brain) (28.8kD, pI 7.2) IPI00549725 | 58 | 3 |
| PIP4K2A | Phosphatidylinositol-5-phosphate 4-kinase type-2 alpha (46kD, pI 7.0) IPI00009688 | 26 | 2 |
| PITHD1 | PITH domain-containing protein 1 (24kD, pI 5.8) [IPI00015351](http://srs.ebi.ac.uk/srsbin/cgi-bin/wgetz?-e+[IPI-acc:IPI00015351]+-vn+2) | 82 | 7 |
| *PNP* | Purine Nucleoside Phosphorylase (32.5kD, pI 7.2) IPI00017672 | 613 | 21 |
| *POTEI* | POTE ankyrin domain family, member I, (42kD, pI 6.3) [IPI00888712](http://srs.ebi.ac.uk/srsbin/cgi-bin/wgetz?-e+[IPI-acc:IPI00888712]+-vn+2) | 17 | 1 |
| PPME1 | Protein phosphatase methylesterase 1 (42.6kD, pI 5.8) IPI00007694 | 15 | 1 |
| *PPP2CA* | Serine/threonine-protein phosphatase 2A catalytic subunit alpha isoform | 3 | 1 |
|  | (35.5kD, pI 5.5) IPI00008380 |  |  |
| *PRDX1* | Peroxiredoxin-1 (22.1kD, pI 8.1) IPI00000874 | 23 | 4 |
| *PRDX2* | Peroxiredoxin-2 (21.8kD, pI 6.0) IPI00027350 | 371 | 9 |
| PRPSAP1 | Phosphoribosyl pyrophosphate synthetase-associated protein 1 | 5 | 1 |
|  | (42kD, pI 8.5) IPI00291578 |  |  |
| *PRPSAP2* | Phosphoribosyl pyrophosphate synthetase-associated protein 2 | 3 | 1 |
|  | (42.4kD, pI 8.5) IPI00003168 |  |  |
| PSMA1 | Isoform Long of Proteasome subunit alpha type-1 (30.2kD, pI 7) IPI00472442 | 39 | 3 |
| *PSMA2* | Proteasome subunit, alpha type- 2 (25.9kD, pI 7.4) IPI00219622 | 30 | 2 |
| PSMA3 | Isoform 1 of Proteasome subunit alpha type-3 (28.4kD, pI 5.3) IPI00419249 IPI00171199 | 23 | 1 |
| *PSMA4* | Proteasome subunit alpha type-4 (29.4kD, pI 7.7) IPI00299155 | 192 | 7 |
| *PSMA5* | Proteasome subunit alpha type-5 (26.4kD, pI 4.8) IPI00291922 | 3 | 1 |
| *PSMA6* | Proteasome subunit alpha type-6 (27.4kD, pI 6.7) IPI00029623 | 16 | 1 |
| *PSMA7* | Isoform 1 of Proteasome subunit alpha type-7 (27.8kD, pI 8.5) IPI00024175 | 66 | 6 |
| *PSMB1* | Proteasome subunit beta type-1 precursor (26.5kD, pI 8.1) IPI00025019 | 4 | 1 |
| *PSMB2* | Proteasome beta 2 subunit variant (Fragment) | 39 | 2 |
|  | (27.8kD, pI 9.3) IPI00844375 / IPI00028006 |  |  |
| PSMB3 | Proteasome subunit beta type-3 (22.9kD, pI 6.5) IPI00028004 | 31 | 2 |
| PSMB4 | Proteasome subunit beta type-4 precursor (29.2kD, pI 6.0) IPI00555956 | 22 | 3 |
| PSMB5 | Proteasome subunit, beta type, 5 (28kD, 6.5) IPI00479306 | 58 | 2 |
| PSMB7 | Proteasome subunit beta type-7 precursor (29.9kD, pI 7.7) IPI00003217 | 16 | 1 |
| *PSMC2* | 26S protease regulatory subunit 7 (48.5kD, pI 5.9) IPI00021435 | 14 | 3 |
| *PSMC5* | 26S protease regulatory subunit 8 (45.6kD, pI 7.5) IPI00023919 | 196 | 6 |
| PSMC6 | 26S protease regulatory subunit S10B (44.1kD, pI 7.5) IPI00926977 IPI00021926 | 59 | 3 |
| *PSMD2* | 26S proteasome non-ATPase regulatory subunit 2 (100kD, pI 5.2) IPI00012268 | 14 | 2 |
| *PSMD7* | 26S proteasome non-ATPase regulatory subunit 7 (37kD, pI 6.8) IPI00019927 | 94 | 7 |
| PSMD8 | Proteasome 26S non-ATPase subunit 8 (39.6kD, pI 9.7) IPI00937278 IPI00010201 | 54 | 5 |
| PSMD9 | Isoform p27-L of 26S proteasome non-ATPase regulatory subunit 9 | 24 | 4 |
|  | (24.6kD, pI 7.0) IPI00010860 |  |  |
| PSMD10 | 26S proteasome non-ATPase regulatory subunit 10 (24kD, pI 6.1) IPI00003565 | 8 | 1 |
| *PSMD11* | Proteasome 26S non-ATPase subunit 11 variant (47.5kD, pI 6.5) IPI00105598 | 87 | 3 |
| *PSMD12* | 26S proteasome non-ATPase regulatory subunit 12 (52.9kD, pI 7.6) IPI00185374 | 75 | 3 |
| PSMD14 | 26S proteasome non-ATPase regulatory subunit 14 (34.5kD, pI 6.5) IPI00024821 | 19 | 2 |
| PSME1 | Proteasome activator subunit 1 (PA28 alpha) (28.7kD, pI 5.7) IPI00479722 IPI00030154 | 49 | 3 |
| PSME2 | Proteasome activator subunit 2 (PA28 beta) (27.4kD, pI 5.7) IPI00943181 IPI00384051 | 24 | 3 |
| PSMG1 | Proteasome assembly chaperone 1 (32.8kD, pI 7.2) IPI00030770 | 2 | 1 |
| PURA | Transcriptional activator protein Pur-alpha (34.9kD, pI 6.4) IPI00023591 | 34 | 1 |
| QDPR | Dihydropteridine reductase (25.7kD, pI 7.4) IPI00014439 | 8 | 1 |
| *RAB10* | Ras-related protein Rab-10 (22.5kD, pI 8.4) IPI00016513 | 5 | 1 |
| RAD23A | UV excision repair protein RAD23 homolog A (39.6kD, pI 4.6) IPI00008219 | 3 | 1 |
| RANBP1 | Ran-specific GTPase-activating protein (23.3kD, pI 5.3) IPI00414127 | 12 | 1 |
| RHOA | Ras homolog gene family, member A (22.2kD, pI 7.1) IPI00478231 IPI00027500 | 16 | 2 |
| RHOC | Ras homolog gene family, member C (22kD, pI 7.6) IPI00027434 | 7 | 1 |
| RPSA | Ribosomal protein SA (33.3kD, pI 4.9) IPI00553164 IPI00413108 | 66 | 4 |
| *RRM1* | Ribonucleoside-diphosphate reductase large subunit (90kD, pI 7.1) IPI00013871 | 16 | 2 |
| RUVBL1 | RuvB-like 1 (50.2kD, pI 6.4) IPI00021187 | 2 | 1 |
| *RUVBL2* | RuvB-like 2 (51.1kD, pI 5.6) IPI00009104 | 2 | 1 |
| *SBNO1 or SBNO2* | Strawberry notch homolog 1 or 2 (154kD, pI 7.9 or 6.8) IPI00023649 / IPI00024900 | 2 | 1 |
| SCFD1 | Sec1 family domain containing protein 1 (72.3kD, pI 6.3) IPI00165261 | 16 | 3 |
| SH3GLB2 | Isoform 2 of SH3 domain GRB2-like endophilin B2 | 4 | 2 |
|  | (44.7kD, pI 5.6) IPI00398828 IPI00024540 |  |  |
| SKP1 | Isoform 1 of S-phase kinase-associated protein 1A (18.6kD, pI 4.5) IPI00301364 | 2 | 1 |
| SMS | Spermine synthase (41.2kD, pI 5.0) IPI00005102 | 6 | 1 |
| SNF8 | Isoform 1 of Vacuolar-sorting protein SNF8 (28.8kD, pI 6.8) IPI00101524 | 3 | 1 |
| *SOD1* | Superoxide dismutase 1, soluble (16.1kD, pI 6.3) IPI00218733 | 9 | 2 |
| *ST13* | Hsc70-interacting protein (41.3kD, pI 5.3) PI00032826 | 4 | 2 |
| STAT5B | Signal transducer and activator of transcription 5B (89.8kD, pI 6.1) IPI00103415 | 4 | 1 |
| *STIP1* | Stress-induced-phosphoprotein 1 (68kD, pI 7.7) IPI00013894 | 14 | 1 |
| SUGT1 | Isoform 1 of Suppressor of G2 allele of SKP1 homolog (41.2kD, pI 5.2) IPI00828150 | 7 | 2 |
| TARS | Threonyl-tRNA synthetase, cytoplasmic (83.4kD, pI 6.7) IPI00329633 | 2 | 1 |
| *TCP1* | T-complex protein 1 subunit alpha (60kD, pI 6.1) IPI00290566 | 91 | 4 |
| TET1 | Tet oncogene 1 (235kD, pI 8.3) IPI00303112 | 3 | 1 |
| *TGM2* | Isoform 1 of Protein-glutamine gamma-glutamyltransferase 2 | 22 | 4 |
|  | (77.3kD, pI 5.2) IPI00294578 |  |  |
| *TPP2* | Tripeptidyl peptidase II (140kD, pI 6.5) IPI00020416 | 3 | 1 |
| TPT1 | Tumor protein, translationally-controlled 1 (21.5kD, pI 5.0) IPI00009943 | 4 | 1 |
| TSN | Translin (26.1kD, pI 6.4) IPI00018768 | 32 | 2 |
| *TSTA3* | GDP-L-fucose synthetase (35.8kD, pI 6.6) IPI00014361 | 202 | 7 |
| TUBA1B | Tubulin alpha-1B chain (50.1kD, pI 5.1) IPI00930688 IPI00387144 | 56 | 1 |
| TUBA4A | Tubulin alpha-4A chain (49.9kD, pI 5.1) IPI00007750 | 41 | 1 |
| TUBB | Tubulin beta chain (49.6kD, pI 4.9) IPI00023598 | 104 | 1 |
| TUBB2B | Tubulin beta-2B chain (49.9kD, pI 4.9) IPI00031370 | 39 | 1 |
| TUBB2C | Tubulin beta-2C chain (49.8kD, pI 4.9) IPI00007752 | 61 | 2 |
| TUBB3 | Tubulin, beta-3 (88.4kD, pI 5.9) IPI00013683 | 32 | 1 |
| TUBB4 | Tubulin beta-4 chain (49.5kD, pI 4.9) IPI00023598 | 51 | 1 |
| TUBB6 | Tubulin, beta 6 (50kD, pI 4.9) IPI00646779 | 31 | 1 |
| TXNL1 | Thioredoxin-like 1 (36.7kD, pI 4.9) IPI00305692 | 70 | 2 |
| TXNRD1 | Thioredoxin reductase 1 isoform 3 (71.1kD, pI 5.5) IPI00783641 IPI00554786 | 33 | 5 |
| UBA1 | Ubiquitin-activating enzyme E1 (117kD, pI 5.8) IPI00645078 | 4 | 1 |
| *UROD* | Uroporphyrinogen decarboxylase (40.7kD, pI 6.1) IPI00301489 | 51 | 5 |
| *USP14* | Ubiquitin carboxyl-terminal hydrolase 14 (56kD, pI 5.3) IPI00219913 | 214 | 6 |
| *USP5* | Ubiquitin specific peptidase 5 (isopeptidase T) (95.7kD, pI 5.0) IPI00024664 | 3 | 1 |
| *VCP* | Valosin-containing protein, transitional endoplasmic reticulum ATPase | 113 | 3 |
|  | (89.3kD, pI 5.2) IPI00022774 |  |  |
| WARS | Tryptophanyl-tRNA synthetase, cytoplasmic (53kD, pI 6.2) IPI00295400 | 17 | 1 |
| WDR77 | Methylosome protein 50 (36.7kD, pI 5.2) IPI00647794 IPI00012202 | 2 | 1 |
| XPNPEP1 | Xaa-Pro aminopeptidase 1 (74.9kD, pI 5.9) IPI00793375 | 11 | 1 |
| *XPO7* | Exportin 7 isoform a (125kD, pI 6.3) IPI00302458 | 117 | 7 |
| *YWHAG* | 14-3-3 protein gamma (28.3kD, pI 4.9) IPI00220642 | 4 | 1 |
| YWHAH | 14-3-3 protein eta (28.2kD, pI 4.8) IPI00216319 | 4 | 1 |
| *YWHAZ* | 14-3-3 protein zeta/delta (27.7kD, pI 4.8) IPI00021263 | 7 | 1 |

Gene: HGNC Symbol for coding human gene

Protein: HGNC Symbol for protein identified

Spectral count: Sum of spectral counts for peptides unique to identified protein from all spots where this protein was found

Total of Hits: Number of spots where identified protein was found
