# Supplementary material for: Assessment of the Red Cell Proteome of Young Patients with Unexplained Hemolytic Anemia by Two-Dimensional Differential In-Gel Electrophoresis (DIGE)
Source: PLoS One. 2012 Apr 3;7(4):e34237. doi: 10.1371/journal.pone.0034237 (PMC3317954; doi:10.1371/journal.pone.0034237)
Supplement: Table S3 — Proteins not listed in Red Blood Cell protein databases. (DOCX) [file pone.0034237.s007.docx]

Table S3: Proteins not listed in Red Blood Cell protein databases: <http://141.61.102.16/rbc/>, <http://ebm.rsmjournals.com/cgi/content/full/232/11/1391#AFF3>, <http://proteomique.ipbs.fr/pairs/www/rbc_browser.htm>

| Gene | Protein | Expression level | Coverage% | SC | Hits |
| --- | --- | --- | --- | --- | --- |
| RANBP1 | Ran-specific GTPase-activating protein | N/A | 29.9 | 12 | 1 |
| TET1 | Tet oncogene 1 | N/A | 0.6 | 3 | 1 |
| *HARS* | Histidyl-tRNA synthetase, cytoplasmic | N/A | 25.9 | 23 | 1 |
| *HERC1* | Guanine nucleotide exchange factor p532 | N/A | 0.2 | 4 | 2 |
| TUBB | Tubulin beta chain | HA21 🡹 | 52 | 104 | 1 |
| TUBB4 | Tubulin beta-4 chain | HA21 🡹 | 49.5 | 51 | 1 |
| TUBA4A | Tubulin alpha-4A chain | HA21 🡹 | 46 | 41 | 1 |
| TUBB3 | Tubulin, beta- 3 | HA21 🡹 | 14.9 | 32 | 1 |
| TUBB6 | Tubulin, beta 6 | HA21 🡹 | 30.4 | 31 | 1 |
| RHOC | Ras homolog gene family, member C | N/A | 25.5 | 7 | 1 |
| *FGD1* | FYVE, RhoGEF and PH domain-containing protein 1 | N/A | 0.8 | 2 | 1 |
| *APOA1BP* | Isoform 1 of Apolipoprotein A-I-binding protein precursor | HA19 🡻 | 24.3 | 53 | 5 |
| *GSTM3* | Glutathione S-transferase mu 3 (brain) | N/A | 46.7 | 41 | 3 |
| PGAM1 | Phosphoglycerate mutase 1 | HA24 🡹 | 53.9 | 58 | 3 |
| *GMPR2* | GMPR2 protein | N/A | 24 | 20 | 3 |
| **POTEKP** | POTE ankyrin domain family, member K | N/A | 6.9 | 17 | 1 |
| *HDHD2* | Haloacid dehalogenase-like hydrolase domain containing | HA21 🡻 | 14.3 | 15 | 2 |
| PRPSAP1 | Phosphoribosyl pyrophosphate synthetase-associated protein 1 | N/A | 12.7 | 5 | 1 |
| *COL3A1* | Isoform 1 of Collagen alpha-1(III) chain precursor | HA21 🡹 | 1.2 | 2 | 1 |
| LETM1 | Leucine zipper-EF-hand-containing transmem.protein 1,  mitochondrial precursor | N/A | 3.5 | 2 | 1 |
| *SBNO1/2* | Strawberry notch homolog 1 or 2 | N/A | 0.8 | 2 | 1 |

Gene: HGNC Symbol for coding human gene Protein: HGNC Symbol for protein identified

Expression level: Expression measured (normalized volume) in patient sample compared to average of controls

N/A: Protein not predominant protein running in that spot,

🡹: Patient expression higher than controls, 🡻: Patient expression lower than controls

Spectral count: Sum of spectral counts for peptides unique to identified protein from all spots where this protein was found

Coverage: Percentage of aminoacid sequence that is covered by unique peptides to identify protein

Hits: Number of spots where identified protein was found
