# Supplementary material for: Assessment of the Red Cell Proteome of Young Patients with Unexplained Hemolytic Anemia by Two-Dimensional Differential In-Gel Electrophoresis (DIGE)
Source: PLoS One. 2012 Apr 3;7(4):e34237. doi: 10.1371/journal.pone.0034237 (PMC3317954; doi:10.1371/journal.pone.0034237)
Supplement: Table S4 — A: Differentially expressed spots with expression level in Patient sample higher compared to controls. B: Differentially expressed spots with expression level in Patient sample lower compared to controls. (DOCX) [file pone.0034237.s008.docx]

Table S4, A: Differentially expressed spots with expression level in Patient sample higher compared to controls

| ID | Fold change | Gene | Protein | SC |
| --- | --- | --- | --- | --- |
| HA09 |  |  |  |  |
|  | + 4.29 | HSPA1B | Heat shock 70kDa protein 1A/ | 52 |
|  |  | HSPA8 | 1B Isoform 1 of Heat shock cognate 71kDa | 10 |
|  | + 4.31 | HSP90AA1 | Heat shock protein HSP 90-alpha | 33 |
|  |  | HSP90AB1 | Heat shock protein HSP 90-beta | 4 |
|  | + 2.69 | HSPA8 | Isoform 1 of Heat shock cognate 71kDa | 56 |
|  | + 2.05 | HSPA1B | Heat shock 70kDa protein 1A/1B | 50 |
|  |  | HSPA8 | Isoform 1 of Heat shock cognate 71kDa | 23 |
|  |  | HSPA1L | Heat shock 70kDa protein 1 like | 8 |
|  | + 2.01 | ACTR1A | Alpha-centractin | 21 |
|  | + 4.5 | ACTB | Actin, cytoplasmic 2 | 21 |
| HA19 |  |  |  |  |
|  | + 5.3 | XPO7 | Exportin 7 isoform a | 7 |
|  | + 3.47 | ATG3 | ATG3 autophagy related 3 homolog | 10 |
|  | + 2.64 | RPSA | Ribosomal protein SA | 26 |
|  | + 2.41 | TXNL1 | Thioredoxin-like 1 | 11 |
|  | + 3.36 | WARS | Tryptophanyl-tRNA synthetase, cytoplasmic | 17 |
|  | + 3.56 | XPO7 | Exportin 7 isoform a | 8 |
|  | + 3.28 | XPO7 | Exportin 7 isoform a | 7 |
|  | + 2.69 | AHSA1 | Activator of 90kDa heat shock protein ATPase homolog 1 | 7 |
|  | + 2.36 | EIF2S1 | Eukaryotic translation initiation factor 2 subunit 1 | 35 |
|  | + 1.92 | CCT3 | T-complex protein 1 subunit gamma | 13 |
|  | + 2.25 | UROD | Uroporphyrinogen decarboxylase | 6 |
|  | + 1.88 | CCT4 | T-complex protein 1 subunit delta | 65 |
|  |  | CCT7 | T-complex protein 1 subunit eta | 9 |
|  | + 1.79 | TSTA3 | GDP-L-fucose synthetase | 45 |
|  | + 1.72 | TCP1 | T-complex protein 1 subunit alpha | 19 |
|  | + 1.75 | GLRX3 | Glutaredoxin-3 | 22 |
|  | + 1.72 | G6PD | Isoform Long of Glucose-6-phosphate 1-dehydrogenase | 54 |
|  | + 1.62 | GCLC | Glutamate-cysteine ligase catalytic subunit | 25 |
| HA21 |  |  |  |  |
|  | + 7.65 | EEF2 | Elongation factor 2 | 7 |
|  | + 6.31 | EEF2 | Elongation factor 2 | 9 |
|  | + 5.41 | RPSA | Ribosomal protein SA | 30 |
|  | + 5.58 | RRM1 | Ribonucleoside-diphosphate reductase large subunit | 5 |
|  | + 4.93 | FBXO7 | F-box only protein 7 | 25 |
|  | + 4.63 | RRM1 | Ribonucleoside-diphosphate reductase large subunit | 11 |
|  | + 3.57 | BTF3L4 | Transcription factor BTF3 homolog 4 | 3 |
|  | + 4.11 | ATG3 | ATG3 autophagy related 3 homolog | 3 |
|  | + 3.59 | TUBB | Tubulin beta chain | 104 |
|  |  | TUBB4 | Tubulin beta-4 chain | 51 |
|  |  | TUBB6 | Tubulin, beta-6 | 31 |
|  | + 2.91 | UROD | Uroporphyrinogen decarboxylase | 37 |
|  | + 2.47 | ACTR1A | Alpha-centractin | 39 |
|  | + 1.89 | CCT3 | T-complex protein 1 subunit gamma | 68 |
|  | + 2.33 | TXNL1 | Thioredoxin-like 1 | 59 |
|  | + 2.43 | ASNA1 | ATPase ASNA1 | 25 |
|  | + 2.63 | XPO7 | Exportin 7 isoform a | 3 |
|  | + 1.77 | CCT4 | T-complex protein 1 subunit delta | 66 |
|  |  | CCT7 | T-complex protein 1 subunit eta | 18 |
|  | + 1.88 | TCP1 | T-complex protein 1 subunit alpha | 50 |
|  | + 2.02 | KPNB1 | Importin subunit beta-1 | 49 |
|  | + 2.19 | PITHD1 | PITH domain-containing protein 1 | 19 |
|  | + 2.14 | TSTA3 | GDP-L-fucose synthetase | 25 |
|  | + 2.23 | TUBA1B | Tubulin alpha-1B chain | 56 |
|  |  | TUBA4A | Tubulin alpha-4A chain | 41 |
|  | + 2.13 | TSTA3 | GDP-L-fucose synthetase | 41 |
|  | + 1.66 | CCT2 | Chaperonin containing TCP1, subunit 2 | 86 |
|  | + 1.78 | COL3A1 | Isoform 1 of Collagen alpha-1(III) chain precursor | 2 |
|  | + 1.52 | PSMC5 | 26S protease regulatory subunit 8 | 63 |
|  | + 1.57 | XPO7 | Exportin 7 isoform a | 25 |
| HA24 |  |  |  |  |
|  | + 2.49 | ACTB | Actin, cytoplasmic 2 | 86 |
|  | + 2.04 | PNP | Purine Nucleoside Phosphorylase | 73 |

ID: Name of sample set (HA09, HA19, HA21, HA24)

Fold Change: Comparison of normalized volume in Patient sample with average of controls

Gene: HGNC Symbol for coding human gene

Protein: HGNC Symbol for protein identified

SC: Spectral count

Table S4, B: Differentially expressed spots with expression level in Patient sample lower compared to controls

| ID | Fold change | Gene | | Protein | SC |
| --- | --- | --- | --- | --- | --- |
| HA09 |  | |  |  |  |
|  | - 2.26 | | GCLC | Glutamate-cysteine ligase catalytic subunit | 2 |
|  | - 2.02 | | USB14 | Ubiquitin carboxyl-terminal hydrolase 14 | 8 |
|  | - 1.96 | | PPME1 | Protein phosphatase methylesterase 1 | 15 |
|  | - 2.00 | | CCT3 | T-complex protein 1 subunit gamma | 6 |
|  | - 2.08 | | CCT8 | T-complex protein 1 subunit theta | 16 |
|  |  |  | CCT5 | T-complex protein 1 subunit epsilon | 12 |
|  | - 2.05 | | PSMC5 | 26S protease regulatory subunit 8 | 37 |
|  | - 2.03 | | G6PD | Glucose-6-phosphate 1-dehydrogenase | 48 |
|  | - 1.87 | | CCT6A | T-complex protein 1 subunit zeta | 2 |
|  | - 1.74 | | CCT7 | T-complex protein 1 subunit eta | 29 |
|  |  |  | CCT4 | T-complex protein 1 subunit delta | 14 |
|  |  |  | CCT2 | T-complex protein 1 subunit beta | 7 |
|  | - 1.96 | | CCT2 | Chaperonin containing TCP1, subunit 2 | 40 |
|  | - 1.62 | | CCT3 | T-complex protein 1 subunit gamma | 32 |
|  |  |  | CCT6A | T-complex protein 1 subunit zeta | 4 |
|  | - 1.62 | | PITHD1 | PITH domain-containing protein 1 | 18 |
|  | - 1.68 | | GLRX3 | Glutaredoxin-3 | 8 |
|  | - 1.69 | | CCT5 | T-complex protein 1 subunit epsilon | 8 |
|  | - 1.58 | | ALAD | Delta-aminolevulinic acid dehydratase isoform a | 10 |
|  | - 1.58 | | PSMB7 | Proteasome subunit beta type-7 precursor | 16 |
|  | - 1.55 | | PSMA1 | Isoform Long Proteasome subunit alpha type-1 | 12 |
|  | - 1.55 | | PSMC6 | 26S protease regulatory subunit S10B | 39 |
| HA19 |  | |  |  |  |
|  | - 7.7 | | PNP | Purine Nucleoside Phosphorylase | 23 |
|  | - 5.83 | | PNP | Purine Nucleoside Phosphorylase | 6 |
|  | - 3.09 | | PNP | Purine Nucleoside Phosphorylase | 59 |
|  | - 2.82 | | CLIC1 | Chloride intracellular channel protein 1 | 20 |
|  | - 3.09 | | RHOA | Ras homolog gene family, member A | 3 |
|  | - 2.65 | | GDI2 | Rab GDP dissociation inhibitor beta | 11 |
|  | - 2.8 | | LCP1 | L-plastin, Plastin-2 | 24 |
|  | - 2.44 | | PSMA4 | Proteasome subunit alpha type-4 | 27 |
|  |  | | PSMD9 | Proteasome 26S subunit, non-ATPase, 9 | 10 |
|  | - 2.26 | | FH | Fumarate hydratase, mitochondrial | 16 |
|  | - 2.49 | | USB14 | Ubiquitin carboxyl-terminal hydrolase 14 | 19 |
|  | - 2.17 | | PSMA1 | Isoform Long Proteasome subunit alpha type-1 | 9 |
|  | - 2.09 | | PSMA4 | Proteasome subunit alpha type-4 | 8 |
|  | - 1.84 | | CAPZB | Capping protein | 24 |
|  | - 1.72 | | PSMB5 | Proteasome subunit, beta type, 5 | 52 |
|  | - 1.8 | | APOA1BP | Isoform 1 Apolipoprotein A-I-binding protein | 35 |
|  | - 1.79 | | C1orf123 | UPF0587 protein C1orf123 | 6 |
|  | - 1.51 | | PSMB3 | Proteasome subunit beta type-3 | 22 |
|  | - 1.5 | | PSMB1 | Proteasome subunit beta type-1 precursor | 4 |
| HA21 |  | |  |  |  |
|  | - 9.07 | | PNP | Purine Nucleoside Phosphorylase | 37 |
|  | - 5.19 | | PNP | Purine Nucleoside Phosphorylase | 40 |
|  | - 4.48 | | PNP | Purine Nucleoside Phosphorylase | 48 |
|  | - 3.75 | | GDI1 | Rab GDP dissociation inhibitor alpha | 47 |
|  |  | | GDI2 | Rab GDP dissociation inhibitor beta | 7 |
|  | - 3.27 | | ALAD | Delta-aminolevulinic acid dehydratase isoform a | 23 |
|  | - 2.44 | | PSMB4 | Proteasome subunit beta type-4 precursor | 14 |
|  | - 2.93 | | FH | Fumarate hydratase, mitochondrial | 12 |
|  | - 2.71 | | SOD1 | Superoxide dismutase 1, soluble | 4 |
|  | - 2.43 | | GDI2 | Rab GDP dissociation inhibitor beta | 50 |
|  | - 2.32 | | TSN | Translin | 20 |
|  | - 2.39 | | CAPZB | Capping protein | 12 |
|  | - 2.16 | | ALAD | Delta-aminolevulinic acid dehydratase isoform a | 30 |
|  | - 2.32 | | CUL4A | Cullin-4A | 10 |
|  |  | | CUL5 | Cullin-5 | 10 |
|  | - 2.16 | | LCP1 | L-plastin, Plastin-2 | 53 |
|  | - 2.20 | | PSMA4 | Proteasome subunit alpha type-4 | 58 |
|  | - 2.16 | | FH | Fumarate hydratase, mitochondrial | 8 |
|  | - 2.22 | | GCLC | Glutamate--cysteine ligase catalytic SU | 43 |
|  | - 2.03 | | PSMA7 | Isoform 1 of Proteasome subunit alpha type-7 | 27 |
|  | - 2.01 | | PSMA4 | Proteasome subunit alpha type-4 | 20 |
|  | - 2.02 | | PSMA1 | Isoform Long Proteasome subunit alpha type-1 | 18 |
|  | - 2.09 | | NME1; | Nucleoside diphosphate kinase | 15 |
|  |  | | NME2 |  |  |
|  | - 1.86 | | ALAD | Delta-aminolevulinic acid dehydratase isoform a | 35 |
|  | - 1.55 | | PSME1 | Proteasome activator subunit 1 | 44 |
|  |  | | PSMA2 | Proteasome subunit, alpha type- 2 | 22 |
|  |  | | PSME2 | Proteasome activator subunit 2 | 2 |
|  | - 1.79 | | ALAD | Delta-aminolevulinic acid dehydratase isoform a | 44 |
|  | - 1.63 | | QDPR | Dihydropteridine reductase | 8 |
|  | - 1.80 | | PSMD7 | 26S proteasome non-ATPase regulatory SU 7 | 8 |
|  | - 1.75 | | G6PD | Glucose-6-phosphate 1-dehydrogenase | 54 |
|  | - 1.67 | | USP14 | Ubiquitin carboxyl-terminal hydrolase 14 | 69 |
| HA24 |  | |  |  |  |
|  | - 1.75 | | RAB10 | Ras-related protein Rab-10 | 5 |
|  | - 1.78 | | PIP4K2A | Phosphatidylinositol-5-phosphate 4-kinase type-2 A | 23 |
|  | - 1.88 | | CAP1 | Adenylyl cyclase-associated protein 1 | 20 |
|  | - 1.78 | | PGAM1 | Phosphoglycerate mutase 1 | 10 |
|  | - 1.72 | | GMPR | GMP reductase 1 | 40 |
|  | - 1.78 | | PURA | Transcriptional activator protein Pur-alpha | 34 |
|  | - 1.71 | | GMPR | GMP reductase 1 | 34 |
|  | - 1.61 | | PSMD12 | 26S proteasome non-ATPase regulatory SU 12 | 40 |
|  | - 1.59 | | PSMC5 | 26S protease regulatory subunit 8 | 56 |
|  | - 1.52 | | CCT2 | Chaperonin containing TCP1, subunit 2 | 27 |

ID: Name of sample set (HA09, HA19, HA21, HA24)

Fold Change: Comparison of normalized volume in Patient sample with average of controls

Gene: HGNC Symbol for coding human gene

Protein: HGNC Symbol for protein identified

SC: Spectral count

SU: Subunit
