# Supplementary material for: Assessment of the Red Cell Proteome of Young Patients with Unexplained Hemolytic Anemia by Two-Dimensional Differential In-Gel Electrophoresis (DIGE)
Source: PLoS One. 2012 Apr 3;7(4):e34237. doi: 10.1371/journal.pone.0034237 (PMC3317954; doi:10.1371/journal.pone.0034237)
Supplement: Table S5 — Expression of Cytoskeleton Proteins. (DOCX) [file pone.0034237.s009.docx]

Table S5: Expression of Cytoskeleton Proteins

| ID | Fold change | Gene | Protein |
| --- | --- | --- | --- |
| HA09 |  |  |  |
|  | + 2.01 | ACTR1A | Alpha-centractin |
|  | + 4.50 | ACTB | Actin, cytoplasmic 2 |
| HA19 |  |  |  |
|  | - 1.84 | CAPZB | Capping protein |
|  | - 2.80 | LCP1 | Lymphocyte cytosolic protein 1 (L-plastin) |
|  | - 2.65 | GDI2 | Rab GDP dissociation inhibitor beta |
|  | - 3.09 | RHOA | Ras homolog gene family, A |
| HA21 |  |  |  |
|  | + 2.47 | ACTR1A | Alpha-centractin |
|  | + 3.59 | TUBB | Tubulin beta chain |
|  |  | TUBB4 | Tubulin beta-4 chain |
|  |  | TUBB6 | Tubulin, beta 6 |
|  |  | CRLF3 | Cytokine receptor-like factor 3 |
|  | + 2.23 | TUBA1B | Tubulin alpha-1B chain |
|  |  | TUBA4A | Tubulin alpha-4A chain |
|  |  | TUBB2C | Tubulin beta-2C chain |
|  | - 2.16 | LCP1 | Lymphocyte cytosolic protein 1 (L-plastin) |
|  | - 2.39 | CAPZB | Capping protein |
|  | - 3.75 | GDI2 | Rab GDP dissociation inhibitor beta |
|  |  | GDI1 | Rab GDP dissociation inhibitor alpha |
|  | - 2.43 | GDI2 | Rab GDP dissociation inhibitor beta |
| HA24 |  |  |  |
|  | + 2.49 | ACTB | Actin, cytoplasmic 2 |
|  |  | actin | actin (no unique peptides) |
|  |  | ACTBL2 | actin, beta-like 2, hypothetical protein |

ID: Name of sample set (HA09, HA19, HA21, HA24)

Fold Change: Comparison of normalized volume in Patient sample with average of controls

Gene: HGNC Symbol for coding human gene Protein: HGNC Symbol for protein identified

SC: Spectral count
