# Supplementary material for: Assessment of the Red Cell Proteome of Young Patients with Unexplained Hemolytic Anemia by Two-Dimensional Differential In-Gel Electrophoresis (DIGE)
Source: PLoS One. 2012 Apr 3;7(4):e34237. doi: 10.1371/journal.pone.0034237 (PMC3317954; doi:10.1371/journal.pone.0034237)
Supplement: Table S6 — Expression levels of Chaperones. (DOCX) [file pone.0034237.s010.docx]

Table S6: Expression levels of Chaperones

| ID | Fold change | | Gene | Protein |
| --- | --- | --- | --- | --- |
| HA09 |  | |  |  |
|  | - 1.62 | | CCT3 | T-complex protein 1 subunit gamma |
|  |  |  | CCT6A | T-complex protein 1 subunit zeta |
|  | - 2.0 | | CCT3 | T-complex protein 1 subunit gamma |
|  | - 1.87 | | CCT6A | T-complex protein 1 subunit zeta |
|  | - 1.96 | | CCT2 | T-complex protein 1 subunit beta |
|  | - 1.74 | | CCT7 | T-complex protein 1 subunit eta |
|  |  |  | CCT4 | T-complex protein 1 subunit delta |
|  |  |  | CCT2 | T-complex protein 1 subunit beta |
|  | - 1.69 | | CCT5 | T-complex protein 1 subunit epsilon |
|  | - 2.08 | | CCT8 | Chaperonin containing TCP1, subunit 8 (theta) |
|  |  |  | CCT5 | T-complex protein 1 subunit epsilon |
|  | + 2.69 | | HSPA8 | Isoform 1 of Heat shock cognate 71 kD protein |
|  | + 2.05 | | HSPA1B | Heat shock 70kDa protein 1A/1B |
|  |  |  | HSPA8 | Isoform 1 of Heat shock cognate 71 kD protein |
|  | + 4.29 | | HSPA1B | Heat shock 70kDa protein 1A/1 |
|  |  |  | HSPA8 | Isoform 1 of Heat shock cognate 71 kD protein |
|  | + 4.31 | | HSP90AA1 | Heat shock protein 90 kD alpha, classA member1 |
|  |  |  | HSP90AB1 | Heat shock protein 90 kD alpha, classA member1 |
| HA19 |  |  | |  |
|  | + 1.88 | CCT4 | | T-complex protein 1 subunit delta |
|  |  | CCT7 | | T-complex protein 1 subunit eta |
|  | + 1.92 | CCT3 | | T-complex protein 1 subunit gamma |
|  | + 1.72 | TCP1 | | T-complex protein 1 subunit alpha |
|  | - 1.85 | HSPA8 | | Isoform 1 of Heat shock cognate 71 kD protein |
|  | + 2.69 | AHSA1 | | Activator of 90 kD heat shock protein ATPase |
| HA21 |  |  | |  |
|  | + 1.77 | CCT4 | | T-complex protein 1 subunit delta |
|  |  | CCT7 | | T-complex protein 1 subunit eta |
|  | + 1.89 | CCT3 | | T-complex protein 1 subunit gamma |
|  | + 1.88 | TCP1 | | T-complex protein 1 subunit alpha |
|  | + 1.66 | CCT2 | | T-complex protein 1 subunit beta |
| HA24 |  |  | |  |
|  | - 1.53 | CCT8 | | Chaperonin containing TCP1, subunit 8 (theta) |
|  | - 1.52 | CCT2 | | T-complex protein 1 subunit beta |

ID: Name of sample set (HA09, HA19, HA21, HA24)

Fold Change: Comparison of normalized volume in Patient sample with average of controls

Gene: HGNC Symbol for coding human gene

Protein: HGNC Symbol for protein identified

SC: Spectral count
