# Supplementary material for: Assessment of the Red Cell Proteome of Young Patients with Unexplained Hemolytic Anemia by Two-Dimensional Differential In-Gel Electrophoresis (DIGE)
Source: PLoS One. 2012 Apr 3;7(4):e34237. doi: 10.1371/journal.pone.0034237 (PMC3317954; doi:10.1371/journal.pone.0034237)
Supplement: Table S7 — Expression of proteins involved in Protein Degradation/Proteasome/Ubiquitin pathway. (DOCX) [file pone.0034237.s011.docx]

Table S7: Expression of proteins involved in Protein Degradation / Proteasome / Ubiquitin pathway

| ID | Fold change | Gene | Protein |
| --- | --- | --- | --- |
| HA09 |  |  |  |
|  | - 2.05 | PSMC5 | 26S protease regulatory subunit 8 |
|  | - 1.55 | PSMA1 | Isoform Long of Proteasome subunit alpha type-1 |
|  | - 1.55 | PSMC6 | 26S protease regulatory subunit S10B |
|  | - 1.58 | PSMB7 | Proteasome subunit beta type-7 |
|  | - 1.62 | PITHD1 | PITH domain containing 1 |
|  | - 2.02 | USP14 | Ubiquitin carboxyl-terminal hydrolase 14 |
| HA19 | - 2.44 | PSMA4 | Proteasome subunit alpha type-4 |
|  |  | PSMD9 | Proteasome 26S subunit, non-ATPase, 9 |
|  | - 2.17 | PSMA1 | Isoform Long of Proteasome subunit alpha type-1 |
|  | - 1.72 | PSMB5 | Proteasome subunit, beta type, 5 |
|  | - 1.50 | PSMB1 | Proteasome subunit beta type-1 |
|  | - 1.51 | PSMB3 | Proteasome subunit beta type-3 |
|  | - 2.09 | PSMA4 | Proteasome subunit alpha type-4 |
|  | - 2.49 | USP14 | Ubiquitin carboxyl-terminal hydrolase 14 |
|  | + 1.68 | PITHD1 | PITH domain containing 1 |
|  | + 2.41 | TXNL1 | Thioredoxin-like 1 |
| HA21 |  |  |  |
|  | - 2.44 | PSMB4 | Proteasome subunit beta type-4 (precursor) |
|  | - 2.01 | PSMA4 | Proteasome subunit alpha type-4 |
|  |  | PSMD9 | Proteasome 26S subunit, non-ATPase, 9 |
|  | - 2.02 | PSMA1 | Proteasome subunit alpha type-4 |
|  | - 1.80 | PSMD7 | 26S proteasome regulatory subunit 7 |
|  | - 2.03 | PSMA7 | Isoform 1 of proteasome subunit alpha type-7 |
|  | - 2.20 | PSMA4 | Proteasome subunit alpha type-4 |
|  | - 1.55 | PSME1 | Proteasome activator subunit 1 |
|  |  | PSMA2 | Proteasome subunit, alpha type- 2 |
|  |  | PSME2 | Proteasome activator subunit 2 |
|  | - 2.32 | CUL4A | Cullin-4A |
|  |  | CUL5 | Cullin-5 |
|  | - 1.67 | USP14 | Ubiquitin carboxyl-terminal hydrolase 14 |
|  | + 1.52 | PSMC5 | 26S protease regulatory subunit 8 |
|  | + 2.19 | PITHD1 | PITH domain containing 1 |
|  | + 2.33 | TXNL1 | Thioredoxin-like 1 |
| HA24 |  |  |  |
|  | - 1.60 | PSMD12 | 26S proteasome non-ATPase regulatory subunit 12 |
|  | - 1.59 | PSMC5 | 26S protease regulatory subunit 8 |

ID: Name of sample set (HA09, HA19, HA21, HA24)

Fold Change: Comparison of normalized volume in Patient sample with average of controls

Gene: HGNC Symbol for coding human gene

Protein: HGNC Symbol for protein identified SC: Spectral count
