# Supplementary material for: Assessment of the Red Cell Proteome of Young Patients with Unexplained Hemolytic Anemia by Two-Dimensional Differential In-Gel Electrophoresis (DIGE)
Source: PLoS One. 2012 Apr 3;7(4):e34237. doi: 10.1371/journal.pone.0034237 (PMC3317954; doi:10.1371/journal.pone.0034237)
Supplement: Table S8 — Complete list of all spots picked and proteins identified in HA09. (DOCX) [file pone.0034237.s012.docx]

# Table S8: Complete list of all spots picked and proteins identified in HA09

| **Spot rank** | **Pick #** | **Spot Location**  **(kD / pI)** | **Coverage**  **(%)** | **Gene** | **Protein** | **SC** |
| --- | --- | --- | --- | --- | --- | --- |
| 42 | 1 | 80 / 6.9 | 30.3 | *HSPA8* | Isoform 1 of Heat shock cognate 71kDa protein | 56 |
|  |  |  |  |  | (70.8kD, pI 5.5) |  |
| 52 | 2 | 73 / 6.1 | 5.3 | *GCLC* | Glutamate--cysteine ligase catalytic subunit | 2 |
|  |  |  |  |  | (72.7kD, pI 6.1) |  |
| 160 | 3 | 55 / 7.0 | 13.4 | *G6PD* | Isoform Long of Glucose-6-phosphate 1-dehydrogenase | 6 |
|  |  |  |  |  | (63.8kD, pI 6.9) |  |
| 59 | 4 | 75 / 7.0 |  |  | NO ID |  |
| 111 | 5 | 60/ 6.5 | 8 | *TXNRD1* | Thioredoxin reductase 1 isoform 3 (71.1kD, pI 5.5) | 4 |
|  |  |  | 4.7 | *FBXO7* | F-box only protein 7 (58.2kD, pI 6.8) | 10 |
|  |  |  | 5.6 | *ALDH1A1* | Retinal dehydrogenase 1 (54.8kD, pI 6.7) | 3 |
| 84 | 6 | 75 / 6.8 |  |  | NO ID |  |
| 79 | 7 | 58 / 6.2 | 16.7 | *OXSR1* | Serine/threonine-protein kinase OSR1 (58kD. pI 6.4) | 10 |
| 69 | 8 | 60 / |  |  | NO ID |  |
| 133 | 9 | 58 / 6.9 | 39.6 | *G6PD* | Isoform Long of Glucose-6-phosphate 1-dehydrogenase | 48 |
|  |  |  |  |  | (63.8kD. pI 6.9) |  |
| 82 | 10 | 55 / 7.5 | 6.1 | *CAP1* | Adenylyl cyclase-associated protein 1 (58.1kD, pI 8.1) | 3 |
|  |  |  | 2.7 | *G6PD* | Isoform Long of Glucose-6-phosphate 1-dehydrogenase | 3 |
|  |  |  |  |  | (63.8kD. pI 6.9) |  |
| 175 | 11 |  |  |  | NO ID |  |
| 166 | 12 | 27 / 5.8 | 32.2 | *PITHD1* | PITH domain-containing protein 1 (24kD, pI 5.8) | 18 |
| 76 | 13 | 45 / 7.0 | 29.3 | *ACTR1A* | Alpha-centractin (42.6kD, pI 6.6) | 21 |
|  |  |  | 13.7 | *PITHD1* | PITH domain-containing protein 1 (24kD, 5.8) | 4 |
|  |  |  |  |  | CARRY OVER |  |
| 193 | 14 | 30 / 7 | 17.7 | *PSMD8* | Proteasome 26S non-ATPase subunit 8 (39.6kD, pI 9.7) | 14 |
|  |  |  | 21.2 | GMPR | GMP reductase 1 (37.4kD, pI 7.1) | 7 |
|  |  |  | 19.4 | *ACTR1A* | Alpha-centractin (42.6kD, pI 6.6) CARRY OVER | 7 |
| 141 | 15 |  |  |  | NO ID |  |
| 116 | 16 | 45 / 6.1 | 14.7 | *PPME1* | Isoform 1 of Protein phosphatase methylesterase 1 | 15 |
|  |  |  |  |  | (42.6kD, pI 5.8) |  |
| 109 | 17 | 70 / 5.6 | 19 | *HSPA1B* | HSPA1A Heat shock 70 kDa protein 1 (70kD, pI 5.6) | 50 |
|  |  |  | 19.3 | *HSPA8* | Isoform 1 of Heat shock cognate 71kDa protein | 23 |
|  |  |  |  |  | (70.8kD, pI 5.5) |  |
|  |  |  | 10.3 | *HSPA1L* | Heat shock 70 kDa protein 1L (70.4kD, 6.5) | 8 |
|  |  |  |  |  | no unique peptides |  |
| 45 | 18 | 45 / 6.9 | 11.6 | *PA2G4* | Proliferation-associated protein 2G4(45.1kD, 8.9) | 4 |
|  |  |  | 7.6 | *EEF1G* | Elongation factor 1-gamma (50.1kD, pI 6.7) | 4 |
| 132 | 19 | 45 / 7.5 | 47 / 7.5 | *PSMC5* | 26S protease regulatory subunit 8 (45.6kD, pI 7.5) | 37 |
| 281 | 20 | 32 / 6.9 | 33.1 | *PSMA1* | Isoform Long of Proteasome subunit alpha type-1 | 12 |
|  |  |  |  |  | (30.2Kd, pI 7) |  |
| 285 | 21 | 35 / 6.1 | 42.5 | *CAPZB* | Capping protein (33.7kD, pI 6.4) | 18 |
|  |  |  | 9.6 | *PNP* | Purine Nucleoside Phosphorylase (32.5kD, pI 7.2) | 3 |
| 188 | 22 | 32 / 5.5 | 17.6 | *CAPZB* | Capping protein (33.7kD, pI 6.4) carry over | 7 |
|  |  |  | 8.1 | *PSMC5* | 26S protease regulatory subunit 8 (45.6kD, pI 7.5) | 3 |
| 159 | 23 | 60 / 6.7 | 30.1 | *CCT3* | T-complex protein 1 subunit gamma (60.5 kD, pI 6.5) | 32 |
|  |  |  | 7 | *CCT6A* | T-complex protein 1 subunit zeta (58kD, pI 6.7) | 4 |
| 119 | 24 | 60 / 6.2 | 9 | *CCT3* | T-complex protein 1 subunit gamma (60.5 kD, pI 6.5) | 6 |
| 199 | 25 | 40 /5.8 | 26 | *GLRX3* | Glutaredoxin-3 (37kD, pI 5.4) | 8 |
|  |  |  | 3.5 | *LETM1* | Leucine zipper-EF-hand-containing transmem.protein 1, | 2 |
|  |  |  |  |  | mitochondrial precursor (83.3kD, pI 6.7) |  |
| 419 | 26 | 50 / 6.5 | 32 | *BLMH* | Bleomycin hydrolase (52.5kD, pI 6.3) | 41 |
|  |  |  | 22 | *GDI2* | Rab GDP dissociation inhibitor beta (50.6kD, pI 6.5) | 9 |
| 139 | 27 | 60 / 6.7 | 6.4 | *CCT6A* | T-complex protein 1 subunit zeta (58kD, pI 6.7) | 2 |
| 75 | 28 |  |  |  | NO ID |  |
| 347 | 29 | 27 / 8.8 | 39.5 | *PSMA7* | Isoform 1 of Proteasome subunit alpha type-7 | 23 |
|  |  |  |  |  | (27.8kD, pI 8.5) |  |
| 232 | 30 | 50 / 6.3 | 14.5 | *PA2G4* | Proliferation-associated protein 2G4(45.1kD, 8.9) | 6 |
| 327 | 31 | 42 /7.5 | 42.9 | *PSMC6* | 26S protease regulatory subunit S10B (44.1kD, 7.5) | 39 |
| 196 | 32 | 33 / 6.4 | 47 | *PNP* | Purine Nucleoside Phosphorylase (32.5kD, pI 7.2) | 52 |
| 307 | 33 | 50 / 6.1 | 26.8 | *PA2G4* | Proliferation-associated protein 2G4(45.1kD, 8.9) | 26 |
|  |  |  | 15.5 | *PSMC2* | 26S protease regulatory subunit 7 (48.5kD, pI 5.9) | 5 |
| 154 | 34 | 55 / 6.5 | 35.7 | *CCT2* | T-complex protein 1 subunit beta (57.5kD, pI 6.4) | 40 |
|  |  |  | 3.8 | *DARS* | Aspartyl-tRNA synthetase, cytoplasmic (57.1kD, pI 6.5) | 3 |
| 145 | 35 | 60 / 7.5 | 28.2 | *CCT7* | T-complex protein 1 subunit eta (59.3kD, pI 7.6) | 29 |
|  |  |  | 13.9 | *CCT4* | T-complex protein 1 subunit delta (57.9kD, pI 7.8) | 14 |
|  |  |  | 12 | *CCT2* | T-complex protein 1 subunit beta (57.5kD, pI 6.4) | *7* |
| 256 | 36 | 30 / 6.1 | 32.9 | *PSMB7* | Proteasome subunit beta type-7 precursor | 16 |
|  |  |  |  |  | (29.9kD, pI7.7) |  |
|  |  |  | 16.1 | *GCLM* | Glutamate--cysteine ligase regulatory subunit | 5 |
|  |  |  |  |  | (30.7kD, pI 6.0) |  |
|  |  |  | 2.4 | *ALB* | Uncharacterized protein ALB (71.5kD, pI 6.7) | 4 |
| 384 | 37 | 49 / 6.1 | 10.9 | *PSMC2* | 26S protease regulatory subunit 7 (48.5kD, pI 5.9) | 6 |
| 201 | 38 | 58 / 5.7 | 13.5 | *CCT5* | T-complex protein 1 subunit epsilon (59.6kD, pI 5.6) | 8 |
| 255 | 39 | 39 / 7.6 | 16.7 | *ALAD* | Delta-aminolevulinic acid dehydratase isoform a | 10 |
|  |  |  |  |  | (39kD, pI 7.6) |  |
| 122 | 40 | 57 / 5.6 | 21.5 | *CCT8* | Chaperonin containing TCP1, subunit 8 (59.8kD, pI 5.6) | 16 |
|  |  |  | 15 | *CCT5* | T-complex protein 1 subunit epsilon (59.6kD. pI 5.6) | 12 |
| 17 | 41 | 71 / 5.6 | 24.3 | *HSPA1B* | HSPA1A Heat shock 70 kDa protein 1 (70kD, pI 5.6) | 52 |
|  |  |  | 13.8 | *HSPA8* | Isoform 1 of Heat shock cognate 71 kDa protein | 10 |
|  |  |  |  |  | (70.8kD, pI 5.5) |  |
| 124 | 42 |  |  |  | NO ID |  |
| 114 | 43 | 56 / 5.3 | 13 | *USP14* | Ubiquitin carboxyl-terminal hydrolase 14 (56kD, 5.3) | 8 |
| 18 | 44 | 80 / 5.5 | 18.6 | *HSP90AA1* | Heat shock protein 90kDa alpha (cytosolic), | 33 |
|  |  |  |  |  | class A member 1 (98kD, pI 5.2) |  |
|  |  |  | 8.1 | *HSP90AB1* | 85kDa protein (84.7kD, pI 5.4) | 4 |
|  |  |  | 4.2 | *TGM2* | Isoform 1 of Protein-glutamine gamma- | 3 |
|  |  |  |  |  | glutamyltransferase 2 (77.3kD, pI 5.2) |  |
| 16 | 45 | 45 /5.8 | 25.7 | *ACTB* | Actin, cytoplasmic 2 (45kD, pI 6.2) | 21 |
| 267 | 46 | 39 / 7.0 | 28 | *ALAD* | Delta-aminolevulinic acid dehydratase isoform a | 31 |
|  |  |  |  |  | (39kD, pI 7.6) |  |
|  |  |  | 9.5 | *AKR7A2* | Aflatoxin B1 aldehyde reductase member 2 | 3 |
|  |  |  |  |  | (39.5kD, pI 7.2) |  |
|  |  |  | 0.2 | *HERC1* | Guanine nucleotide exchange factor p532 (532kD,pI 6.0) | 2 |
| 118 | 47 | 27.7 / 4.8 | 18.4 | *YWHAZ* | 14-3-3 protein zeta/delta (27.7kD, pI 4.8) | 7 |
|  |  |  | 11.2 | *PSMA5* | Proteasome subunit alpha type-5 (26.4kD, pI 4.8) | 3 |
|  |  |  | 10.2 | *YWHAH* | 14-3-3 protein eta (28.2kD, pI 4.8) | 4 |
|  |  |  | 9.7 | *YWHAG* | 14-3-3 protein gamma (28.3kD, pI 4.9) | 4 |
| 220 | 48 | 22 / 6.0 | 68.2 | *PRDX2* | Peroxiredoxin-2 (21.8kD, pI 6.0) | 180 |
|  |  |  | 4.8 | *PRDX1* | Peroxiredoxin-1 (22.1kD, pI 8.1) | 3 |

Spot rank: Rank of spot as assigned by Same Spots Software depending on fold change (normalized volume) comparing the highest to lowest sample

Pick #: Sequence in which spots were excised from gel depending on spot intensity (from lowest to highest)

Spot Location: Approximate location of spot by protein mass (marker on gel in kilo dalton, kD) and isoelectric point (pI) as estimated on focussing strip used

Coverage: Percentage of aminoacid sequence that is covered by unique peptides to identify protein

Gene: HGNC Symbol for coding human gene

Protein: HGNC Symbol for protein identified

SC: Spectral count
