# Supplementary material for: Assessment of the Red Cell Proteome of Young Patients with Unexplained Hemolytic Anemia by Two-Dimensional Differential In-Gel Electrophoresis (DIGE)
Source: PLoS One. 2012 Apr 3;7(4):e34237. doi: 10.1371/journal.pone.0034237 (PMC3317954; doi:10.1371/journal.pone.0034237)
Supplement: Table S9 — Complete list of all spots picked in HA19, fold change comparing patient to all other samples run in the experiment. (DOCX) [file pone.0034237.s013.docx]

# Table S9: Complete list of all spots picked in HA19, fold change comparing patient to all other samples run in the experiment

| Spot rank | Pick # | Fold change |  | Patient | Mother | Control | Standard |
| --- | --- | --- | --- | --- | --- | --- | --- |
| 42 | 1 |  | I | 2.028 | 0.908 | 0.573 | 0.785 |
|  |  |  | II | 2.000 | 0.920 | 0.588 | 0.723 |
|  |  |  | med | 2.014 | 0.914 | 0.581 | 0.754 |
|  | **P to all 2.69** | |  |  | **2.20** | **3.47** | **2.67** |
| 52 | 2 |  | I | 0.557 | 1.682 | 0.910 | 0.993 |
|  |  |  | II | 0.517 | 1.723 | 0.907 | 1.078 |
|  |  |  | med | 0.537 | 1.703 | 0.909 | 1.036 |
|  | *P to all 2.26* | |  |  | *3.170* | *1.69* | *1.93* |
| 160 | 3 |  | I | 0.553 | 0.899 | 1.105 | 1.221 |
|  |  |  | II | 0.630 | 0.793 | 1.176 | 1.125 |
|  |  |  | med | 0.592 | 0.846 | 1.141 | 1.173 |
|  | *P to all 1.78* | |  |  | *1.430* | *1.93* | *1.98* |
| 59 | 4 |  | I | 0.420 | 1.068 | 1.212 | 0.827 |
|  |  |  | II | 0.418 | 1.108 | 1.302 | 0.859 |
|  |  |  | med | 0.419 | 1.088 | 1.257 | 0.843 |
|  | *P to all 2.54* | |  |  | *2.597* | *3.00* | *2.01* |
| 111 | 5 |  | I | 0.503 | 1.037 | 1.058 | 0.763 |
|  |  |  | II | 0.515 | 1.281 | 1.023 | 0.877 |
|  |  |  | med | 0.509 | 1.159 | 1.041 | 0.820 |
|  | *P to all 1.98* | |  |  | *2.277* | *2.04* | *1.61* |
| 84 | 6 |  | I | 0.607 | 1.641 | 0.896 | 1.164 |
|  |  |  | II | 0.601 | 1.621 | 0.908 | 1.214 |
|  |  |  | med | 0.604 | 1.631 | 0.902 | 1.189 |
|  | *P to all 2.05* | |  |  | *2.700* | *1.49* | *1.97* |
| 79 | 7 |  | I | 0.455 | 1.053 | 1.264 | 0.897 |
|  |  |  | II | 0.416 | 1.147 | 1.155 | 0.954 |
|  |  |  | med | 0.436 | 1.100 | 1.210 | 0.926 |
|  | *P to all 2.48* | |  |  | *2.526* | *2.78* | *2.13* |
| 69 | 8 |  | I | 1.345 | 1.054 | 0.714 | 0.477 |
|  |  |  | II | 1.441 | 1.076 | 0.640 | 0.507 |
|  |  |  | med | 1.393 | 1.065 | 0.677 | 0.492 |
|  | **P to all 1.87** | |  |  | **1.31** | **2.06** | **2.83** |
| 133 | 9 |  | I | 0.462 | 1.003 | 1.097 | 0.931 |
|  |  |  | II | 0.529 | 0.996 | 0.994 | 1.011 |
|  |  |  | med | 0.496 | 1.000 | 1.046 | 0.971 |
|  | *P to all 2.03* | |  |  | *2.017* | *2.11* | *1.96* |
| 82 | 10 |  | I | 0.521 | 0.890 | 1.288 | 1.332 |
|  |  |  | II | 0.471 | 0.842 | 1.196 | 1.407 |
|  |  |  | med | 0.496 | 0.866 | 1.242 | 1.370 |
|  | *P to all 2.34* | |  |  | *1.746* | *2.50* | *2.76* |
| 175 | 11 |  | I | 0.609 | 1.066 | 1.152 | 1.235 |
|  |  |  | II | 0.665 | 0.957 | 1.062 | 1.205 |
|  |  |  |  | 0.637 | 1.012 | 1.107 | 1.220 |
|  | *P to all 1.75* | | *med* |  | *1.588* | *1.74* | *1.92* |
| 166 | 12 |  | I | 0.618 | 1.275 | 1.031 | 0.781 |
|  |  |  | II | 0.624 | 1.132 | 0.998 | 0.808 |
|  |  |  | med | 0.621 | 1.204 | 1.015 | 0.795 |
|  | *P to all 1.62* | |  |  | *1.938* | *1.63* | *1.28* |
| 76 | 13 |  | I | 1.419 | 0.676 | 0.945 | 0.480 |
|  |  |  | II | 1.442 | 0.667 | 0.947 | 0.556 |
|  |  |  | med | 1.431 | 0.672 | 0.946 | 0.518 |
|  | **P to all 2.01** | |  |  | **2.13** | **1.51** | **2.76** |
| 193 | 14 |  | I | 0.586 | 0.885 | 1.147 | 0.903 |
|  |  |  | II | 0.596 | 0.944 | 1.024 | 1.021 |
|  |  |  | med | 0.591 | 0.915 | 1.086 | 0.962 |
|  | *P to all 1.67* | |  |  | *1.547* | *1.84* | *1.63* |
| 141 | 15 |  | I | 0.498 | 0.994 | 1.143 | 1.020 |
|  |  |  | II | 0.573 | 1.119 | 1.093 | 0.990 |
|  |  |  | med | 0.536 | 1.057 | 1.118 | 1.005 |
|  | *P to all 1.98* | |  |  | *1.973* | *2.09* | *1.88* |
| 116 | 16 |  | I | 0.547 | 1.217 | 0.990 | 0.959 |
|  |  |  | II | 0.534 | 1.193 | 1.010 | 0.986 |
|  |  |  | med | 0.541 | 1.205 | 1.000 | 0.973 |
|  | *P to all 1.96* | |  |  | *2.229* | *1.85* | *1.80* |
| 109 | 17 |  | I | 1.656 | 0.807 | 0.736 | 0.795 |
|  |  |  | II | 1.465 | 0.879 | 0.632 | 0.718 |
|  |  |  | med | 1.561 | 0.843 | 0.684 | 0.757 |
|  | **P to all 2.05** | |  |  | **1.85** | **2.28** | **2.06** |
| 45 | 18 |  | II | 1.540 | 0.429 | 0.801 | 0.687 |
|  |  |  | II | 1.277 | 0.413 | 0.657 | 0.752 |
|  |  |  | med | 1.409 | 0.421 | 0.729 | 0.720 |
|  | **P to all 2.26** | |  |  | **3.35** | **1.93** | **1.96** |
| 132 | 19 |  | I | 0.538 | 1.028 | 1.161 | 1.106 |
|  |  |  | II | 0.532 | 0.998 | 1.101 | 1.192 |
|  |  |  |  | 0.535 | 1.013 | 1.131 | 1.149 |
|  | *P to all 2.05* | | *med* |  | *1.893* | *2.11* | *2.15* |
| 281 | 20 |  | I | 0.639 | 0.944 | 0.941 | 1.024 |
|  |  |  | II | 0.609 | 0.959 | 0.850 | 1.068 |
|  |  |  | med | 0.624 | 0.952 | 0.896 | 1.046 |
|  | *P to all 1.55* | |  |  | *1.525* | *1.44* | *1.68* |
| 285 | 21 |  | I | 0.724 | 1.278 | 1.002 | 0.943 |
|  |  |  | II | 0.696 | 1.080 | 1.022 | 0.854 |
|  |  |  | med | 0.710 | 1.179 | 1.012 | 0.899 |
|  | P to all 1.45 | |  |  | 1.661 | 1.43 | 1.27 |
| 188 | 22 |  | I | 0.910 | 1.081 | 1.191 | 0.650 |
|  |  |  | II | 0.898 | 1.060 | 1.195 | 0.647 |
|  |  |  | med | 0.904 | 1.071 | 1.193 | 0.649 |
|  | *P to M 1.07* | |  |  | *1.184* | *1.32* | *1.39* |
| 159 | 23 |  | I | 0.610 | 1.266 | 1.066 | 0.735 |
|  |  |  | II | 0.638 | 1.205 | 1.077 | 0.715 |
|  |  |  | med | 0.624 | 1.236 | 1.072 | 0.725 |
|  | *P to all 1.62* | |  |  | *1.980* | *1.72* | *1.16* |
| 119 | 24 |  | I | 0.525 | 1.246 | 1.067 | 0.938 |
|  |  |  | II | 0.547 | 1.127 | 1.112 | 0.929 |
|  |  |  | med | 0.536 | 1.187 | 1.090 | 0.934 |
|  | *P to all 2.00* | |  |  | *2.214* | *2.03* | *1.74* |
| 199 | 25 |  | I | 0.597 | 1.076 | 1.141 | 0.886 |
|  |  |  | II | 0.609 | 1.115 | 0.991 | 0.881 |
|  |  |  | med | 0.603 | 1.096 | 1.066 | 0.884 |
|  | *P to all 1.68* | |  |  | *1.817* | *1.77* | *1.47* |
| 419 | 26 |  | I | 0.760 | 0.832 | 0.965 | 1.019 |
|  |  |  | II | 0.750 | 0.863 | 0.913 | 1.063 |
|  |  |  | med | 0.755 | 0.848 | 0.939 | 1.041 |
|  | P to all 1.25 | |  |  | 1.123 | 1.24 | 1.38 |
| 139 | 27 |  | I | 0.500 | 1.124 | 1.082 | 0.768 |
|  |  |  | II | 0.539 | 1.049 | 1.022 | 0.771 |
|  |  |  | med | 0.520 | 1.087 | 1.052 | 0.770 |
|  | *P to all 1.87* | |  |  | *2.091* | *2.03* | *1.48* |
| 78 | 28 |  | I | 0.426 | 1.203 | 1.140 | 0.939 |
|  |  |  | II | 0.428 | 1.193 | 1.153 | 1.056 |
|  |  |  | med | 0.427 | 1.198 | 1.147 | 0.998 |
|  | *P to all 2.61* | |  |  | *2.806* | *2.69* | *2.34* |
| 347 | 29 |  | I | 0.830 | 1.078 | 1.144 | 1.263 |
|  |  |  | II | 0.871 | 1.204 | 1.130 | 1.354 |
|  |  |  | med | 0.851 | 1.141 | 1.137 | 1.309 |
|  | P to all 1.41 | |  |  | 1.342 | 1.34 | 1.54 |
| 232 | 30 |  | I | 0.637 | 0.860 | 1.113 | 1.011 |
|  |  |  | II | 0.610 | 0.833 | 1.074 | 1.123 |
|  |  |  | med | 0.624 | 0.847 | 1.094 | 1.067 |
|  | *P to all 1.61* | |  |  | *1.358* | *1.75* | *1.71* |
| 327 | 31 |  | I | 0.646 | 1.028 | 1.060 | 1.028 |
|  |  |  | II | 0.684 | 1.024 | 0.998 | 1.056 |
|  |  |  | med | 0.665 | 1.026 | 1.029 | 1.042 |
|  | *P to all 1.55* | |  |  | *1.543* | *1.55* | *1.57* |
| 196 | 32 |  | I | 1.145 | 0.705 | 0.609 | 1.014 |
|  |  |  | II | 1.117 | 0.687 | 0.637 | 0.984 |
|  |  |  | med | 1.131 | 0.696 | 0.623 | 0.999 |
|  | P to all 1.46 | |  |  | 1.63 | 1.82 | 1.13 |
| 307 | 33 |  | I | 0.605 | 0.810 | 1.022 | 0.991 |
|  |  |  | II | 0.644 | 0.776 | 0.993 | 1.027 |
|  |  |  | med | 0.625 | 0.793 | 1.008 | 1.009 |
|  | *P to all 1.50* | |  |  | *1.270* | *1.61* | *1.62* |
| 154 | 34 |  | I | 0.559 | 0.976 | 1.081 | 1.050 |
|  |  |  | II | 0.503 | 0.970 | 1.077 | 1.105 |
|  |  |  | med | 0.531 | 0.973 | 1.079 | 1.078 |
|  | *P to all 1.96* | |  |  | *1.832* | *2.03* | *2.03* |
| 145 | 35 |  | I | 0.633 | 1.352 | 1.361 | 0.722 |
|  |  |  | II | 0.667 | 1.337 | 1.292 | 0.733 |
|  |  |  | med | 0.650 | 1.345 | 1.327 | 0.728 |
|  | *P to all 1.74* | |  |  | *2.068* | *2.04* | *1.12* |
| 256 | 36 |  | I | 0.612 | 0.985 | 0.899 | 0.962 |
|  |  |  | II | 0.624 | 1.116 | 0.873 | 1.021 |
|  |  |  | med | 0.618 | 1.051 | 0.886 | 0.992 |
|  | *P to all 1.58* | |  |  | *1.700* | *1.43* | *1.60* |
| 384 | 37 |  | I | 0.683 | 0.922 | 0.968 | 0.959 |
|  |  |  | II | 0.689 | 0.889 | 1.007 | 0.976 |
|  |  |  | med | 0.686 | 0.906 | 0.988 | 0.968 |
|  | P to all 1.39 | |  |  | 1.320 | 1.44 | 1.41 |
| 201 | 38 |  | I | 0.606 | 1.165 | 0.985 | 1.087 |
|  |  |  | II | 0.637 | 1.092 | 0.926 | 1.038 |
|  |  |  | med | 0.622 | 1.129 | 0.956 | 1.063 |
|  | *P to all 1.69* | |  |  | *1.816* | *1.54* | *1.71* |
| 255 | 39 |  | I | 0.719 | 1.194 | 1.009 | 1.179 |
|  |  |  | II | 0.703 | 1.217 | 1.015 | 1.258 |
|  |  |  | med | 0.711 | 1.206 | 1.012 | 1.219 |
|  | *P to all 1.61* | |  |  | *1.695* | *1.42* | *1.71* |
| 122 | 40 |  | I | 0.504 | 1.091 | 1.033 | 1.070 |
|  |  |  | II | 0.512 | 1.147 | 0.942 | 1.045 |
|  |  |  | med | 0.508 | 1.119 | 0.988 | 1.058 |
|  | *P to all 2.08* | |  |  | *2.203* | *1.94* | *2.08* |
| 17 | 41 |  | I | 2.283 | 0.600 | 0.418 | 0.515 |
|  |  |  | II | 2.138 | 0.639 | 0.409 | 0.512 |
|  |  |  | med | 2.211 | 0.620 | 0.414 | 0.514 |
|  | **P to all 4.29** | |  |  | **3.57** | **5.35** | **4.30** |
| 124 | 42 |  | I | 0.681 | 1.398 | 1.057 | 1.101 |
|  |  |  | II | 0.664 | 1.527 | 1.057 | 1.176 |
|  |  |  | med | 0.673 | 1.463 | 1.057 | 1.139 |
|  | *P to all 1.81* | |  |  | *2.175* | *1.57* | *1.69* |
| 114 | 43 |  | I | 0.528 | 1.226 | 1.148 | 1.046 |
|  |  |  | II | 0.582 | 1.255 | 1.056 | 1.005 |
|  |  |  | med | 0.555 | 1.241 | 1.102 | 1.026 |
|  | *P to all 2.02* | |  |  | *2.235* | *1.99* | *1.85* |
| 18 | 44 |  | I | 2.323 | 0.641 | 0.414 | 0.518 |
|  |  |  | II | 2.338 | 0.682 | 0.450 | 0.541 |
|  |  |  | med | 2.331 | 0.662 | 0.432 | 0.530 |
|  | **P to all 4.31** | |  |  | **3.52** | **5.39** | **4.40** |
| 16 | 45 |  | I | 2.251 | 0.435 | 0.419 | 0.642 |
|  |  |  | II | 2.296 | 0.438 | 0.418 | 0.678 |
|  |  |  | med | 2.274 | 0.437 | 0.419 | 0.660 |
|  | **P to all 4.50** | |  |  | **5.21** | **5.43** | **3.44** |
| 267 | 46 |  | I | 0.760 | 1.182 | 0.976 | 1.192 |
|  |  |  | II | 0.713 | 1.190 | 1.006 | 1.314 |
|  |  |  | med | 0.737 | 1.186 | 0.991 | 1.253 |
|  | *P to all 1.55* | |  |  | *1.610* | *1.35* | *1.70* |
| 118 | 47 |  | I | 1.426 | 0.655 | 0.934 | 1.383 |
|  |  |  | II | 1.424 | 0.638 | 0.962 | 1.383 |
|  |  |  | med | 1.425 | 0.647 | 0.948 | 1.383 |
|  | P to all 1.44 | |  |  | 2.20 | 1.50 | 1.03 |
| 220 | 48 |  | I | 0.664 | 1.181 | 0.946 | 1.009 |
|  |  |  | II | 0.681 | 1.214 | 0.942 | 1.023 |
|  |  |  | med | 0.673 | 1.198 | 0.944 | 1.016 |
|  | *P to all 1.57* | |  |  | *1.781* | *1.40* | *1.51* |

Spot rank: Rank of spot as assigned by Same Spots Software depending on fold change (normalized volume) comparing the highest to lowest sample

Pick #: Sequence in which spots were excised from gel depending on spot intensity (from lowest to highest)

Roman Numerals: Normalized volume measured in replicate runs one (I) and two (II)

Med: Average of normalized volume measured in replicate runs one (I) and two (II)

Fold Change P to all: Expression level (normalized volume) of patient sample compared to the average of all other samples combined;

Last row of columns also shows fold change comparing patient to sample indicated in header of column (Mother, Father, Control, Standard)

Formatting: **Bold**: Patient upregulated *Italic*: Patient downregulated

No special formatting: Fold change “P to all” does not exceed 1.5 fold
