# Supplementary material for: Assessment of the Red Cell Proteome of Young Patients with Unexplained Hemolytic Anemia by Two-Dimensional Differential In-Gel Electrophoresis (DIGE)
Source: PLoS One. 2012 Apr 3;7(4):e34237. doi: 10.1371/journal.pone.0034237 (PMC3317954; doi:10.1371/journal.pone.0034237)
Supplement: Table S10 — Complete list of all spots picked and Proteins identified in HA19. (DOCX) [file pone.0034237.s014.docx]

# Table S10: Complete list of all spots picked and Proteins identified in HA19

| **Spot rank** | **Pick #** | **Spot Location**  **(kD / pI)** | **Coverage**  **(%)** | Gene | **Protein** | SC |
| --- | --- | --- | --- | --- | --- | --- |
| 6 | 1 | 80 / 6.8 | 1.5 | *MUC5AC* | *Mucin 5 (*Fragment) (261kD, pI 6.6) | 2 |
| 38 | 2 | 45 / 7.5 | 24.4 | *CSNK2A1* | Casein kinase 2, alpha 1 polypeptide (45.9kD, pI 7.9) | 14 |
|  |  |  | 9.8 | *PSMC6* | 26S protease regulatory subunit S10B (44.1kD, pI 7.5) | 10 |
|  |  |  | 3.3 | *TARS* | Threonyl-tRNA synthetase, cytoplasmic (83.4kD, pI 6.7) | 2 |
| 22 | 3 | 110 / 6.5 | 6.1 | *XPO7* | Exportin 7 isoform a (125kD, pI 6.3) | 8 |
| 16 | 4 | 30 / 5.5 | 38.2 | *CLIC1* | Chloride intracellular channel protein 1 (29.9kD, pI 5.2) | 20 |
| 4 | 5 | 110 / 6.5 | 7 | *XPO7* | Exportin 7 isoform a (125kD, pI 6.3) | 10 |
| 30 | 6 | 110 / 6.5 | 4.2 | *XPO7* | Exportin 7 isoform a (125kD, pI 6.3) | 7 |
| 54 | 7 | 45 / 7.8 | 17.8 | *FH* | Isoform Mito.of Fumarate hydratase, mitoch, precursor | 16 |
|  |  |  |  |  | (54.6kD, pI 8.8) |  |
| 1 | 8 | 33 / 6.5 | 47.4 | *PNP* | Purin Nucleotide Phosphorylase (33kD, pI 7.2) | 23 |
|  |  |  | 9 | *FH* | Isoform Mito.of Fumarate hydratase, mitoch, precursor | 3 |
|  |  |  |  |  | (54.6kD, pI 8.8) carry over |  |
| 9 | 9 | 60 / 6.5 | 11 | *OXSR1* | Serine/threonine-protein kinase OSR1 | 4 |
|  |  |  |  |  | (oxidative stress response 1) (58kD, pI 6.4) |  |
|  |  |  | 8.9 | *TXNRD1* | Thioredoxin reductase 1 isoform 3 (71.1kD, pI 7.5) | 6 |
|  |  |  | 7.4 | *FBXO7* | F-box only protein 7 (58kD, pI 6.8) | 10 |
| 52 | 10 | 40 / 5.5 | 54.6 | *EIF2S1* | Eukaryotic translation initiation factor 2 subunit 1 (36.1kD, pI 5.1) | 35 |
|  |  |  | 12.4 | *NMI* | N-myc (and STAT) interactor (35 kD, pI 5.3) | 2 |
|  |  |  | 12.3 | *PPP2CA* | Serine/threonine-protein phosphatase 2A catalytic subunit | 3 |
|  |  |  |  |  | alpha isoform (35.5kD, pI 5.5) |  |
| 48 | 11 | 45 / 5.8 | 9.8 | *AHSA1* | Activator of 90 kDa heat shock protein ATPase homolog 1 yeast | 7 |
|  |  |  |  |  | (38.2kD, pI 5.5) |  |
| 11 | 12 | 40 / 5.0 | 31.5 | *LOC387867* | Uncharacterized protein (32.7kD, pI 4.5) | 13 |
|  |  |  | 15.7 | *AHSA1* | Activator of 90 kDa heat shock protein ATPase homolog 1 | 7 |
|  |  |  |  |  | (38.2kD, pI 5.5) |  |
|  |  |  | 33.3 | *RPSA* | Ribosomal protein SA (33.3kD, pI 4.9) | 13 |
|  |  |  | 11.4 | *WDR77* | Methylosome protein 50 (36.7kD, pI 5.2) (WD repeat domain 77) | 2 |
| 28 | 13 | 60 / 6.5 | 14.7 | *TXNRD1* | Thioredoxin reductase 1 isoform 3 (71.1kD, pI 7.5) | 9 |
|  |  |  | 14.2 | *OXSR1* | Serine/threonine-protein kinase OSR1 | 12 |
|  |  |  |  |  | (oxidative stress response 1) (58kD, pI 6.4) |  |
| 45 | 14 | 30 / 7.5 | 35.6 | *PSMA4* | Proteasome subunit alpha type-4 (29.4kD, pI 7.7) | 27 |
|  |  |  | 22 | *PSMD9* | Isoform p27-L of 26S proteasome | 10 |
|  |  |  |  |  | non-ATPase regulatory subunit 9 (24.6kD, pI 7.0) |  |
|  |  |  | 7.7 | *BPGM* | Bisphosphoglycerate mutase (30kD, pI 6.5) | 3 |
| 20 | 15 | 55 / 6.5 | 20 | *WARS* | Tryptophanyl-tRNA synthetase, cytoplasmic (53kD, pI 6.2) | 17 |
| 43 | 16 | 53 / 6.1 | 26.3 | *CNDP2* | CNDP dipeptidase 2 (metallopeptidase M20 family)(52.9kD, pI 6.0) | 19 |
|  |  |  | 25.9 | *HARS* | Histidyl-tRNA synthetase, cytoplasmic (57.4kD, pI 6.9) | 23 |
|  |  |  | 5.1 | *FLAD1* | Isoform 1 of FAD synthetase (65kD, pI 6.9) | 2 |
| 31 | 17 | 25 / 6.2 | 19.3 | *RHOA* | ras homolog gene family, member A (22.2kD, pI 7.1) | 3 |
| 37 | 18 | 70 / 5.8 | 19.1 | *LCP1* | Lymphocyte cytosolic protein 1 (Plastin-2) (70.2kD, pI 5.3) | 24 |
| 80 | 19 | 30 / 7.0 | 31.6 | PSMA1 | Isoform Long of Proteasome subunit alpha type-1 (30.2kD, pI 7.0) | 9 |
| 89 | 20 | 55 / 6.2 | 25.2 | *HSPA8* | Isoform 1 of Heat shock cognate 71 kDa protein (70.8kD, pI 5.5) | 41 |
|  |  |  | 6.6 | *DARS* | Aspartyl-tRNA synthetase, cytoplasmic (57kD, pI 6.5) | 3 |
|  |  |  | 5.3 | *PEPD* | Xaa-Pro dipeptidase (peptidase D) (54.5kD, pI 6.0) | 2 |
| 218 | 21 | 75 / 6.2 | 32.2 | *GCLC* | Glutamate--cysteine ligase catalytic subunit (73kD, pI 6.1) | 25 |
|  |  |  | 15.8 | *PDE12* | 2',5'-Phosphodiesterase 12 (67kD, pI 6.6) | 9 |
|  |  |  | 13.4 | *SCFD1* | Sec1 family domain containing 1 (72.3kD, pI 6.3) | 5 |
| 8 | 22 | 35 / 5.0 | 17.2 | *ATG3* | ATG3 autophagy related 3 homolog (S. cerevisiae) (35.8kD, pI 4.7) | 10 |
| 32 | 23 | 60 / 5.5 | 22.8 | *APEH* | Acylamino-acid-releasing enzyme (81.2kD, pI 5.5) | 34 |
|  |  |  | 21.5 | *TUBA1B* | Tubulin alpha-1B chain (50.1kD, pI 5.1) | 16 |
|  |  |  |  | *(or B or C)* |  |  |
|  |  |  | 11 | *GDI1* | Rab GDP dissociation inhibitor alpha (50.5kD, pI 5.1) | 4 |
|  |  |  | 8.9 | *ATG3* | ATG3 autophagy related 3 homolog (S. cerevisiae) (35.8kD, pI 4.7) | 3 |
|  |  |  | 8.5 | *NAP1L4* | Nucleosome assembly protein 1-like 4 (43kD, pI 4.7) | 3 |
|  |  |  | 8.6 | *CASP8* | Isoform 9 of Caspase-8 precursor (61.8kD, pI 5.3) | 4 |
| 14 | 24 | 35 / 7.0 | 47.4 | *PNP* | Purin Nucleotide Phosphorylase (33kD, pI 7.2) | 59 |
| 55 | 25 | 60 / 5.5 | 23.3 | *USP14* | Ubiquitin carboxyl-terminal hydrolase 14 (56kD, pI 5.3) | 19 |
|  |  |  | 44 | *PNP* | Purin Nucleotide Phosphorylase (33kD, pI 7.2) carry over | 33 |
| 178 | 26 | 60 / 7.5 | 36 | *G6PD* | Isoform Long of Glucose-6-phosphate 1-dehydrogenase | 54 |
|  |  |  |  |  | (64kD, pI 6.9) |  |
|  |  |  | 6.5 | *PNP* | Purin Nucleotide Phosphorylase (33kD, pI 7.2) carry over | 2 |
| 301 | 27 | 47 / 8.0 | 30.2 | *PAFAH1B1* | Isoform 1 of Platelet-activ. factor acetylhydr. IB subunit Alpha | 14 |
|  |  |  |  |  | (46kD, pI 7.4) |  |
| 231 | 28 | 27 / 6.0 | 27.5 | PITHD1 | PITH domain-containing protein 1 (24.1kD, pI 5.8) | 27 |
|  |  |  | 9.1 | *PRDX1* | Peroxiredoxin-1 (22.1kD, pI 8.1) | 3 |
| 145 | 29 | 20 / 8.5 | 25.9 | *PSMB5* | Proteasome subunit, beta type, 5 (28kD, pI 6.5) | 52 |
| 116 | 30 | 35 / 5.6 | 19.8 | *MAPRE1* | Microtubule-associated protein, RP/EB family, member 1 | 6 |
|  |  |  |  |  | (29.9kD, pI 5.1) |  |
|  |  |  | 10.2 | *CCS* | Copper chaperone for superoxide dismutase (29kD, pI 5.6) | 2 |
| 17 | 31 | 37 / 5.6 | 28.9 | *TXNL1* | Thioredoxin-like 1 (36.7kD, pI 4.9) | 11 |
| 160 | 32 | 22 / 5.6 | 37.5 | *C1orf123* | Chromosome 1 open reading frame 123 (18kD, pI 5.0) | 6 |
| 148 | 33 | 25 / 5.8 | 24.3 | *APOA1BP* | Isoform 1 of Apolipoprotein A-I-binding protein precursor | 35 |
|  |  |  |  |  | (31.6kD, pI 7.7) |  |
|  |  |  | 35 | *C1orf123* | Chromosome 1 open reading frame 123 (18kD, pI 5.0) | 4 |
|  |  |  | 9.5 | *PITHD1* | PITH domain-containing protein 1 (24.1kD, 5.8) | 4 |
| 138 | 34 | 22 / 7.5 | 11.8 | *APOA1BP* | Isoform 1 of Apolipoprotein A-I-binding protein precursor | 2 |
|  |  |  |  |  | (31.6kD, pI 7.7) carry over |  |
| 96 | 35 | 60 / 8.1 | 26.3 | *CCT4* | T-complex protein 1 subunit delta (58kD, pI 7.8) | 65 |
|  |  |  | 12.3 | *CCT7* | T-complex protein 1 subunit eta (59.4kD, pI 7.6) | 9 |
| 136 | 36 | 33 / 5.8 | 31.2 | *CAPZB* | Capping protein (33.7kD, pI 6.4) | 24 |
|  |  |  | 5.4 | *CCT4* | T-complex protein 1 subunit delta (58kD, pI 7.8) carry over | 3 |
| 87 | 37 | 40 / 6.5 | 30.2 | *UROD* | Uroporphyrinogen decarboxylase (40.7kD, pI 6.1) | 6 |
| 3 | 38 | 30 / 6.5 | 29.4 | *PNP* | Purin Nucleotide Phosphorylase (33kD, pI 7.2) | 6 |
|  |  |  | 10.4 | *UROD* | Uroporphyrinogen decarboxylase (40.7kD, pI 6.1) | 3 |
| 15 | 39 | 25 / 5.0 | 20.7 | *TPT1* | Tumor protein, translationally-controlled 1 (21.5kD, pI 5.0) | 4 |
|  |  |  | 11.2 | *UROD* | Uroporphyrinogen decarboxylase (40.7kD, pI 6.1) | 3 |
| 33 | 40 | 50 / 6.2 | 31.9 | *GDI2* | Rab GDP dissociation inhibitor beta (50.6kD, pI 6.5) | 11 |
| 255 | 41 | 20 / 8.2 | 15.8 | *PSMB1* | Proteasome subunit beta type-1 precursor (26.5kD, pI 8.1) | 4 |
| 74 | 42 | 60 / 6.5 | 24.2 | *CCT3* | Chaperonin containing TCP1, subunit 3 (gamma) (60.5kD, pI 6.5) | 13 |
| 131 | 43 | 40 / 5.8 | 39.4 | *GLRX3* | Glutaredoxin-3 (37.4kD, pI 5.4) | 22 |
| 229 | 44 | 25 / 6.5 | 37.1 | *PSMB3* | Proteasome subunit beta type-3 (22.9kD, pI 6.5) | 22 |
| 124 | 45 | 60 / 6.5 | 27.5 | *TCP1* | T-complex protein 1 subunit alpha (60.3kD, pI 6.1) | 19 |
| 90 | 46 | 30 / 8.0 | 47.5 | *PSMA4* | Proteasome subunit alpha type-4 (29.4kD, pI 7.7) | 8 |
| 109 | 47 | 45 / 7.5 | 33.3 | *TSTA3* | GDP-L-fucose synthetase (35.8kD, pI 6.6) | 45 |
| 94 | 48 | 30 / 4.8 | *11.5* | *TSTA3* | *GDP-L-fucose synthetase (35.8kD,* pI 6.6*)* | 4 |
|  |  |  | 10 | *LOC440917* | Similar to 14-3-3 protein epsilon (29.6kD, pI 5.0) | 19 |

Spot rank: Rank of spot as assigned by Same Spots Software depending on fold change (normalized volume) comparing the highest to lowest sample

Pick #: Sequence in which spots were excised from gel depending on spot intensity (from lowest to highest)

Spot Location: Approximate location of spot by protein mass (marker on gel in kilo dalton, kD) and isoelectric point (pI) as estimated on focussing strip used

Coverage: Percentage of aminoacid sequence that is covered by unique peptides to identify protein

Gene: HGNC Symbol for coding human gene

Protein: HGNC Symbol for protein identified

SC: Spectral count
