# Supplementary material for: Assessment of the Red Cell Proteome of Young Patients with Unexplained Hemolytic Anemia by Two-Dimensional Differential In-Gel Electrophoresis (DIGE)
Source: PLoS One. 2012 Apr 3;7(4):e34237. doi: 10.1371/journal.pone.0034237 (PMC3317954; doi:10.1371/journal.pone.0034237)
Supplement: Table S11 — Complete list of all spots picked in HA19, fold change comparing patient to all other samples run in the experiment. (DOCX) [file pone.0034237.s015.docx]

# Table S11: Complete list of all spots picked in HA19, fold change comparing patient to all other samples run in the experiment

| Spot rank | Pick # | Fold change |  | Patient | Mother | Father | Control | Standart |
| --- | --- | --- | --- | --- | --- | --- | --- | --- |
| 6 | 1 |  | I | 3.091 | 0.550 | 0.560 | 0.854 | 0.529 |
|  |  |  | II | 3.295 | 0.528 | 0.547 | 0.892 | 0.514 |
|  |  |  | med | 3.193 | 0.539 | 0.554 | 0.873 | 0.522 |
|  | **P to all 5.14** | |  |  | **5.924** | **5.769** | **3.658** | **6.123** |
| 38 | 2 |  | I | 2.060 | 0.759 | 0.746 | 0.580 | 0.638 |
|  |  |  | II | 1.909 | 0.786 | 0.711 | 0.781 | 0.661 |
|  |  |  | med | 1.985 | 0.773 | 0.729 | 0.681 | 0.650 |
|  | **P to all 2.8** | |  |  | **2.569** | **2.724** | **2.916** | **3.055** |
| 22 | 3 |  | I | 2.930 | 0.784 | 0.750 | 0.756 | 0.903 |
|  |  |  | II | 2.908 | 0.812 | 0.889 | 0.811 | 0.850 |
|  |  |  | med | 2.919 | 0.798 | 0.820 | 0.784 | 0.877 |
|  | **P to all 3.56** | |  |  | **3.658** | **3.562** | **3.726** | **3.330** |
| 16 | 4 |  | I | 0.389 | 1.199 | 0.708 | 0.939 | 1.750 |
|  |  |  | II | 0.424 | 1.130 | 0.832 | 0.978 | 1.650 |
|  |  |  | med | 0.407 | 1.165 | 0.770 | 0.959 | 1.700 |
|  | *P to all 2.82* | |  |  | *2.86* | *1.89* | *2.36* | *4.18* |
| 4 | 5 |  | I | 3.112 | 0.569 | 0.641 | 0.489 | 0.705 |
|  |  |  | II | 3.091 | 0.584 | 0.600 | 0.517 | 0.587 |
|  |  |  | med | 3.102 | 0.577 | 0.621 | 0.503 | 0.646 |
|  | **P to all 5.3** | |  |  | **5.380** | **4.998** | **6.166** | **4.801** |
| 30 | 6 |  | I | 2.498 | 0.769 | 0.754 | 0.771 | 0.935 |
|  |  |  | II | 2.911 | 0.804 | 0.890 | 0.844 | 0.839 |
|  |  |  | med | 2.705 | 0.787 | 0.822 | 0.808 | 0.887 |
|  | **P to all 3.28** | |  |  | **3.439** | **3.290** | **3.349** | **3.049** |
| 54 | 7 |  | I | 0.436 | 1.010 | 0.754 | 0.918 | 1.216 |
|  |  |  | II | 0.413 | 0.901 | 0.808 | 0.946 | 1.117 |
|  |  |  | med | 0.425 | 0.956 | 0.781 | 0.932 | 1.167 |
|  | *P to all 2.26* | |  |  | *2.25* | *1.84* | *2.20* | *2.75* |
| 1 | 8 |  | I | 0.113 | 0.887 | 0.997 | 0.430 | 1.489 |
|  |  |  | II | 0.157 | 0.878 | 1.112 | 0.430 | 1.409 |
|  |  |  | med | 0.135 | 0.883 | 1.055 | 0.430 | 1.449 |
|  | *P to all 7.07* | |  |  | *6.54* | *7.81* | *3.19* | *10.73* |
| 9 | 9 |  | I | 0.249 | 1.144 | 1.234 | 1.372 | 1.015 |
|  |  |  | II | 0.282 | 1.206 | 1.135 | 1.328 | 0.884 |
|  |  |  | med | 0.266 | 1.175 | 1.185 | 1.350 | 0.950 |
|  | *P to all 4.39* | |  |  | *4.43* | *4.46* | *5.08* | *3.58* |
| 52 | 10 |  | I | 1.556 | 0.804 | 0.708 | 0.738 | 0.590 |
|  |  |  | II | 1.731 | 0.786 | 0.664 | 0.711 | 0.577 |
|  |  |  | med | 1.644 | 0.795 | 0.686 | 0.725 | 0.584 |
|  | **P to all 2.36** | |  |  | **2.067** | **2.396** | **2.268** | **2.817** |
| 48 | 11 |  | I | 1.885 | 0.632 | 0.623 | 0.938 | 0.724 |
|  |  |  | II | 2.065 | 0.734 | 0.753 | 0.795 | 0.666 |
|  |  |  |  | 1.975 | 0.683 | 0.688 | 0.867 | 0.695 |
|  | **P to all 2.69** | | **med** |  | **2.892** | **2.871** | **2.279** | **2.842** |
| 11 | 12 |  | I | 2.527 | 0.906 | 0.705 | 1.287 | 0.527 |
|  |  |  | II | 2.484 | 0.815 | 0.722 | 1.254 | 0.542 |
|  |  |  | med | 2.506 | 0.861 | 0.714 | 1.271 | 0.535 |
|  | **P to all 2.64** | |  |  | **2.912** | **3.512** | **1.972** | **4.688** |
| 28 | 13 |  | I | 0.309 | 0.994 | 1.197 | 1.316 | 0.964 |
|  |  |  | II | 0.407 | 1.081 | 1.100 | 1.437 | 0.843 |
|  |  |  | med | 0.358 | 1.038 | 1.149 | 1.377 | 0.904 |
|  | *P to all 3.12* | |  |  | *2.90* | *3.21* | *3.84* | *2.52* |
| 45 | 14 |  | I | 0.397 | 1.037 | 0.928 | 0.723 | 1.292 |
|  |  |  | II | 0.411 | 1.025 | 0.964 | 0.833 | 1.088 |
|  |  |  | med | 0.404 | 1.031 | 0.946 | 0.778 | 1.190 |
|  | *P to all 2.44* | |  |  | *2.55* | *2.34* | *1.93* | *2.95* |
| 20 | 15 |  | I | 2.200 | 0.522 | 0.571 | 0.768 | 0.590 |
|  |  |  | II | 2.039 | 0.556 | 0.596 | 0.822 | 0.618 |
|  |  |  | med | 2.120 | 0.539 | 0.584 | 0.795 | 0.604 |
|  | **P to all 3.36** | |  |  | **3.932** | **3.632** | **2.666** | **3.509** |
| 43 | 16 |  | I | 1.804 | 0.701 | 0.999 | 0.504 | 0.870 |
|  |  |  | II | 1.646 | 0.748 | 0.912 | 0.664 | 0.803 |
|  |  |  | med | 1.725 | 0.725 | 0.956 | 0.584 | 0.837 |
|  | **P to all 2.23** | |  |  | **2.381** | **1.805** | **2.954** | **2.062** |
| 31 | 17 |  | I | 0.557 | 1.882 | 2.041 | 1.524 | 2.214 |
|  |  |  | II | 0.685 | 2.041 | 2.018 | 1.691 | 1.956 |
|  |  |  | med | 0.621 | 1.962 | 2.030 | 1.608 | 2.085 |
|  | *P to all 3.09* | |  |  | *3.16* | *3.27* | *2.59* | *3.36* |
| 37 | 18 |  | I | 0.452 | 1.332 | 1.003 | 1.396 | 1.483 |
|  |  |  | II | 0.482 | 1.334 | 1.093 | 1.421 | 1.387 |
|  |  |  | med | 0.467 | 1.333 | 1.048 | 1.409 | 1.435 |
|  | *P to all 2.8* | |  |  | *2.85* | *2.24* | *3.02* | *3.07* |
| 80 | 19 |  | I | 0.457 | 1.245 | 0.903 | 0.882 | 1.191 |
|  |  |  | II | 0.480 | 1.038 | 1.019 | 0.822 | 1.043 |
|  |  |  |  | 0.469 | 1.142 | 0.961 | 0.852 | 1.117 |
|  | *P to all 2.17* | | *med* |  | *2.44* | *2.05* | *1.82* | *2.38* |
| 89 | 20 |  | I | 0.590 | 0.829 | 0.987 | 1.447 | 1.113 |
|  |  |  | II | 0.580 | 0.885 | 0.941 | 1.341 | 1.116 |
|  |  |  | med | 0.585 | 0.857 | 0.964 | 1.394 | 1.115 |
|  | *P to all 1.85* | |  |  | *1.46* | *1.65* | *2.38* | *1.91* |
| 218 | 21 |  | I | 1.524 | 0.966 | 0.898 | 1.008 | 1.025 |
|  |  |  | II | 1.638 | 0.974 | 0.906 | 1.036 | 0.991 |
|  |  |  | med | 1.581 | 0.970 | 0.902 | 1.022 | 1.008 |
|  | **P to all 1.62** | |  |  | **1.630** | **1.753** | **1.547** | **1.568** |
| 8 | 22 |  | I | 2.608 | 0.898 | 0.806 | 1.085 | *0.574* |
|  |  |  | II | 3.032 | 0.832 | 0.813 | 1.004 | *0.495* |
|  |  |  | med | 2.820 | 0.865 | 0.810 | 1.045 | 0.535 |
|  | **P to all 3.47** | |  |  | **3.260** | **3.484** | **2.700** | **5.276** |
| 32 | 23 |  | I | 1.952 | 0.660 | 0.534 | 0.666 | 0.763 |
|  |  |  | II | 1.662 | 0.771 | 0.571 | 0.767 | 0.688 |
|  |  |  | med | 1.807 | 0.716 | 0.553 | 0.717 | 0.726 |
|  | **P to all 2.67** | |  |  | **2.526** | **3.271** | **2.522** | **2.491** |
| 14 | 24 |  | I | 0.308 | 0.984 | 1.074 | 0.675 | 1.478 |
|  |  |  | II | 0.365 | 0.958 | 1.068 | 0.658 | 1.424 |
|  |  |  | med | 0.337 | 0.971 | 1.071 | 0.667 | 1.451 |
|  | *P to all 3.09* | |  |  | *2.89* | *3.18* | *1.98* | *4.31* |
| 55 | 25 |  | I | 0.386 | 1.236 | 1.017 | 0.883 | 1.125 |
|  |  |  | II | 0.444 | 1.029 | 1.211 | 0.787 | 0.975 |
|  |  |  | med | 0.415 | 1.133 | 1.114 | 0.835 | 1.050 |
|  | *P to all 2.49* | |  |  | *2.73* | *2.68* | *2.01* | *2.53* |
| 178 | 26 |  | I | 1.427 | 0.717 | 0.830 | 0.817 | 0.733 |
|  |  |  | II | 1.285 | 0.753 | 0.785 | 0.919 | 0.771 |
|  |  |  | med | 1.356 | 0.735 | 0.808 | 0.868 | 0.752 |
|  | **P to all 1.72** | |  |  | **1.845** | **1.679** | **1.562** | **1.803** |
| 301 | 27 |  | I | 0.665 | 0.776 | 0.880 | 0.979 | 0.980 |
|  |  |  | II | 0.660 | 0.763 | 0.860 | 0.987 | 0.960 |
|  |  |  | med | 0.663 | 0.770 | 0.870 | 0.983 | 0.970 |
|  | P to all 1.36 | |  |  | 1.16 | 1.31 | 1.48 | 1.46 |
| 231 | 28 |  | I | 1.242 | 0.960 | 0.743 | 0.723 | 0.731 |
|  |  |  | II | 1.166 | 0.916 | 0.780 | 0.679 | 0.741 |
|  |  |  | med | 1.204 | 0.938 | 0.762 | 0.701 | 0.736 |
|  | **P to all 1.54** | |  |  | **1.284** | **1.581** | **1.718** | **1.636** |
| 145 | 29 |  | I | 0.617 | 1.105 | 0.996 | 0.943 | 1.250 |
|  |  |  | II | 0.615 | 1.006 | 1.007 | 0.961 | 1.187 |
|  |  |  | med | 0.616 | 1.056 | 1.002 | 0.952 | 1.219 |
|  | *P to all 1.72* | |  |  | *1.71* | *1.63* | *1.55* | *1.98* |
| 116 | 30 |  | I | 1.495 | 0.956 | 0.689 | 1.141 | 0.781 |
|  |  |  | II | 1.368 | 0.928 | 0.653 | 1.178 | 0.843 |
|  |  |  | med | 1.432 | 0.942 | 0.671 | 1.160 | 0.812 |
|  | **P to all 1.6** | |  |  | **1.520** | **2.133** | **1.235** | **1.763** |
| 17 | 31 |  | I | 1.899 | 0.734 | 0.831 | *0.457* | 0.721 |
|  |  |  | II | 1.782 | 0.733 | 0.859 | *0.440* | 0.697 |
|  |  |  | med | 1.841 | 0.734 | 0.845 | 0.449 | 0.709 |
|  | **P to all 2.69** | |  |  | **2.509** | **2.178** | **4.104** | **2.596** |
| 160 | 32 |  | I | 0.541 | 0.999 | 0.941 | 0.848 | 1.012 |
|  |  |  | II | 0.523 | 1.043 | 0.949 | 0.885 | 0.962 |
|  |  |  | med | 0.532 | 1.021 | 0.945 | 0.867 | 0.987 |
|  | *P to all 1.79* | |  |  | *1.92* | *1.78* | *1.63* | *1.86* |
| 148 | 33 |  | I | 0.720 | 1.298 | 1.356 | *0.852* | 1.255 |
|  |  |  | II | 0.645 | 1.382 | 1.326 | *0.932* | 1.423 |
|  |  |  | med | 0.683 | 1.340 | 1.341 | 0.892 | 1.339 |
|  | *P to all 1.8* | |  |  | *1.96* | *1.96* | *1.31* | *1.96* |
| 138 | 34 |  | I | 0.666 | 1.016 | 1.109 | 0.984 | 1.343 |
|  |  |  | II | 0.686 | 1.022 | 1.201 | 1.019 | 1.375 |
|  |  |  | med | 0.676 | 1.019 | 1.155 | 1.002 | 1.359 |
|  | *P to all 1.68* | |  |  | *1.51* | *1.71* | *1.48* | *2.01* |
| 96 | 35 |  | I | 1.529 | 0.656 | 0.697 | *1.161* | 0.812 |
|  |  |  | II | 1.678 | 0.747 | 0.737 | *1.276* | 0.754 |
|  |  |  | med | 1.604 | 0.702 | 0.717 | 1.219 | 0.783 |
|  | **P to all 1.88** | |  |  | **2.286** | **2.236** | **1.316** | **2.048** |
| 136 | 36 |  | I | 0.582 | 1.140 | 1.017 | 0.784 | 1.166 |
|  |  |  | II | 0.535 | 1.038 | 1.230 | 0.744 | 1.095 |
|  |  |  | med | 0.559 | 1.089 | 1.124 | 0.764 | 1.131 |
|  | *P to all 1.84* | |  |  | *1.95* | *2.01* | *1.37* | *2.02* |
| 87 | 37 |  | I | 1.658 | 0.712 | 0.741 | 0.707 | 0.813 |
|  |  |  | II | 1.620 | 0.667 | 0.805 | 0.719 | 0.652 |
|  |  |  | med | 1.639 | 0.690 | 0.773 | 0.713 | 0.733 |
|  | **P to all 2.25** | |  |  | **2.377** | **2.120** | **2.299** | **2.238** |
| 3 | 38 |  | I | 0.170 | 0.992 | 0.994 | 0.519 | 1.569 |
|  |  |  | II | 0.169 | 0.926 | 1.031 | 0.523 | 1.347 |
|  |  |  | med | 0.170 | 0.959 | 1.013 | 0.521 | 1.458 |
|  | *P to all 5.85* | |  |  | *5.66* | *5.97* | *3.07* | *8.60* |
| 15 | 39 |  | I | 2.015 | 0.683 | 0.502 | 1.086 | 0.752 |
|  |  |  | II | 2.396 | 0.549 | 0.569 | 1.034 | 0.626 |
|  |  |  | med | 2.206 | 0.616 | 0.536 | 1.060 | 0.689 |
|  | **P to all 3.04** | |  |  | **3.580** | **4.119** | **2.081** | **3.201** |
| 33 | 40 |  | I | 0.386 | 1.101 | 1.087 | 0.920 | 1.339 |
|  |  |  | II | 0.453 | 1.194 | 1.103 | 0.799 | 1.348 |
|  |  |  | med | 0.420 | 1.148 | 1.095 | 0.860 | 1.344 |
|  | *P to all 2.65* | |  |  | *2.74* | *2.61* | *2.05* | *3.20* |
| 255 | 41 |  | I | 0.768 | 1.142 | 1.075 | 0.984 | 1.194 |
|  |  |  | II | 0.700 | 1.085 | 1.089 | 1.032 | 1.192 |
|  |  |  | med | 0.734 | 1.114 | 1.082 | 1.008 | 1.193 |
|  | *P to all 1.5* | |  |  | *1.52* | *1.47* | *1.37* | *1.63* |
| 74 | 42 |  | I | 1.582 | 0.737 | 0.771 | 1.129 | 0.640 |
|  |  |  | II | 1.576 | 0.737 | 0.797 | 1.147 | 0.630 |
|  |  |  | med | 1.579 | 0.737 | 0.784 | 1.138 | 0.635 |
|  | **P to all 1.92** | |  |  | **2.142** | **2.014** | **1.388** | **2.487** |
| 131 | 43 |  | I | 1.511 | 0.899 | 0.878 | 0.856 | 0.747 |
|  |  |  | II | 1.455 | 0.889 | 0.875 | 0.934 | 0.712 |
|  |  |  | med | 1.483 | 0.894 | 0.877 | 0.895 | 0.730 |
|  | **P to all 1.75** | |  |  | **1.659** | **1.692** | **1.657** | **2.033** |
| 229 | 44 |  | I | 0.698 | 1.093 | 0.967 | 0.947 | 1.257 |
|  |  |  | II | 0.690 | 0.985 | 1.109 | 0.901 | 1.150 |
|  |  |  | med | 0.694 | 1.039 | 1.038 | 0.924 | 1.204 |
|  | *P to all 1.51* | |  |  | *1.50* | *1.50* | *1.33* | *1.73* |
| 124 | 45 |  | I | 1.529 | 0.772 | 0.720 | *1.220* | 0.815 |
|  |  |  | II | 1.603 | 0.745 | 0.772 | *1.515* | 0.729 |
|  |  |  | med | 1.566 | 0.759 | 0.746 | 1.368 | 0.772 |
|  | **P to all 1.72** | |  |  | **2.065** | **2.099** | **1.145** | **2.028** |
| 90 | 46 |  | I | 0.473 | 1.194 | 0.986 | 0.887 | 1.096 |
|  |  |  | II | 0.523 | 1.108 | 1.008 | 0.888 | 1.162 |
|  |  |  | med | 0.498 | 1.151 | 0.997 | 0.888 | 1.129 |
|  | *P to all 2.09* | |  |  | *2.31* | *2.00* | *1.78* | *2.27* |
| 109 | 47 |  | I | 1.471 | 0.904 | 0.877 | 0.842 | 0.687 |
|  |  |  | II | 1.466 | 0.857 | 0.948 | 0.792 | 0.659 |
|  |  |  | med | 1.469 | 0.881 | 0.913 | 0.817 | 0.673 |
|  | **P to all 1.79** | |  |  | **1.668** | **1.609** | **1.797** | **2.182** |
| 94 | 48 |  | I | 0.645 | 0.711 | 0.906 | 0.978 | 1.457 |
|  |  |  | II | 0.643 | 0.677 | 0.904 | 0.987 | 1.530 |
|  |  |  | med | 0.644 | 0.694 | 0.905 | 0.983 | 1.494 |
|  | *P to all 1.59* | |  |  | *1.08* | *1.41* | *1.53* | *2.32* |

Spot rank: Rank of spot as assigned by Same Spots Software depending on fold change (normalized volume) comparing the highest to lowest sample

Pick #: Sequence in which spots were excised from gel depending on spot intensity (from lowest to highest)

Roman Numerals: Normalized volume measured in replicate runs one (I) and two (II)

Med: Average of normalized volume measured in replicate runs one (I) and two (II)

Fold Change P to all: Expression level (normalized volume) of patient sample compared to the average of all other samples combined;

Last row of columns also shows fold change comparing patient to sample indicated in header of column (Mother, Father, Control, Standard)

Formatting: **Bold**: Patient upregulated *Italic*: Patient downregulated

No special formatting: Fold change “P to all” does not exceed 1.5 fold
