# Supplementary material for: Assessment of the Red Cell Proteome of Young Patients with Unexplained Hemolytic Anemia by Two-Dimensional Differential In-Gel Electrophoresis (DIGE)
Source: PLoS One. 2012 Apr 3;7(4):e34237. doi: 10.1371/journal.pone.0034237 (PMC3317954; doi:10.1371/journal.pone.0034237)
Supplement: Table S12 — Complete list of all spots picked and Proteins identified in HA21. (DOCX) [file pone.0034237.s016.docx]

# Table S12: Complete list of all spots picked and Proteins identified in HA21

| **Spot rank** | **Pick #** | **Spot Location**  **(kD / pI)** | **Coverage**  **(%)** | Gene | **Protein** | | SC |
| --- | --- | --- | --- | --- | --- | --- | --- |
| 20 | 1 | 90 / 7.5 | 14.1 | *RRM1* | Ribonucleosidee-diphosphate reductase large subunit | | 11 |
|  |  |  |  |  | (90kD, pI 7.1) | |  |
|  |  |  | 7.5 | *HSP90AA1* | Heat shock protein 90kDa alpha (cytosolic), | | 5 |
|  |  |  |  |  | class A member 1 isoform 1 (98kD, pI 5.2) | |  |
| 5 | 2 | 100 / 7.2 | 10.7 | *EEF2* | Elongation factor 2 (95kD, pI 6.8) | | 9 |
| 29 | 3 | 25 / 8.5 |  |  | NO ID | |  |
| 7 | 4 | 90 / 7.5 | 7.8 | *RRM1* | Ribonucleosidee-diphosphate reductase large subunit | | 5 |
|  |  |  |  |  | (90kD. pI 7.1) | |  |
| 232 | 5 | 100 / 7.2 | 1.2 | *COL3A1* | Isoform 1 of Collagen alpha-1(III) chain precursor (136kD, pI 6.6) | | 2 |
| 133 | 6 | 95 / 7.8 | 11.5 | *CUL4A* | Cullin-4A (88kD, pI 8.1) | | 10 |
|  |  |  | 9.8 | *CUL5* | Cullin-5 (96.7kD, pI 8.4) | | 10 |
|  |  |  | 7.6 | *CAPN1* | Calpain-1 catalytic subunit (81.8k, pI 5.7) | | 3 |
| 21 | 7 | 15 / 6.5 | 10.1 | *BTF3L4* | Transcription factor BTF3 homolog 4 (17.2kD, pI 6.4) | | 3 |
| 3 | 8 | 100 / 7.5 | 7 | *EEF2* | Elongation factor 2 (95kD, pI 6.8) | | 7 |
| 298 | 9 | 80 / 7.0 | 17.3 | *CTPS* | CTP synthase 1 (66.7kD, pI 6.5) | | 15 |
|  |  |  | 5.1 | *ASPSCR1* | Isoform 2 of Tether containing UBX domain for GLUT4 | | 2 |
|  |  |  |  |  | (69.9kD, pI 8.1) | |  |
|  |  |  | 4.6 | *GCLC* | Glutamate--cysteine ligase catalytic subunit (72.7kD, 6.1) | | 2 |
| 98 | 10 | 110 / 6.5 | 9.2 | *ALAD* | Delta-aminolevulinic acid dehydratase isoform a (39kD, 7.6) | | 3 |
|  |  |  | 3.6 | *XPO7* | Exportin 7 isoform a (125kD, pI 6.3) | | 3 |
| 151 | 11 | 50 / 7.2 | 14.9 | *FH* | Fumarate hydratase, mitochondrial, precursor (54.6kD, pI 8.8) | | 8 |
|  |  |  | 5.6 | PAFAH1B1 | Isoform 1 of Platelet-activating factor acetylhydrolase IB | | 4 |
|  |  |  |  |  | subunit alpha (46.6kD, pI 7.4) | |  |
| 108 | 12 | 50 / 7.8 | 25.2 | *PSMC6* | 26S protease regulatory subunit S10B (44kD, pI 7.5) | | 10 |
|  |  |  | 18.9 | *CSNK2A1* | Casein kinase 2, alpha 1 polypeptide (45.9kD, pI 7.9) | | 12 |
| 27 | 13 | 60 / 5.0 | 55.3 | *TUBB2C* | Tubulin beta-2C chain (49.8kD, pI 4.9) | | 56 |
|  |  |  | 52 | *TUBB* | Tubulin beta chain (49.6kD, pI 4.9) | | 104 |
|  |  |  | 49.5 | *TUBB4* | Tubulin beta-4 chain (49.5kD, pI 4.9) | | 51 |
|  |  |  | 45.2 | *TUBB2B* | Tubulin beta-2B chain (49.9kD, pI 4.9) | | 39 |
|  |  |  | 30.4 | *TUBB6* | Tubulin, beta 6 (50kD, pI 4.9) | | 31 |
|  |  |  | 14.9 | *TUBB3* | Tubulin, beta, 4 (88.4kD, pI 5.9) | | 32 |
|  |  |  | 6.6 | *CRLF3* | Cytokine receptor-like factor 3 (49.7kD, pI 5.1) | | 4 |
| 258 | 14 | 30 / 6.4 | 10.9 | *HDHD2* | Haloacid dehalogenase-like hydrolase domain containing 2 | | 4 |
|  |  |  |  |  | (29.6kD, pI 6.4) | |  |
| 87 | 15 | 29 / 6.7 | 52 | *PNP* | Purine Nucleoside Phosphorylase (33kD, pI 7.2) | | 24 |
| 45 | 16 | 55 / 6.3 | 14.7 | *BLMH* | Bleomycin hydrolase (52.5 kD, pI 6.3) | | 10 |
|  |  |  | 8.5 | *GDI2* | Rab GDP dissociation inhibitor beta (50.6kD, pI 6.5) | | 3 |
|  |  |  | 6.3 | *GSS* | Glutathione synthetase (52.3kD, pI 5.9) | | 3 |
|  |  |  | 5.5 | *ARHGAP1* | Rho GTPase-activating protein 1 (50.4kD, pI 6.3) | | 4 |
| 389 | 17 | 125 / 6.5 | 18.9 | *XPO7* | Exportin 7 isoform a (124kD, pI 6.3) | | 25 |
|  |  |  | 7.5 | *IDE* | Insulin-degrading enzyme (118kD, pI 6.8) | | 9 |
|  |  |  | 5.9 | *BLMH* | Bleomycin hydrolase (52.5 kD, pI 6.3) carry over | | 3 |
| 12 | 18 | 60 / 6.8 | 15.3 | *FBXO7* | F-box only protein 7 (58kD, pI 6.8) | | 25 |
|  |  |  | 9.8 | *G6PD* | Isoform Long of Glucose-6-phosphate 1-dehydrogenase | | 4 |
|  |  |  |  |  | (64kD, pI 6.9) | |  |
|  |  |  | 4.7 | *GPS1* | Isoform 3 of COP9 signalosome complex subunit 1(60.4kD, pI 6.9) | | 3 |
| 48 | 19 | 65 / 6.9 | 26.7 | *CCT6A* | T-complex protein 1 subunit zeta 1 (58kD, pI 6.7) | | 16 |
|  |  |  | 14.4 | *STIP1* | Stress-induced-phosphoprotein 1 (68kD, pI 7.7) | | 14 |
|  |  |  | 13.7 | *ATIC* | Bifunctional purine biosynthesis protein PURH (64.6kD, pI 6.7): | | 6 |
|  |  |  | 6.9 | *COASY* | Bifunctional coenzyme A synthase (62.3kD, pI 7.0) | | 4 |
|  |  |  | 4.7 | *GPS1* | Isoform 3 of COP9 signalosome complex subunit 1(60.4kD, pI 6.9) | | 3 |
|  |  |  |  |  | carry over | |  |
| 103 | 20 | 40 / 7.5 | 14.3 | *HDHD2* | Haloacid dehalogenase-like hydrolase domain containing 2 | | 11 |
|  |  |  |  |  | (29.6kD, 6.4) | |  |
|  |  |  | 24 | *GMPR2* | Guanosine monophosphate reductase 2 (45kD, pI 8.3) | | 11 |
| 6 | 21 | 45 / 4.8 | 37.3 | *LOC387867* | Ribosomal protein SA pseudogene 12 (32.7kD, pI 4.5) | | 28 |
|  |  |  | 33.7 | *RPSA* | Ribosomal protein SA (33.3kD, pI 4.9) | | 30 |
| 116 | 22 | 50 / 6.4 | 10.8 | *RPSA* | Ribosomal protein SA (33.3kD, pI 4.9) carry over | | 3 |
|  |  |  | 10.6 | *GDI2* | Rab GDP dissociation inhibitor beta (50.6kD, pI 6.5) | | 4 |
|  |  |  | 8.6 | *BLMH* | Bleomycin hydrolase (52.5 kD, pI 6.3) | | 4 |
| 188 | 23 | 27 / 5.5 | 20 | *GSTM3* | Glutathione S-transferase mu 3 (brain) (26.5kD, pI 5.5) | | 9 |
|  |  |  | 16.7 | *APOA1BP* | Isoform 1 of Apolipoprotein A-I-binding protein precursor | | 9 |
|  |  |  |  |  | (31.6kD, pI 7.7) | |  |
|  |  |  | 9.5 | PITHD1 | PITH domain-containing protein 1 (24kD, pI 5.8) | | 3 |
| 61 | 24 | 27 / 6.2 | 21.6 | *PSMB4* | Proteasome subunit beta type-4 precursor (29.2kD, pI 6.0) | | 14 |
| 51 | 25 | 35 / 5.5 | 18.6 | *NAPA* | Alpha-soluble NSF attachment protein (33.2kD, pI 5.4) | | 9 |
|  |  |  | 10.4 | *APOA1BP* | Isoform 1 of Apolipoprotein A-I-binding protein precursor | | 4 |
|  |  |  |  |  | (31.6kD, pI 7.7) | |  |
|  |  |  | 7.9 | *NAPRT1* | Nicotinate phosphoribosyltransferase domain-containing protein 1 | | 9 |
|  |  |  |  |  | (60.2kD, pI 5.8) | |  |
| 204 | 26 | 15 / 6.2 | 28.2 | *SOD1* | Superoxide dismutase 1, soluble (16.1kD, pI 6.3) | | 5 |
| 304 | 27 | 60 / 6.8 | 43.7 | *G6PD* | Isoform Long of Glucose-6-phosphate 1-dehydrogenase | | 54 |
|  |  |  |  |  | (64kD, pI 6.9) | |  |
|  |  |  | 5.6 | *DARS* | Aspartyl-tRNA synthetase, cytoplasmic (57kD, pI 6.5) | | 2 |
| 127 | 28 | 30 / 6.4 | 31.7 | *HPRT1* | Hypoxanthine phosphoribosyltransferase 1 (24.6kD, pI 7.0) | | 9 |
|  |  |  | 28.5 | *TSN* | Translin (26.1kD, pI 6.4) | | 20 |
| 72 | 29 | 50 / 7.2 | 20 | *FH* | Fumarate hydratase, mitochondrial, precursor (54.6kD, pI 8.8) | | 12 |
| 25 | 30 | 40 / 4.9 | 10.2 | *ATG3* | Isoform 1 of Autophagy-related protein 3 (35.9kD, pI 4.7) | | 3 |
| 158 | 31 | 27 / 6.1 | 44.1 | *PITHD1* | PITH domain-containing protein 1 (24kD, pI 5.8) | | 19 |
|  |  |  | 12.3 | *TSN* | Translin (26.1kD, pI 6.4) | | 2 |
| 67 | 32 | 45 / 6.8 | 33 | *ACTR1A* | Alpha-centractin (42kD, pI 6.6) | | 39 |
| 559 | 33 | 55 / 7.1 | 43.4 | *ADSL* | Isoform 1 of Adenylosuccinate lyase (55kD, pI 7.1) | | 29 |
|  |  |  | 10.1 | *ACTR1A* | Alpha-centractin (42kD, pI 6.6) carry over | | 3 |
| 291 | 34 | 28 / 7.0 | 30.7 | *QDPR* | Dihydropteridine reductase (25.7kD, pI 7.4) | | 8 |
|  |  |  | 15.5 | *ADSL* | Isoform 1 of Adenylosuccinate lyase (55kD, pI 7.1) carry over | | 7 |
|  |  |  | 10.1 | *ACTR1A* | Alpha-centractin (42kD, 6.6) carry over | | 2 |
|  |  |  | 4.6 | *NAPRT1* | Nicotinate phosphoribosyltransferase domain-cont.protein 1 | | 2 |
|  |  |  |  |  | (60.2kD, pI 5.8) | |  |
| 143 | 35 | 70 / 5.5 | 38.3 | *LCP1* | Lymphocyte cytosolic protein 1 (L-plastin) Plastin-2 | | 53 |
|  |  |  |  |  | (70.2kD, pI 5.3) | |  |
| 150 | 36 | 55 / 6.3 | 14.4 | *ARHGAP1* | Rho GTPase-activating protein 1 (50.4kD, pI 6.3) | | 7 |
|  |  |  | 10.9 | *MAT2A* | S-adenosylmethionine synthetase isoform type-2 (43.6kD, pI 6.5) | | 6 |
|  |  |  | 5.7 | *SH3GLB2* | Isoform 2 of SH3 domain GRB2-like protein B2 endophilin B2 | | 2 |
|  |  |  |  |  | (44.7kD, pI 5.6) | |  |
| 32 | 37 | 35 / 6.5 | 22.9 | *PSMD14* | 26S proteasome non-ATPase regulatory subunit 14 | | 7 |
|  |  |  |  |  | (34.5kD,pI 6.5) | |  |
|  |  |  | 22 | *ALAD* | Delta-aminolevulinic acid dehydratase isoform a (39kD, pI 7.6) | | 23 |
| 47 | 38 | 60 / 6.4 | 17.7 | *TXNRD1* | Thioredoxin reductase 1 isoform 3 (71.1kD, pI 7.5) | | 11 |
|  |  |  | 12.1 | *OXSR1* | Serine/threonine-protein kinase OSR1 (58kD, pI 6.4) | | 10 |
|  |  |  | 10.2 | *FBXO7* | F-box only protein 7 (58kD, pI 6.8) | | 9 |
| 544 | 39 | 26 / 7.1 | 42.5 | *PGAM1* | Phosphoglycerate mutase 1 (brain) (28.8kD, pI 7.2) | | 45 |
|  |  |  | 20.6 | *PSMA7* | Isoform 1 of Proteasome subunit alpha type-7 (28kD, pI 8.5) | | 4 |
|  |  |  | 11.1 | *PSMD8* | Proteasome 26S non-ATPase subunit 8 (39.6kD, pI 9.7) | | 4 |
| 77 | 40 | 16 / 6.0 | 12.8 | *SOD1* | Superoxide dismutase 1, soluble (16.1, pI 6.3) | | 4 |
|  |  |  | 9.1 | *PRDX2* | Peroxiredoxin-2 (21.8kD, pI 6.0) | | 2 |
| 8 | 41 | 33 / 6.2 | 60.4 | *PNP* | Purine Nucleoside Phosphorylase (33kD, pI 7.2) | | 40 |
| 86 | 42 | 35 / 5.0 | 50.2 | *TXNL1* | Thioredoxin-like 1(36.7kD, pI 4.9) | | 59 |
|  |  |  | 26.3 | *PNP* | Purine Nucleoside Phosphorylase (33kD, pI 7.2) | | 9 |
|  |  |  | 0.8 | *FGD1* | RhoGEF and PH domain-containing protein 1 (106kD, pI 6.6) | | 2 |
| 69 | 43 | 75 / 6.2 | 17.6 | *GCLC* | Glutamate--cysteine ligase catalytic subunit (73kD, pI 6.1) | | 15 |
|  |  |  | 8.5 | *ALB* | Uncharacterized protein ALB (71kD, pI 6.7) | | 5 |
|  |  |  | 7.3 | *SCFD1* | Sec1 family domain-containing protein 1 (72.3kD, pI 6.3) | | 5 |
|  |  |  | 4.3 | *PDE12* | 2',5'-Phosphodiesterase 12 (67.3kD, pI 6.6) | | 3 |
| 128 | 44 | 30 / 5.9 | 25.2 | *CAPZB* | Capping protein (33.7kD, pI 6.4) | | 12 |
| 28 | 45 | 55 / 8.2 | 26.9 | *EIF2S3* | Eukaryotic translation initiation factor 2 subunit 3 (51.1kD, pI 8.4) | | 26 |
| 489 | 46 | 30 / 7.9 | 24.8 | *ATP6V1E1* | Vacuolar ATP synthase subunit E 1 (26.1kD, pI 8.0) | | 19 |
| 19 | 47 | 32 / 7.2 | 66.2 | *PNP* | Purine Nucleoside Phosphorylase (33kD, pI 7.2) | | 48 |
|  |  |  | 15 | *ATP6V1E1* | Vacuolar ATP synthase subunit E 1 (26.1kD, pI 8.0) carry over | | 3 |
| 124 | 48 | 26 / 6.2 | 30.2 | *PSMB3* | Proteasome subunit beta type-3 (22.9kD, pI 6.5) | | 9 |
|  |  |  | 31 | *PSMD10* | 26S proteasome non-ATPase regulatory subunit 10 (24kD, pI 6.1) | | 8 |
|  |  |  | 25.5 | *RHOC* | Ras homolog gene family, member C (22kD, pI 7.6) | | 7 |
|  |  |  | 25.4 | *RHOA* | Ras homolog gene family, member A (22.2kD, pI 7.1) | | 13 |
|  |  |  | 25 | *PNP* | Purine Nucleoside Phosphorylase (33kD, pI 7.2) | | 11 |
| 173 | 49 | 40 / 6.7 | 43 | *TSTA3* | GDP-L-fucose synthetase (35.8kD, pI 6.6) | | 41 |
|  |  |  | 12.7 | *PRPSAP1* | Phosphoribosyl pyrophosphate synthetase-associated protein 1 | | 5 |
|  |  |  |  |  | (42kD, pI 8.5) | |  |
|  |  |  | 7.3 | *PRPSAP2* | Phosphoribosyl pyrophosphate synthetase-associated protein 1 | | 3 |
|  |  |  |  |  | (42.4kD, pI 8.5) | |  |
|  |  |  | 7.1 | *ACOT7* | Isoform 1 of Cytosolic acyl coenzyme A thioester hydrolase | | 2 |
|  |  |  |  |  | (41.7kD, pI 8.5) | |  |
| 174 | 50 | 30 / 6.9 | 34.1 | *PSMA4* | Proteasome subunit alpha type-4 (29.4kD, pI 7.7) | | 20 |
|  |  |  | 22 | *PSMD9* | Isoform p27-L of 26S proteasome non-ATPase regulatory SU 9 | | 4 |
|  |  |  |  |  | (24.6kD, pI 7.0) | |  |
|  |  |  | 16 | *COPS7A* | COP9 signalosome complex subunit 7a (30.2kD, pI 8.2) | | 4 |
|  |  |  | 8.1 | *TSTA3* | GDP-L-fucose synthetase (35.8kD, pI 6.6) | | 3 |
| 163 | 51 | 75 / 6.2 | 39.7 | *GCLC* | Glutamate--cysteine ligase catalytic subunit (73kD, pI 6.1) | | 43 |
|  |  |  | 15.6 | *SCFD1* | Sec1 family domain-containing protein 1 (72.3kD, pI 6.3) | | 6 |
|  |  |  | 12.8 | *XPNPEP1* | Xaa-Pro aminopeptidase 1 (74.9kD, pI 5.9) | | 11 |
|  |  |  | 6.3 | *GSS* | Glutathione synthetase (52.3kD, pI 5.9) | | 4 |
|  |  |  | 5.8 | *TXNRD1* | Thioredoxin reductase 1 isoform 3 (71.1kD, pI 7.5) | | 3 |
|  |  |  | 4.5 | *UBA1* | Ubiquitin-activating enzyme E1 (117kD, pI 5.8) | | 4 |
| 62 | 52 | 40 / 6.3 | 37.6 | *UROD* | Uroporphyrinogen decarboxylase (40.7kD, pI 6.1) | | 37 |
|  |  |  | 14.1 | *MGC3207 / MRI1* | Hypothetical protein LOC84245 isoform 1 (39.1kD, pI 6.3) | | 5 |
|  |  |  |  |  | Methylthioribose-1-phosphate isomerase homolog (S. cerevisiae) | |  |
|  |  |  | 7.2 | *NIF3L1* | Putative uncharacterized protein NIF3L1 (42kD, pI 6.7) | | 4 |
| 169 | 53 | 55 / 5.2 | 53.2 | *TUBA1B* | Tubulin alpha-1B chain (50.1kD, pI 5.1) | | 56 |
|  |  |  | 46 | *TUBA4A* | Tubulin alpha-4A chain (49.9kD, pI 5.1) | | 41 |
|  |  |  | 16.3 | *APEH* | Acylamino-acid-releasing enzyme (81.2kD, pI 5.5) | | 9 |
|  |  |  | 14.6 | *TUBB2C* | Tubulin beta-2C chain (49.8kD, pI 5.9) | | 5 |
|  |  |  | 10.6 | *UROD* | Uroporphyrinogen decarboxylase (40.7kD, pI 6.1) | | 2 |
| 113 | 54 | 37 / 5.4 | 33.7 | *ALAD* | Delta-aminolevulinic acid dehydratase isoform a (39kD, pI 7.6) | | 28 |
|  |  |  | 10.2 | *LDHB* | L-lactate dehydrogenase B chain (36.6kD, pI 6.1) | | 4 |
|  |  |  | 10.1 | *C1orf80* | Hypothetical protein LOC64853 (35kD, pI 6.6) | | 3 |
| 229 | 55 | 30 / 6.5 | 39.2 | *DHRS11* | Isoform 1 of Dehydrogenase/reductase SDR family member 11 | | 15 |
|  |  |  |  |  | precursor (28.3kD, pI 6.6) | |  |
|  |  |  | 24.9 | *HSPB1* | Heat shock protein beta-1 (22.7kD, pI 6.4) | | 9 |
|  |  |  | 23.2 | *PSMA6* | Proteasome subunit alpha type-6 (27.4kD, pI 6.7) | | 16 |
|  |  |  | 9.5 | *ALAD* | Delta-aminolevulinic acid dehydratase isoform a (39kD, pI 7.6) | | 4 |
| 162 | 56 | 110 / 5.5 | 36.5 | *VCP* | Valosin-containing protein (89.3kD, pI 5.2) | | 59 |
|  |  |  | 9.7 | *PSMD2* | 26S proteasome non-ATPase regulatory subunit 2 (100kD, pI 5.2) | | 7 |
|  |  |  | 6.4 | *TGM2* | Isoform 1 of Protein-glutamine gamma-glutamyltransferase 2 | | 5 |
|  |  |  |  |  | (77.3kD, pI 5.2) | |  |
|  |  |  | 3.4 | *KPNB1* | Karyopherin Importin subunit beta-1 (97kD, pI 4.8) | | 4 |
| 56 | 57 | 55 / 7.5 | 29 | *PSMD12* | 26S proteasome non-ATPase regulatory SU 12 (52.9kD, pI 7.6) | | 26 |
| 22 | 58 | 50 / 6.4 | 56.6 | *GDI2* | Rab GDP dissociation inhibitor beta (50.6kD, pI 6.5) | | 47 |
|  |  |  | 15 | *GDI1* | Rab GDP dissociation inhibitor alpha (50.5kD, pI 5.1) | | 7 |
|  |  |  | 12.2 | *PSMC2* | 26S protease regulatory subunit 7 (58.6kD, pI 5.9) | | 3 |
|  |  |  | 6.5 | *ST13* | Hsc70-interacting protein (41.3kD, pI 5.3) | | 2 |
| 178 | 59 | 30 / 6.6 | 36.4 | *PSMA1* | Isoform Long of Proteasome subunit alpha type-1 (30.2kD, pI 7.0) | | 18 |
|  |  |  | 28.9 | *ASNA1* | ATPase ASNA1 (40.2kD, pI 5.1 | | 9 |
| 90 | 60 | 40 / 4.9 | 42.7 | *ASNA1* | ATPase ASNA1 (40.2kD, pI 5.1) carry over | | 25 |
|  |  |  | 18 | *SMS* | Spermine synthase (41.2kD, pI 5.0) | | 6 |
|  |  |  | 10.1 | *SUGT1* | Isoform 1 of Suppressor of G2 allele of SKP1 homolog | | 2 |
|  |  |  |  |  | (41.2kD, pI 5.2) | |  |
| 223 | 61 | 35 / 6.6 | 30.6 | *ALAD* | Delta-aminolevulinic acid dehydratase isoform a (39kD, pI 7.6) | | 35 |
|  |  |  | 15 | *C1orf80* | Hypothetical protein LOC64853 (35kD, pI 6.6) | | 6 |
| 96 | 62 | 48 / 6.7 | 44.7 | *PSMD11* | Proteasome 26S non-ATPase subunit 11 variant (Fragment) | | 64 |
|  |  |  |  |  | (47.5kD, pI 6.5) | |  |
|  |  |  | 23.2 | *PA2G4* | Proliferation-associated 2G4, 38kDa (45.1kD, pI 8.9) | | 12 |
| 200 | 63 | 100 / 5.2 | 38.5 | *VCP* | Valosin-containing protein (89.3kD, pI 5.2) | | 42 |
|  |  |  | 19.9 | *PSMD11* | Proteasome 26S non-ATPase subunit 11 variant (47.5kD, pI 6.5) | | 12 |
|  |  |  |  |  | carry over | |  |
|  |  |  | 13.5 | *TGM2* | Isoform 1 of Protein-glutamine gamma-glutamyltransferase 2 | | 9 |
|  |  |  |  |  | (77.3kD, pI 5.2) | |  |
|  |  |  | 9.4 | *PSMD2* | 26S proteasome non-ATPase regulatory subunit 2 (100kD, pI 5.2) | | 7 |
|  |  |  | 8.9 | *PA2G4* | Proliferation-associated 2G4, 38kDa (45.1kD, pI 8.9) carry over | | 3 |
|  |  |  | 3.4 | *USP5* | Ubiquitin specific peptidase 5 (isopeptidase T) (95.7kD, pI 5.0) | | 3 |
| 292 | 64 | 37 / 6.8 | 21.9 | *PSMD7* | 26S proteasome non-ATPase regulatory subunit 7 (37kD, pI 6.8) | | 8 |
|  |  |  | 17.4 | *GMPR* | GMP reductase 1 (37.4kD, pI 7.1) | | 5 |
|  |  |  | 16.9 | *VCP* | Valosin-containing protein (89.3kD, pI 5.2) carry over | | 12 |
| 281 | 65 | 45 / 7.5 | 46.1 | *PSMC5* | 26S protease regulatory subunit 8 (45.6kD, pI 7.5) | | 63 |
| 1 | 66 | 30 / 6.5 | 66.3 | *PNP* | Purine Nucleoside Phosphorylase (33kD, pI 7.2) | | 37 |
|  |  |  | 20.1 | *PSMD7* | 26S proteasome non-ATPase regulatory subunit 7 (37kD, pI 6.8) | | 6 |
|  |  |  |  |  | carry over | |  |
|  |  |  | 17.4 | *GMPR* | GMP reductase 1 (37.4kD, pI 7.1) carry over | | 4 |
|  |  |  | 10.6 | *PSMC5* | 26S protease regulatory subunit 8 (45.6kD, pI 7.5) carry over | | 5 |
| 164 | 67 | 26 / 8.1 | 56.9 | *PSMA7* | Isoform 1 of Proteasome subunit alpha type-7 (28kD, pI 8.5) | | 27 |
| 165 | 68 | 40 / 6.6 | 38.6 | *TSTA3* | GDP-L-fucose synthetase (35.8kD, pI 6.6) | | 25 |
| 199 | 69 | 17 / 6.4 | 32.3 | *NME1/*  *NME2* | Nucleosidee diphosphate kinase (32.6kD, pI 8.5 )  NM23-H1B of Nucleosidee diphosphate kinase A (19.6kD, pI 5.4) | | 15 |
| 121 | 70 | 10 / 5.0 | 13 | *EIF5A* | Isoform 2 of Eukaryotic translation initiation factor 5A-1 | | 14 |
|  |  |  |  |  | (20.1kD, pI 7.0) | |  |
| 147 | 71 | 100 / 5.0 | 26 | *KPNB1* | Karyopherin (importin) subunit beta 1 (97.1kD, pI 4.8) | | 49 |
| 479 | 72 | 49 / 6.5 | 27.3 | *BLMH* | Bleomycin hydrolase (52.5 kD, pI 6.3) | | 29 |
|  |  |  | 20.6 | *PSMD11* | Proteasome 26S non-ATPase subunit 11 variant (47.5kD, pI 6.5) | | 11 |
|  |  |  | 16.2 | *GDI2* | Rab GDP dissociation inhibitor beta (50.6kD, pI 6.5) | | 6 |
|  |  |  | 6.5 | *ST13* | Hsc70-interacting protein (41.3kD, pI 5.3) | | 2 |
|  |  |  | 4.8 | *KPNB1* | Karyopherin (importin) subunit beta 1 (97.1kD, pI 4.8) carry over | | 4 |
| 106 | 73 | 65 / 6.5 | 43.7 | *TCP1* | T-complex protein 1 subunit alpha (60kD, pI 6.1) | | 50 |
|  |  |  | 4.9 | *OXSR1* | Serine/threonine-protein kinase OSR1 (58kD, pI 6.4) | | 3 |
| 327 | 74 | 60 / 5.8 | 42.5 | *USP14* | Ubiquitin carboxyl-terminal hydrolase 14 (56kD, pI 5.3) | | 69 |
|  |  |  | 10.1 | *APPBP1* | NEDD8 activating enzyme E1 subunit 1 (60.5kD, pI 5.4) | | 4 |
|  |  |  | 6.5 | *TCP1* | T-complex protein 1 subunit alpha (60kD, pI 6.1) carry over | | 4 |
| 260 | 75 | 22 / 7.0 | 34.5 | *PSMB2* | Proteasome beta 2 subunit variant (Fragment) (27.8kD, pI 9.3) | | 28 |
|  |  |  | 28.2 | *TCP1* | T-complex protein 1 subunit alpha (60kD, pI 6.1) carry over | | 18 |
|  |  |  | 27.5 | *USP14* | Ubiquitin carboxyl-terminal hydrolase 14 (56kD, pI 5.3) carry over | | 26 |
|  |  |  | 22.5 | *PSMB2* | Proteasome beta 2 subunit variant (Fragment) (27.8kD, pI 9.3) | | 11 |
|  |  |  | 18.2 | *PRDX2* | Peroxiredoxin-2 (21.8kD, pI 6.0) | | 4 |
| 118 | 76 | 60 / 6.8 | 35.2 | *CCT6A* | T-complex protein 1 subunit zeta 1 (58kD, pI 6.7) | | 37 |
|  |  |  | 11.1 | *CCT6B* | T-complex protein 1 subunit zeta-2 (57.7kD, pI 7.1) | | 9 |
| 537 | 77 | 50 / 6.1 | 30.8 | *GSS* | Glutathione synthetase (52.3kD, pI 5.9) | | 33 |
|  |  |  | 7 | *CCT6A* | T-complex protein 1 subunit zeta (58kD, pI 6.7) carry over | | 4 |
|  |  |  | 6.9 | *RUVBL2* | RuvB-like 2 (E. coli) (51.1kD, pI 5.6) | | 2 |
|  |  |  | 5.7 | *SH3GLB2* | SH3-domain GRB2-like endophilin B2 (44.7kD, pI 5.6) | | 2 |
| 74 | 78 | 60 / 6.8 | 42.2 | *CCT3* | Chaperonin containing TCP1, subunit 3 (gamma) (60.5kD, pI 6.5) | | 68 |
|  |  |  | 16.2 | *GSS* | Glutathione synthetase (52.3kD, pI 5.9) carry over | | 7 |
|  |  |  | 10.2 | *CCT6A* | T-complex protein 1 subunit zeta (58kD, pI 6.7) | | 6 |
|  |  |  | 7 | *DCAF11* | DDB1 and CUL4 associated factor 11 (61.6kD, pI 6.4) | | 2 |
|  |  |  | 5.3 | *FBXO7* | F-box protein 7 (58kD, pI 6.8) | | 2 |
|  |  |  | 4.9 | *ARCN1* | Archain 1 coatomer protein delta-COP (61.6kD, pI 5.8) | | 3 |
| 132 | 79 | 36 / 6.5 | 27.6 | *ALAD* | Delta-aminolevulinic acid dehydratase isoform a (39kD, pI 7.6) | | 30 |
| 406 | 80 | 36 / 6.5 | 34 | *ALAD* | Delta-aminolevulinic acid dehydratase isoform a (39kD, pI 7.6) | | 52 |
|  |  |  | 27.4 | *PSMD14* | 26S proteasome non-ATPase regulatory SU 14 (34.5kD, pI 6.5) | | 12 |
|  |  |  | 18 | *C1orf80* | Hypothetical protein LOC64853 (35kD, pI 6.6) | | 8 |
|  |  |  | 0.6 | *TET1* | Tet oncogene 1 CXXC-type zinc finger protein 6 (235kD, pI 8.3) | | 3 |
| 145 | 81 | 28 / 7.1 | 57.5 | *PSMA4* | Proteasome subunit alpha type-4 (29.4kD, pI 7.7) | | 58 |
|  |  |  | 30.3 | *CA1* | Carbonic anhydrase 1(28.8kD, pI 7.1) | | 12 |
|  |  |  | 8.1 | *ALAD* | Delta-aminolevulinic acid dehydratase isoform a (39kD, pI 7.6) | | 2 |
|  |  |  |  |  | carry over | |  |
| 101 | 82 | 62 / 8.0 | 37.1 | *CCT4* | T-complex protein 1 subunit delta (57.9kD, pI 7.8) | | 66 |
|  |  |  | 24.5 | *CCT7* | T-complex protein 1 subunit eta (59.4kD, pI 7.6) | | 18 |
|  |  |  | 21.5 | *PSMA4* | Proteasome subunit alpha type-4 (29.4kD, pI 7.7) carry over | | 4 |
| 102 | 83 | 50 / 6.5 | 53 | *GDI2* | Rab GDP dissociation inhibitor beta (50.6kD, pI 6.5) | | 50 |
|  |  |  | 21.3 | *BLMH* | Bleomycin hydrolase (52.5 kD, pI 6.3) | | 19 |
|  |  |  | 4.6 | *CCT4* | T-complex protein 1 subunit delta (57.9kD, pI 7.8) carry over | | 6 |
| 421 | 84 | 30 / 5.5 | 60.6 | *CLIC1* | Chloride intracellular channel protein 1 (26.9kD, pI 5.2) | | 45 |
|  |  |  | 20.7 | *GDI2* | Rab GDP dissociation inhibitor beta (50.6kD, pI 6.5) carry over | | 9 |
|  |  |  | 12.1 | *PSME2* | Proteasome activator subunit 2 (PA28 beta) (27.4kD, pI 5.7) | | 2 |
|  |  |  | 5.9 | *BLMH* | Bleomycin hydrolase (52.5 kD, pI 6.3) carry over | | 3 |
| 184 | 85 | 60 / 6.6 | 46.5 | *CCT2* | Chaperonin containing TCP1, subunit 2 (beta) (57.5kD, pI 6.4) | | 86 |
|  |  |  | 3.8 | *DARS* | Aspartyl-tRNA synthetase, cytoplasmic (57kD, pI 6.5) | |  |
| 387 | 86 | 22 / 6.5 | 35.1 | *CCT2* | T-complex protein 1, subunit 2 (beta) (57.5kD, pI 6.4) carry over | | 37 |
|  |  |  | 30.3 | *PRDX2* | Peroxiredoxin-2 (21.8kD, pI 6.0) | 25 | |
| 491 | 87 | 26 / 7.1 | 30.8 | *PSMA2* | Proteasome subunit, alpha type, 2 (25.9kD, pI 7.4) | | 8 |
| 251 | 88 | 30 / 6.1 | 39.7 | *PSMA2* | Proteasome subunit, alpha type, 2 (25.9kD, pI 7.4) carry over | | 22 |
|  |  |  | 29.6 | *PSME1* | Proteasome (activator subunit 1 (PA28 alpha) (28.7kD, pI 5.7) | | 44 |
|  |  |  | 12.6 | *PSME2* | Proteasome activator subunit 2 (PA28 beta) (27.4kD, pI 5.7) | | 2 |
|  |  |  | 12.2 | *PAFAH1B2* | Platelet-activating factor acetylhydrol. IB SU beta (25.6kD,pI 5.9) | 3 | |
| 91 | 89 | 60 / 7.5 | 53 | *CCT7* | T-complex protein 1 subunit eta (59.4kD, pI 7.6) | | 61 |
|  |  |  | 25.6 | *CCT4* | T-complex protein 1 subunit delta (57.9kD, pI 7.8) | | 17 |
|  |  |  | 14.2 | *PSME1* | Proteasome activator subunit 1 (28.7kD, pI 5.7) carry over | 2 | |
| 283 | 90 | 60 / 6.0 | 44 | *CCT8* | Chaperonin containing TCP1, subunit 8 (theta) (59.7kD, pI 5.6) | | 73 |
|  |  |  | 12 | *CCT5* | Chaperonin containing TCP1, subunit 5 (epsilon) (59.6kD, pI 5.6) | | 7 |
|  |  |  | 7 | *CCT7* | T-complex protein 1 subunit eta (59.4kD, pI 7.6) carry over | | 3 |
|  |  |  | 5.6 | *EIF5* | Eukaryotic translation initiation factor 5 (49.2kD, pI 5.6) | 4 | |
| 487 | 91 | 16 / 4.5 | 31.6 | *CCT8* | T-complex protein 1 subunit 8 (theta) (59.7kD, pI 5.6) carry over | | 26 |
|  |  |  | 8.6 | *SKP1* | Isoform 1 of S-phase kinase-associated protein 1A (18.6kD, pI 4.5) | | 2 |
|  |  |  | 6.8 | *CCT7* | T-complex protein 1 subunit eta (59.4kD, pI 7.6) carry over | 2 | |
| 263 | 92 | 35 / 6.6 | 30.6 | *ALAD* | Delta-aminolevulinic acid dehydratase isoform a (39kD, pI 7.6) | | 44 |
|  |  |  | 9.3 | *LDHB* | L-lactate dehydrogenase B chain (36.6kD, pI 6.1) | | 2 |
|  |  |  | 0.2 | *HERC1* | Guanine nucleotide exchange factor p532 (523kD, pI 6.0) | 2 | |
| 477 | 93 | 29 / 5.9 | 31.8 | *PSME2* | Proteasome activator subunit 2 (PA28 beta) (27.4kD, pI 5.7) | | 20 |
|  |  |  | 23.1 | *ALAD* | Delta-aminolevulinic acid dehydr. Isof. a (39kD, pI 7.6) carry over | | 14 |
|  |  |  | 23.1 | *CLIC1* | Chloride intracellular channel protein 1 (26.9kD, pI 5.2) | | 7 |
|  |  |  | 9.9 | *PSME1* | Proteasome activator subunit 1 (PA28 alpha) (28.7kD, pI 5.7) | 3 | |
| 663 | 94 | 35 / 6.8 | 39.8 | *ALAD* | Delta-aminolevulinic acid dehydratase isoform a (39kD, pI 7.6) | | 50 |
| 631 | 95 | 35 / 6.2 | 52.7 | *LDHB* | L-lactate dehydrogenase B chain (36.6kD, pI 6.1) | | 56 |
|  |  |  | 13.4 | *ALAD* | Delta-aminolevulinic acid dehydratase isoform a (39kD, pI 7.6) | | 33 |
|  |  |  | 6.9 | *LDHA* | Isoform 1 of L-lactate dehydrogenase A chain (36.6kD, pI 8.3) | 3 | |
| 416 | 96 | 22 / 6.0 | 36.9 | *PRDX2* | Peroxiredoxin-2 (21.8kD, pI 6.0) | | 50 |
|  |  |  | 0.8 | *SBNO1 / 2* | Strawberry notch homolog 1/2 (Drosophila) (154kD, pI 7.9 or 6.8) | | 2 |
|  |  |  | 9.3 | *LDHB* | L-lactate dehydrogenase B chain (36.6kD, pI 6.1) | | 4 |
| # 661 | 97 | 35 / 8.1 | 44.9 | *LDHA* | Isoform 1 of L-lactate dehydrogenase A chain (36.6kD, pI 8.3) | | 34 |
| # 632 | 98 | 35 / 4.5 | 8.3 | *RAD23A* | UV excision repair protein RAD23 homolog A (39.6kD, pI 4.6) | | 3 |
| #306 | 99 | 60 / 4.7 | 4.7 | *CAST* | Calpastatin isoform a (84.9kD, pI 5.4) | | 10 |

Spot rank: Rank of spot as assigned by Same Spots Software depending on fold change (normalized volume) comparing the highest to lowest sample

Pick #: Sequence in which spots were excised from gel depending on spot intensity (from lowest to highest)

Spot Location: Approximate location of spot by protein mass (marker on gel in kilo dalton, kD) and isoelectric point (pI) as estimated on focussing strip used

Coverage: Percentage of aminoacid sequence that is covered by unique peptides to identify protein

Gene: HGNC Symbol for coding human gene

Protein: HGNC Symbol for protein identified (including theoretical protein mass and pI)

SC: Spectral count
