# Supplementary material for: Assessment of the Red Cell Proteome of Young Patients with Unexplained Hemolytic Anemia by Two-Dimensional Differential In-Gel Electrophoresis (DIGE)
Source: PLoS One. 2012 Apr 3;7(4):e34237. doi: 10.1371/journal.pone.0034237 (PMC3317954; doi:10.1371/journal.pone.0034237)
Supplement: Table S13 — Complete list of all spots picked in HA21, fold change comparing patient to all other samples run in the experiment. (DOCX) [file pone.0034237.s017.docx]

# Table S13: Complete list of all spots picked in HA21, fold change comparing patient to all other samples run in the experiment

| Spot rank | | Pick # | | Fold change | |  | | Patient | | Mother | | Father | | Control | | Standard | |  |
| --- | --- | --- | --- | --- | --- | --- | --- | --- | --- | --- | --- | --- | --- | --- | --- | --- | --- | --- |
| 20 | | 1 | |  | | I | | 3.370 | | 0.788 | | 0.669 | | 0.696 | | 0.650 | |  |
|  | |  | |  | | II | | 3.084 | | 0.798 | | 0.718 | | 0.615 | | 0.659 | |  |
|  | |  | |  | | med | | 3.227 | | 0.793 | | 0.694 | | 0.656 | | 0.655 | |  |
|  | | **P to all 4.62** | | | |  | |  | | **4.069** | | **4.653** | | **4.923** | | **4.930** | |  |
| 5 | | 2 | |  | | I | | 2.991 | | 0.434 | | 0.517 | | 0.588 | | 0.462 | |  |
|  | |  | |  | | II | | 3.014 | | 0.422 | | 0.451 | | 0.524 | | 0.410 | |  |
|  | |  | |  | | med | | 3.003 | | 0.428 | | 0.484 | | 0.556 | | 0.436 | |  |
|  | | **P to all 6.31** | | | |  | |  | | **7.015** | | **6.204** | | **5.400** | | **6.886** | |  |
| 29 | | 3 | |  | | I | | 3.062 | | 1.382 | | 1.625 | | 1.041 | | 0.722 | |  |
|  | |  | |  | | II | | 2.972 | | 1.541 | | 1.388 | | 0.882 | | 0.734 | |  |
|  | |  | |  | | med | | 3.017 | | 1.462 | | 1.507 | | 0.962 | | 0.728 | |  |
|  | | **P to all 2.59** | | | |  | |  | | **2.064** | | **2.003** | | **3.138** | | **4.144** | |  |
| 7 | | 4 | |  | | I | | 3.046 | | 0.700 | | 0.612 | | 0.499 | | 0.456 | |  |
|  | |  | |  | | II | | 3.166 | | 0.506 | | 0.581 | | 0.569 | | 0.530 | |  |
|  | |  | |  | | med | | 3.106 | | 0.603 | | 0.597 | | 0.534 | | 0.493 | |  |
|  | | **P to all 5.58** | | | |  | |  | | **5.151** | | **5.207** | | **5.816** | | **6.300** | |  |
| 232 | | 5 | |  | | I | | 1.576 | | 0.767 | | 0.769 | | 0.886 | | 1.147 | |  |
|  | |  | |  | | II | | 1.575 | | 0.830 | | 0.800 | | 0.824 | | 1.070 | |  |
|  | |  | |  | | med | | 1.576 | | 0.799 | | 0.785 | | 0.855 | | 1.109 | |  |
|  | | **P to all 1.78** | | | |  | |  | | **1.973** | | **2.008** | | **1.843** | | **1.421** | |  |
| 133 | | 6 | |  | | I | | 0.504 | | 1.182 | | 1.263 | | 1.193 | | 1.049 | |  |
|  | |  | |  | | II | | 0.518 | | 1.195 | | 1.312 | | 1.119 | | 1.157 | |  |
|  | |  | |  | | med | | 0.511 | | 1.189 | | 1.288 | | 1.156 | | 1.103 | |  |
|  | | *P to all 2.32* | | | |  | |  | | *2.33* | | *2.52* | | *2.26* | | *2.16* | |  |
| 21 | | 7 | |  | | I | | 1.862 | | 0.391 | | 0.593 | | 0.612 | | 0.500 | |  |
|  | |  | |  | | II | | 1.817 | | 0.399 | | 0.559 | | 0.574 | | 0.499 | |  |
|  | |  | |  | | med | | 1.840 | | 0.395 | | 0.576 | | 0.593 | | 0.500 | |  |
|  | | **P to all 3.57** | | | |  | |  | | **4.657** | | **3.194** | | **3.102** | | **3.683** | |  |
| 3 | | 8 | |  | | I | | 3.496 | | 0.395 | | 0.487 | | 0.511 | | 0.367 | |  |
|  | |  | |  | | II | | 3.116 | | 0.419 | | 0.424 | | 0.510 | | 0.346 | |  |
|  | |  | |  | | med | | 3.306 | | 0.407 | | 0.456 | | 0.511 | | 0.357 | |  |
|  | | **P to all 7.65** | | | |  | |  | | **8.123** | | **7.258** | | **6.476** | | **9.273** | |  |
| 298 | | 9 | |  | | I | | 1.119 | | 0.641 | | 0.832 | | 0.939 | | 0.928 | |  |
|  | |  | |  | | II | | 1.218 | | 0.623 | | 0.863 | | 0.912 | | 0.773 | |  |
|  | |  | |  | | med | | 1.169 | | 0.632 | | 0.848 | | 0.926 | | 0.851 | |  |
|  | | P to all 1.44 | | | |  | |  | | 1.849 | | 1.379 | | 1.263 | | 1.374 | |  |
| 98 | | 10 | |  | | I | | 3.082 | | 0.956 | | 1.118 | | 1.358 | | 1.025 | |  |
|  | |  | |  | | II | | 2.489 | | 1.042 | | 0.982 | | 1.305 | | 1.007 | |  |
|  | |  | |  | | med | | 2.786 | | 0.999 | | 1.050 | | 1.332 | | 1.016 | |  |
|  | | **P to all 2.53** | | | |  | |  | | **2.788** | | **2.653** | | **2.092** | | **2.742** | |  |
| 151 | | 11 | |  | | I | | 0.360 | | 0.732 | | 0.837 | | 0.883 | | 0.906 | |  |
|  | |  | |  | | II | | 0.425 | | 0.701 | | 0.877 | | 0.881 | | 0.975 | |  |
|  | |  | |  | |  | | 0.393 | | 0.717 | | 0.857 | | 0.882 | | 0.941 | |  |
|  | | *P to all 2.16* | | | | *med* | |  | | *1.83* | | *2.18* | | *2.25* | | *2.40* | |  |
| 108 | | 12 | |  | | I | | 1.842 | | 1.015 | | 1.055 | | 0.583 | | 1.048 | |  |
|  | |  | |  | | II | | 1.590 | | 1.115 | | 0.911 | | 0.678 | | 0.917 | |  |
|  | |  | |  | | med | | 1.716 | | 1.065 | | 0.983 | | 0.631 | | 0.983 | |  |
|  | | **P to all 1.87** | | | |  | |  | | **1.611** | | **1.746** | | **2.722** | | **1.747** | |  |
| 27 | | 13 | |  | | I | | 2.197 | | 0.457 | | 0.609 | | 0.544 | | 0.495 | |  |
|  | |  | |  | | II | | 2.021 | | 0.517 | | 0.616 | | 0.747 | | 0.719 | |  |
|  | |  | |  | | med | | 2.109 | | 0.487 | | 0.613 | | 0.646 | | 0.607 | |  |
|  | | **P to all 3.59** | | | |  | |  | | **4.331** | | **3.443** | | **3.267** | | **3.474** | |  |
| 258 | | 14 | |  | | I | | 0.499 | | 0.696 | | 0.698 | | 0.929 | | 0.926 | |  |
|  | |  | |  | | II | | 0.508 | | 0.734 | | 0.646 | | 1.050 | | 0.903 | |  |
|  | |  | |  | | med | | 0.504 | | 0.715 | | 0.672 | | 0.990 | | 0.915 | |  |
|  | | *P to all 1.63* | | | |  | |  | | *1.42* | | *1.33* | | *1.97* | | *1.82* | |  |
| 87 | | 15 | |  | | I | | 0.442 | | 1.408 | | 1.162 | | 0.799 | | 0.807 | |  |
|  | |  | |  | | II | | 0.479 | | 1.372 | | 1.259 | | 0.720 | | 0.688 | |  |
|  | |  | |  | | med | | 0.461 | | 1.390 | | 1.211 | | 0.760 | | 0.748 | |  |
|  | | *P to all 2.23* | | | |  | |  | | *3.02* | | *2.63* | | *1.65* | | *1.62* | |  |
| 45 | | 16 | |  | | I | | 0.337 | | 1.202 | | 1.415 | | 1.273 | | 1.010 | |  |
|  | |  | |  | | II | | 0.408 | | 1.174 | | 1.290 | | 1.227 | | 1.145 | |  |
|  | |  | |  | | med | | 0.373 | | 1.188 | | 1.353 | | 1.250 | | 1.078 | |  |
|  | | *P to all 3.27* | | | |  | |  | | *3.19* | | *3.63* | | *3.36* | | *2.89* | |  |
| 389 | | 17 | |  | | I | | 1.705 | | 1.083 | | 1.115 | | 1.245 | | 1.104 | |  |
|  | |  | |  | | II | | 1.876 | | 1.160 | | 1.037 | | 1.275 | | 1.133 | |  |
|  | |  | |  | | med | | 1.791 | | 1.122 | | 1.076 | | 1.260 | | 1.119 | |  |
|  | | **P to all 1.57** | | | |  | |  | | **1.597** | | **1.664** | | **1.421** | | **1.601** | |  |
| 12 | | 18 | |  | | I | | 2.898 | | 0.576 | | 0.484 | | 0.525 | | 0.683 | |  |
|  | |  | |  | | II | | 2.173 | | 0.686 | | 0.434 | | 0.663 | | 0.573 | |  |
|  | |  | |  | | med | | 2.536 | | 0.631 | | 0.459 | | 0.594 | | 0.628 | |  |
|  | | **P to all 4.93** | | | |  | |  | | **4.018** | | **5.524** | | **4.269** | | **4.037** | |  |
| 48 | | 19 | |  | | I | | 1.699 | | 0.792 | | 0.706 | | 0.680 | | 0.485 | |  |
|  | |  | |  | | II | | 1.677 | | 0.793 | | 0.746 | | 0.733 | | 0.455 | |  |
|  | |  | |  | |  | | 1.688 | | 0.793 | | 0.726 | | 0.707 | | 0.470 | |  |
|  | | **P to all 2.51** | | | | **med** | |  | | **2.130** | | **2.325** | | **2.389** | | **3.591** | |  |
| 103 | | 20 | |  | | I | | 0.393 | | 1.138 | | 0.541 | | 1.147 | | 0.862 | |  |
|  | |  | |  | | II | | 0.424 | | 1.074 | | 0.565 | | 1.108 | | 0.887 | |  |
|  | |  | |  | | med | | 0.409 | | 1.106 | | 0.553 | | 1.128 | | 0.875 | |  |
|  | | *P to all 2.24* | | | |  | |  | | *2.71* | | *1.35* | | *2.76* | | *2.14* | |  |
| 6 | | 21 | |  | | I | | 3.249 | | 0.500 | | 0.643 | | 0.579 | | 0.496 | |  |
|  | |  | |  | | II | | 3.273 | | 0.503 | | 0.612 | | 0.726 | | 0.761 | |  |
|  | |  | |  | | med | | 3.261 | | 0.502 | | 0.628 | | 0.653 | | 0.629 | |  |
|  | | **P to all 5.41** | | | |  | |  | | **6.502** | | **5.197** | | **4.998** | | **5.189** | |  |
| 116 | | 22 | |  | | I | | 0.512 | | 1.137 | | 1.250 | | 1.061 | | 1.299 | |  |
|  | |  | |  | | II | | 0.489 | | 1.058 | | 1.202 | | 1.021 | | 1.373 | |  |
|  | |  | |  | | med | | 0.501 | | 1.098 | | 1.226 | | 1.041 | | 1.336 | |  |
|  | | *P to all 1.97* | | | |  | |  | | *2.19* | | *2.45* | | *2.08* | | *2.67* | |  |
| 188 | | 23 | |  | | I | | 0.566 | | 1.053 | | 1.265 | | 1.028 | | 1.121 | |  |
|  | |  | |  | | II | | 0.550 | | 1.035 | | 1.206 | | 1.090 | | 1.013 | |  |
|  | |  | |  | | med | | 0.558 | | 1.044 | | 1.236 | | 1.059 | | 1.067 | |  |
|  | | *P to all 1.97* | | | |  | |  | | *1.87* | | *2.21* | | *1.90* | | *1.91* | |  |
| 61 | | 24 | |  | | I | | 0.436 | | 0.554 | | 1.472 | | 1.161 | | 1.168 | |  |
|  | |  | |  | | II | | 0.464 | | 0.548 | | 1.574 | | 1.099 | | 1.221 | |  |
|  | |  | |  | | med | | 0.450 | | 0.551 | | 1.523 | | 1.130 | | 1.195 | |  |
|  | | *P all 2.44* | | | |  | |  | | *1.22* | | *3.38* | | *2.51* | | *2.65* | |  |
| 51 | | 25 | |  | | I | | 0.674 | | 1.218 | | 2.292 | | 1.451 | | 1.846 | |  |
|  | |  | |  | | II | | 0.540 | | 1.570 | | 1.963 | | 1.495 | | 1.454 | |  |
|  | |  | |  | | med | | 0.607 | | 1.394 | | 2.128 | | 1.473 | | 1.650 | |  |
|  | | *P to all 2.74* | | | |  | |  | | *2.30* | | *3.50* | | *2.43* | | *2.72* | |  |
| 204 | | 26 | |  | | I | | 0.465 | | 0.652 | | 0.810 | | 0.985 | | 0.815 | |  |
|  | |  | |  | | II | | 0.472 | | 0.626 | | 0.800 | | 1.026 | | 0.797 | |  |
|  | |  | |  | | med | | 0.469 | | 0.639 | | 0.805 | | 1.006 | | 0.806 | |  |
|  | | *P to all 1.74* | | | |  | |  | | *1.36* | | *1.72* | | *2.15* | | *1.72* | |  |
| 304 | | 27 | |  | | I | | 0.505 | | 0.774 | | 0.878 | | 0.825 | | 0.911 | |  |
|  | |  | |  | | II | | 0.465 | | 0.782 | | 0.843 | | 0.901 | | 0.876 | |  |
|  | |  | |  | | med | | 0.485 | | 0.778 | | 0.861 | | 0.863 | | 0.894 | |  |
|  | | *P to all 1.75* | | | |  | |  | | *1.60* | | *1.77* | | *1.78* | | *1.84* | |  |
| 127 | | 28 | |  | | I | | 0.551 | | 1.157 | | 1.307 | | 1.084 | | 1.419 | |  |
|  | |  | |  | | II | | 0.525 | | 1.185 | | 1.357 | | 1.115 | | 1.356 | |  |
|  | |  | |  | | med | | 0.538 | | 1.171 | | 1.332 | | 1.100 | | 1.388 | |  |
|  | | *P to all 2.32* | | | |  | |  | | *2.18* | | *2.48* | | *2.04* | | *2.58* | |  |
| 72 | | 29 | |  | | I | | 0.303 | | 0.794 | | 0.936 | | 0.964 | | 0.992 | |  |
|  | |  | |  | | II | | 0.323 | | 0.751 | | 0.967 | | 0.915 | | 1.006 | |  |
|  | |  | |  | | med | | 0.313 | | 0.773 | | 0.952 | | 0.940 | | 0.999 | |  |
|  | | *P to all 2.93* | | | |  | |  | | *2.47* | | *3.04* | | *3.00* | | *3.19* | |  |
| 25 | | 30 | |  | | I | | 2.626 | | 0.764 | | 0.651 | | 0.600 | | 0.614 | |  |
|  | |  | |  | | II | | 2.979 | | 0.721 | | 0.652 | | 0.681 | | 0.778 | |  |
|  | |  | |  | | med | | 2.803 | | 0.743 | | 0.652 | | 0.641 | | 0.696 | |  |
|  | | **P to all 4.11** | | | |  | |  | | **3.774** | | **4.302** | | **4.375** | | **4.027** | |  |
| 158 | | 31 | |  | | I | | 1.335 | | 0.709 | | 0.619 | | 0.551 | | 0.559 | |  |
|  | |  | |  | | II | | 1.316 | | 0.662 | | 0.522 | | 0.582 | | 0.631 | |  |
|  | |  | |  | | med | | 1.326 | | 0.686 | | 0.571 | | 0.567 | | 0.595 | |  |
|  | | **P to all 2.19** | | | |  | |  | | **1.934** | | **2.323** | | **2.340** | | **2.228** | |  |
| 67 | | 32 | |  | | I | | 1.641 | | 0.728 | | 0.864 | | 0.560 | | 0.515 | |  |
|  | |  | |  | | II | | 1.629 | | 0.758 | | 0.855 | | 0.522 | | 0.502 | |  |
|  | |  | |  | | med | | 1.635 | | 0.743 | | 0.860 | | 0.541 | | 0.509 | |  |
|  | | **P to all 2.47** | | | |  | |  | | **2.201** | | **1.902** | | **3.022** | | **3.215** | |  |
| 559 | | 33 | |  | | I | | 1.077 | | 1.041 | | 1.471 | | 1.505 | | 1.401 | |  |
|  | |  | |  | | II | | 1.035 | | 1.092 | | 1.342 | | 1.442 | | 1.424 | |  |
|  | |  | |  | | med | | 1.056 | | 1.067 | | 1.407 | | 1.474 | | 1.413 | |  |
|  | | P to all 1.27 | | | |  | |  | | 1.01 | | 1.33 | | 1.40 | | 1.34 | |  |
| 291 | | 34 | |  | | I | | 0.643 | | 1.090 | | 0.843 | | 0.844 | | 1.144 | |  |
|  | |  | |  | | II | | 0.559 | | 1.052 | | 0.871 | | 0.868 | | 1.117 | |  |
|  | |  | |  | | med | | 0.601 | | 1.071 | | 0.857 | | 0.856 | | 1.131 | |  |
|  | | *P to all 1.63* | | | |  | |  | | *1.78* | | *1.43* | | *1.42* | | *1.88* | |  |
| 143 | | 35 | |  | | I | | 0.602 | | 1.428 | | 1.257 | | 1.400 | | 1.180 | |  |
|  | |  | |  | | II | | 0.612 | | 1.509 | | 1.269 | | 1.364 | | 1.099 | |  |
|  | |  | |  | | med | | 0.607 | | 1.469 | | 1.263 | | 1.382 | | 1.140 | |  |
|  | | *P to all 2.16* | | | |  | |  | | *2.42* | | *2.08* | | *2.28* | | *1.88* | |  |
| 150 | | 36 | |  | | I | | 0.553 | | 1.027 | | 1.312 | | 1.202 | | 1.002 | |  |
|  | |  | |  | | II | | 0.484 | | 1.238 | | 1.137 | | 1.277 | | 0.880 | |  |
|  | |  | |  | | med | | 0.519 | | 1.133 | | 1.225 | | 1.240 | | 0.941 | |  |
|  | | *P to all 2.19* | | | |  | |  | | *2.18* | | *2.36* | | *2.39* | | *1.81* | |  |
| 32 | | 37 | |  | | I | | 0.479 | | 1.951 | | 1.228 | | 1.372 | | 1.539 | |  |
|  | |  | |  | | II | | 0.551 | | 1.743 | | 1.185 | | 1.786 | | 2.673 | |  |
|  | |  | |  | | med | | 0.515 | | 1.847 | | 1.207 | | 1.579 | | 2.106 | |  |
|  | | *P to all 3.27* | | | |  | |  | | *3.59* | | *2.34* | | *3.07* | | *4.09* | |  |
| 47 | | 38 | |  | | I | | 0.414 | | 1.182 | | 1.678 | | 1.361 | | 0.787 | |  |
|  | |  | |  | | II | | 0.393 | | 1.282 | | 1.264 | | 1.332 | | 0.603 | |  |
|  | |  | |  | | med | | 0.404 | | 1.232 | | 1.471 | | 1.347 | | 0.695 | |  |
|  | | *P to all 2.94* | | | |  | |  | | *3.05* | | *3.65* | | *3.34* | | *1.72* | |  |
| 544 | | 39 | |  | | I | | 0.694 | | 0.794 | | 0.969 | | 0.900 | | 0.994 | |  |
|  | |  | |  | | II | | 0.691 | | 0.796 | | 0.955 | | 0.903 | | 0.970 | |  |
|  | |  | |  | | med | | 0.693 | | 0.795 | | 0.962 | | 0.902 | | 0.982 | |  |
|  | | P to all 1.31 | | | |  | |  | | 1.15 | | 1.39 | | 1.30 | | 1.42 | |  |
| 77 | | 40 | |  | | I | | 0.285 | | 0.798 | | 0.850 | | 1.043 | | 0.820 | |  |
|  | |  | |  | | II | | 0.350 | | 0.697 | | 0.897 | | 0.922 | | 0.859 | |  |
|  | |  | |  | | med | | 0.318 | | 0.748 | | 0.874 | | 0.983 | | 0.840 | |  |
|  | | *P to all 2.71* | | | |  | |  | | *2.35* | | *2.75* | | *3.09* | | *2.64* | |  |
| 8 | | 41 | |  | | I | | 0.199 | | 1.026 | | 0.940 | | 1.009 | | 1.225 | |  |
|  | |  | |  | | II | | 0.202 | | 1.034 | | 0.973 | | 0.881 | | 1.231 | |  |
|  | |  | |  | | med | | 0.201 | | 1.030 | | 0.957 | | 0.945 | | 1.228 | |  |
|  | | *P to all 5.19* | | | |  | |  | | *5.14* | | *4.77* | | *4.71* | | *6.12* | |  |
| 86 | | 42 | |  | | I | | 1.473 | | 0.795 | | 0.561 | | 0.483 | | 0.743 | |  |
|  | |  | |  | | II | | 1.598 | | 0.711 | | 0.606 | | 0.538 | | 0.843 | |  |
|  | |  | |  | | med | | 1.536 | | 0.753 | | 0.584 | | 0.511 | | 0.793 | |  |
|  | | **P to all 2.33** | | | |  | |  | | **2.039** | | **2.632** | | **3.008** | | **1.936** | |  |
| 69 | | 43 | |  | | I | | 2.029 | | 0.935 | | 0.895 | | 1.029 | | 0.613 | |  |
|  | |  | |  | | II | | 2.034 | | 0.943 | | 0.856 | | 1.108 | | 0.657 | |  |
|  | |  | |  | | med | | 2.032 | | 0.939 | | 0.876 | | 1.069 | | 0.635 | |  |
|  | | **P to all 2.31** | | | |  | |  | | **2.163** | | **2.320** | | **1.901** | | **3.199** | |  |
| 128 | | 44 | |  | | I | | 0.372 | | 1.012 | | 0.956 | | 1.091 | | 0.943 | |  |
|  | |  | |  | | II | | 0.460 | | 0.925 | | 1.006 | | 1.048 | | 0.964 | |  |
|  | |  | |  | | med | | 0.416 | | 0.969 | | 0.981 | | 1.070 | | 0.954 | |  |
|  | | *P to all 2.39* | | | |  | |  | | *2.33* | | *2.36* | | *2.57* | | *2.29* | |  |
| 28 | | 45 | |  | | I | | 1.792 | | 0.947 | | 0.663 | | 0.453 | | 0.680 | |  |
|  | |  | |  | | II | | 1.946 | | 0.910 | | 0.722 | | 0.430 | | 0.726 | |  |
|  | |  | |  | | med | | 1.869 | | 0.929 | | 0.693 | | 0.442 | | 0.703 | |  |
|  | | **P to all 2.7** | | | |  | |  | | **2.013** | | **2.699** | | **4.233** | | **2.659** | |  |
| 489 | | 46 | |  | | I | | 1.156 | | 0.900 | | 0.839 | | 0.749 | | 0.764 | |  |
|  | |  | |  | | II | | 1.097 | | 0.928 | | 0.835 | | 0.748 | | 0.762 | |  |
|  | |  | |  | | med | | 1.127 | | 0.914 | | 0.837 | | 0.749 | | 0.763 | |  |
|  | | P to all 1.38 | | | |  | |  | | 1.232 | | 1.346 | | 1.505 | | 1.476 | |  |
| 19 | | 47 | |  | | I | | 0.227 | | 1.049 | | 0.960 | | 0.987 | | 1.172 | |  |
|  | |  | |  | | II | | 0.242 | | 1.065 | | 1.028 | | 0.941 | | 1.195 | |  |
|  | |  | |  | | med | | 0.235 | | 1.057 | | 0.994 | | 0.964 | | 1.184 | |  |
|  | | *P to all 4.48* | | | |  | |  | | *4.51* | | *4.24* | | *4.11* | | *5.05* | |  |
| 124 | | 48 | |  | | I | | 0.794 | | 1.883 | | 2.150 | | 1.859 | | 2.042 | |  |
|  | |  | |  | | II | | 0.780 | | 2.043 | | 1.984 | | 1.888 | | 2.104 | |  |
|  | |  | |  | | med | | 0.787 | | 1.963 | | 2.067 | | 1.874 | | 2.073 | |  |
|  | | *P to all 2.53* | | | |  | |  | | *2.49* | | *2.63* | | *2.38* | | *2.63* | |  |
| 173 | | 49 | |  | | I | | 1.708 | | 0.828 | | 0.718 | | 0.765 | | 0.802 | |  |
|  | |  | |  | | II | | 1.583 | | 0.811 | | 0.735 | | 0.730 | | 0.777 | |  |
|  | |  | |  | | med | | 1.646 | | 0.820 | | 0.727 | | 0.748 | | 0.790 | |  |
|  | | **P to all 2.13** | | | |  | |  | | **2.008** | | **2.265** | | **2.201** | | **2.084** | |  |
| 174 | | 50 | |  | | I | | 0.427 | | 0.930 | | 0.959 | | 0.766 | | 1.000 | |  |
|  | |  | |  | | II | | 0.480 | | 0.847 | | 1.009 | | 0.721 | | 1.075 | |  |
|  | |  | |  | | med | | 0.454 | | 0.889 | | 0.984 | | 0.744 | | 1.038 | |  |
|  | | *P to all 2.01* | | | |  | |  | | *1.96* | | *2.17* | | *1.64* | | *2.29* | |  |
| 163 | | 51 | |  | | I | | 0.580 | | 1.353 | | 1.273 | | 1.480 | | 1.401 | |  |
|  | |  | |  | | II | | 0.664 | | 1.306 | | 1.386 | | 1.411 | | 1.444 | |  |
|  | |  | |  | | med | | 0.622 | | 1.330 | | 1.330 | | 1.446 | | 1.423 | |  |
|  | | *P to all 2.22* | | | |  | |  | | *2.14* | | *2.14* | | *2.32* | | *2.29* | |  |
| 62 | | 52 | |  | | I | | 1.824 | | 0.530 | | 0.595 | | 0.802 | | 0.574 | |  |
|  | |  | |  | | II | | 1.750 | | 0.540 | | 0.602 | | 0.705 | | 0.562 | |  |
|  | |  | |  | | med | | 1.787 | | 0.535 | | 0.599 | | 0.754 | | 0.568 | |  |
|  | | **P to all 2.91** | | | |  | |  | | **3.340** | | **2.986** | | **2.372** | | **3.146** | |  |
| 169 | | 53 | |  | | I | | 1.655 | | 0.695 | | 0.772 | | 0.629 | | 0.625 | |  |
|  | |  | |  | | II | | 1.455 | | 0.664 | | 0.713 | | 0.774 | | 0.717 | |  |
|  | |  | |  | | med | | 1.555 | | 0.680 | | 0.743 | | 0.702 | | 0.671 | |  |
|  | | **P to all 2.23** | | | |  | |  | | **2.288** | | **2.094** | | **2.217** | | **2.317** | |  |
| 113 | | 54 | |  | | I | | 0.584 | | 1.306 | | 1.267 | | 1.450 | | 1.615 | |  |
|  | |  | |  | | II | | 0.607 | | 1.175 | | 1.449 | | 1.267 | | 1.586 | |  |
|  | |  | |  | | med | | 0.596 | | 1.241 | | 1.358 | | 1.359 | | 1.601 | |  |
|  | | *P to all 2.33* | | | |  | |  | | *2.08* | | *2.28* | | *2.28* | | *2.69* | |  |
| 229 | | 55 | |  | | I | | 0.445 | | 1.036 | | 0.815 | | 0.857 | | 0.967 | |  |
|  | |  | |  | | II | | 0.552 | | 0.925 | | 0.882 | | 0.788 | | 1.067 | |  |
|  | |  | |  | | med | | 0.499 | | 0.981 | | 0.849 | | 0.823 | | 1.017 | |  |
|  | | *P to all 1.84* | | | |  | |  | | *1.97* | | *1.70* | | *1.65* | | *2.04* | |  |
| 162 | | 56 | |  | | I | | 1.610 | | 1.065 | | 0.615 | | 0.694 | | 0.822 | |  |
|  | |  | |  | | II | | 1.560 | | 1.065 | | 0.757 | | 0.691 | | 0.969 | |  |
|  | |  | |  | |  | | 1.585 | | 1.065 | | 0.686 | | 0.693 | | 0.896 | |  |
|  | | **P to all 1.9** | | | | **med** | |  | | **1.488** | | **2.310** | | **2.289** | | **1.770** | |  |
| 56 | | 57 | |  | | I | | 1.483 | | 1.086 | | 0.898 | | 0.451 | | 1.001 | |  |
|  | |  | |  | | II | | 1.521 | | 1.059 | | 0.972 | | 0.419 | | 1.077 | |  |
|  | |  | |  | | med | | 1.502 | | 1.073 | | 0.935 | | 0.435 | | 1.039 | |  |
|  | | **P to all 1.73** | | | |  | |  | | **1.400** | | **1.606** | | **3.453** | | **1.446** | |  |
| 22 | | 58 | |  | | I | | 0.283 | | 0.954 | | 1.135 | | 1.088 | | 1.414 | |  |
|  | |  | |  | | II | | 0.333 | | 0.994 | | 1.175 | | 1.040 | | 1.440 | |  |
|  | |  | |  | | med | | 0.308 | | 0.974 | | 1.155 | | 1.064 | | 1.427 | |  |
|  | | *P to all 3.75* | | | |  | |  | | *3.16* | | *3.75* | | *3.45* | | *4.63* | |  |
| 178 | | 59 | |  | | I | | 0.400 | | 1.012 | | 0.888 | | 0.757 | | 0.996 | |  |
|  | |  | |  | | II | | 0.496 | | 0.949 | | 0.946 | | 0.690 | | 1.014 | |  |
|  | |  | |  | | med | | 0.448 | | 0.981 | | 0.917 | | 0.724 | | 1.005 | |  |
|  | | *P to all 2.02* | | | |  | |  | | *2.19* | | *2.05* | | *1.61* | | *2.24* | |  |
| 90 | | 60 | |  | | I | | 2.242 | | 0.987 | | 0.973 | | 0.744 | | 0.825 | |  |
|  | |  | |  | | II | | 2.187 | | 1.070 | | 0.983 | | 0.774 | | 0.931 | |  |
|  | |  | |  | | med | | 2.215 | | 1.029 | | 0.978 | | 0.759 | | 0.878 | |  |
|  | | **P to all** | | **2.43** | |  | |  | | **2.153** | | **2.264** | | **2.918** | | **2.522** | |  |
| 223 | | 61 | |  | | I | | 0.702 | | 1.286 | | 1.491 | | 1.354 | | 1.223 | |  |
|  | |  | |  | | II | | 0.739 | | 1.190 | | 1.478 | | 1.481 | | 1.215 | |  |
|  | |  | |  | | med | | 0.721 | | 1.238 | | 1.485 | | 1.418 | | 1.219 | |  |
|  | | *P to all 1.86* | | | |  | |  | | *1.72* | | *2.06* | | *1.97* | | *1.69* | |  |
| 96 | | 62 | |  | | I | | 1.664 | | 1.364 | | 1.112 | | 0.636 | | 1.215 | |  |
|  | |  | |  | | II | | 1.648 | | 1.202 | | 1.151 | | 0.545 | | 1.273 | |  |
|  | |  | |  | | med | | 1.656 | | 1.283 | | 1.132 | | 0.591 | | 1.244 | |  |
|  | | **P to all 1.56** | | | |  | |  | | **1.291** | | **1.464** | | **2.804** | | **1.331** | |  |
| 200 | | 63 | |  | | I | | 1.276 | | 1.160 | | 0.672 | | 0.557 | | 0.622 | |  |
|  | |  | |  | | II | | 1.197 | | 1.097 | | 0.624 | | 0.588 | | 0.695 | |  |
|  | |  | |  | | med | | 1.237 | | 1.129 | | 0.648 | | 0.573 | | 0.659 | |  |
|  | | **P to all 1.64** | | | |  | |  | | **1.096** | | **1.908** | | **2.160** | | **1.878** | |  |
| 292 | | 64 | |  | | I | | 0.542 | | 1.081 | | 0.934 | | 1.075 | | 0.995 | |  |
|  | |  | |  | | II | | 0.598 | | 1.030 | | 1.009 | | 1.059 | | 1.037 | |  |
|  | |  | |  | | med | | 0.570 | | 1.056 | | 0.972 | | 1.067 | | 1.016 | |  |
|  | | *P to all 1.8* | | | |  | |  | | *1.85* | | *1.70* | | *1.87* | | *1.78* | |  |
| 281 | | 65 | |  | | I | | 1.167 | | 0.862 | | 0.784 | | 0.621 | | 0.815 | |  |
|  | |  | |  | | II | | 1.140 | | 0.822 | | 0.756 | | 0.599 | | 0.811 | |  |
|  | |  | |  | | med | | 1.154 | | 0.842 | | 0.770 | | 0.610 | | 0.813 | |  |
|  | | **P to all 1.52** | | | |  | |  | | **1.370** | | **1.498** | | **1.891** | | **1.419** | |  |
| 1 | | 66 | |  | | I | | 0.122 | | 1.056 | | 1.025 | | 1.011 | | 1.336 | |  |
|  | |  | |  | | II | | 0.119 | | 1.095 | | 1.060 | | 0.884 | | 1.217 | |  |
|  | |  | |  | | med | | 0.121 | | 1.076 | | 1.043 | | 0.948 | | 1.277 | |  |
|  | | *P to all 9.01* | | | |  | |  | | *8.93* | | *8.65* | | *7.86* | | *10.59* | |  |
| 164 | | 67 | |  | | I | | 0.592 | | 1.409 | | 1.221 | | 1.042 | | 1.427 | |  |
|  | |  | |  | | II | | 0.667 | | 1.355 | | 1.307 | | 0.958 | | 1.506 | |  |
|  | |  | |  | | med | | 0.630 | | 1.382 | | 1.264 | | 1.000 | | 1.467 | |  |
|  | | *P to all 2.03* | | | |  | |  | | *2.20* | | *2.01* | | *1.59* | | *2.33* | |  |
| 165 | | 68 | |  | | I | | 1.374 | | 0.711 | | 0.599 | | 0.677 | | 0.610 | |  |
|  | |  | |  | | II | | 1.432 | | 0.683 | | 0.621 | | 0.645 | | 0.698 | |  |
|  | |  | |  | | med | | 1.403 | | 0.697 | | 0.610 | | 0.661 | | 0.654 | |  |
|  | | **P to all 2.14** | | | |  | |  | | **2.013** | | **2.300** | | **2.123** | | **2.145** | |  |
| 199 | | 69 | |  | | I | | 0.400 | | 0.874 | | 0.854 | | 0.887 | | 0.878 | |  |
|  | |  | |  | | II | | 0.425 | | 0.820 | | 0.897 | | 0.898 | | 0.804 | |  |
|  | |  | |  | | med | | 0.413 | | 0.847 | | 0.876 | | 0.893 | | 0.841 | |  |
|  | | *P to all 2.09* | | | |  | |  | | *2.05* | | *2.12* | | *2.16* | | *2.04* | |  |
| 121 | | 70 | |  | | I | | 0.458 | | 1.200 | | 0.919 | | 0.775 | | 1.149 | |  |
|  | |  | |  | | II | | 0.409 | | 1.087 | | 1.037 | | 0.907 | | 0.975 | |  |
|  | |  | |  | | med | | 0.434 | | 1.144 | | 0.978 | | 0.841 | | 1.062 | |  |
|  | | *P to all 2.32* | | | |  | |  | | *2.64* | | *2.26* | | *1.94* | | *2.45* | |  |
| 147 | | 71 | |  | | I | | 2.183 | | 0.868 | | 0.964 | | 1.137 | | 1.156 | |  |
|  | |  | |  | | II | | 1.962 | | 0.862 | | 1.067 | | 1.030 | | 1.124 | |  |
|  | |  | |  | | med | | 2.073 | | 0.865 | | 1.016 | | 1.084 | | 1.140 | |  |
|  | | **P to all 2.02** | | | |  | |  | | **2.396** | | **2.041** | | **1.913** | | **1.818** | |  |
| 479 | | 72 | |  | | I | | 0.779 | | 0.979 | | 1.117 | | 0.955 | | 1.168 | |  |
|  | |  | |  | | II | | 0.736 | | 1.034 | | 1.078 | | 0.990 | | 1.140 | |  |
|  | |  | |  | | med | | 0.758 | | 1.007 | | 1.098 | | 0.973 | | 1.154 | |  |
|  | | P to all | | 1.4 | |  | |  | | 1.33 | | 1.45 | | 1.28 | | 1.52 | |  |
| 106 | | 73 | |  | | I | | 1.445 | | 0.722 | | 0.934 | | 0.939 | | 0.523 | |  |
|  | |  | |  | | II | | 1.514 | | 0.673 | | 1.047 | | 0.877 | | 0.567 | |  |
|  | |  | |  | | med | | 1.480 | | 0.698 | | 0.991 | | 0.908 | | 0.545 | |  |
|  | | **P to all 1.88** | | | |  | |  | | **2.121** | | **1.494** | | **1.629** | | **2.715** | |  |
| 327 | | 74 | |  | | I | | 0.635 | | 1.082 | | 1.072 | | 1.215 | | 0.993 | |  |
|  | |  | |  | | II | | 0.672 | | 1.038 | | 1.122 | | 1.132 | | 1.055 | |  |
|  | |  | |  | | med | | 0.654 | | 1.060 | | 1.097 | | 1.174 | | 1.024 | |  |
|  | | *P to all 1.67* | | | |  | |  | | *1.62* | | *1.68* | | *1.80* | | *1.57* | |  |
| 260 | | 75 | |  | | I | | 0.947 | | 1.176 | | 1.090 | | 0.616 | | 1.233 | |  |
|  | |  | |  | | II | | 0.886 | | 1.172 | | 1.091 | | 0.640 | | 1.228 | |  |
|  | |  | |  | | med | | 0.917 | | 1.174 | | 1.091 | | 0.628 | | 1.231 | |  |
|  | | P to all 1.12 | | | |  | |  | | 1.28 | | 1.19 | | 0.69 | | 1.34 | |  |
| 118 | | 76 | |  | | I | | 1.171 | | 0.610 | | 0.876 | | 0.870 | | 0.458 | |  |
|  | |  | |  | | II | | 1.198 | | 0.608 | | 0.886 | | 0.836 | | 0.439 | |  |
|  | |  | |  | | med | | 1.185 | | 0.609 | | 0.881 | | 0.853 | | 0.449 | |  |
|  | | **P to all 1.7** | | | |  | |  | | **1.945** | | **1.344** | | **1.389** | | **2.641** | |  |
| 537 | | 77 | |  | | I | | 0.691 | | 0.873 | | 0.949 | | 1.012 | | 0.999 | |  |
|  | |  | |  | | II | | 0.713 | | 0.870 | | 0.967 | | 0.996 | | 0.967 | |  |
|  | |  | |  | | med | | 0.702 | | 0.872 | | 0.958 | | 1.004 | | 0.983 | |  |
|  | | P to all 1.36 | | | |  | |  | | 1.24 | | 1.36 | | 1.43 | | 1.40 | |  |
| 74 | | 78 | |  | | I | | 1.455 | | 0.729 | | 1.005 | | 0.858 | | 0.460 | |  |
|  | |  | |  | | II | | 1.404 | | 0.717 | | 0.968 | | 0.860 | | 0.448 | |  |
|  | |  | |  | | med | | 1.430 | | 0.723 | | 0.987 | | 0.859 | | 0.454 | |  |
|  | | **P to all 1.89** | | | |  | |  | | **1.977** | | **1.449** | | **1.664** | | **3.149** | |  |
| 132 | | 79 | |  | | I | | 0.580 | | 1.192 | | 1.275 | | 1.264 | | 1.464 | |  |
|  | |  | |  | | II | | 0.645 | | 1.178 | | 1.316 | | 1.242 | | 1.658 | |  |
|  | |  | |  | | med | | 0.613 | | 1.185 | | 1.296 | | 1.253 | | 1.561 | |  |
|  | | *P to all 2.16* | | | |  | |  | | *1.93* | | *2.12* | | *2.05* | | *2.55* | |  |
| 406 | | 80 | |  | | I | | 0.925 | | 1.380 | | 1.210 | | 1.300 | | 1.357 | |  |
|  | |  | |  | | II | | 0.893 | | 1.301 | | 1.304 | | 1.171 | | 1.637 | |  |
|  | |  | |  | | med | | 0.909 | | 1.341 | | 1.257 | | 1.236 | | 1.497 | |  |
|  | | P to all 1.47 | | | |  | |  | | 1.47 | | 1.38 | | 1.36 | | 1.65 | |  |
| 145 | | 81 | |  | | I | | 0.441 | | 1.083 | | 0.971 | | 0.791 | | 1.078 | |  |
|  | |  | |  | | II | | 0.444 | | 1.048 | | 0.987 | | 0.778 | | 1.045 | |  |
|  | |  | |  | | med | | 0.443 | | 1.066 | | 0.979 | | 0.785 | | 1.062 | |  |
|  | | *P to all 2.2* | | | |  | |  | | *2.41* | | *2.21* | | *1.77* | | *2.40* | |  |
| 101 | | 82 | |  | | I | | 1.513 | | 0.786 | | 1.066 | | 1.040 | | 0.556 | |  |
|  | |  | |  | | II | | 1.473 | | 0.778 | | 1.014 | | 0.980 | | 0.528 | |  |
|  | |  | |  | | med | | 1.493 | | 0.782 | | 1.040 | | 1.010 | | 0.542 | |  |
|  | | **P to all 1.77** | | | |  | |  | | **1.909** | | **1.436** | | **1.478** | | **2.755** | |  |
| 102 | | 83 | |  | | I | | 0.475 | | 0.920 | | 1.179 | | 1.067 | | 1.300 | |  |
|  | |  | |  | | II | | 0.444 | | 1.033 | | 1.117 | | 1.081 | | 1.252 | |  |
|  | |  | |  | | med | | 0.460 | | 0.977 | | 1.148 | | 1.074 | | 1.276 | |  |
|  | | *P to all 2.43* | | | |  | |  | | *2.13* | | *2.50* | | *2.34* | | *2.78* | |  |
| 421 | | 84 | |  | | I | | 0.765 | | 1.237 | | 0.951 | | 1.257 | | 0.965 | |  |
|  | |  | |  | | II | | 0.763 | | 1.214 | | 0.965 | | 1.214 | | 1.031 | |  |
|  | |  | |  | | med | | 0.764 | | 1.226 | | 0.958 | | 1.236 | | 0.998 | |  |
|  | | P to all 1.45 | | | |  | |  | | 1.60 | | 1.25 | | 1.62 | | 1.31 | |  |
| 184 | | 85 | |  | | I | | 1.389 | | 0.723 | | 1.003 | | 0.974 | | 0.631 | |  |
|  | |  | |  | | II | | 1.370 | | 0.735 | | 1.031 | | 0.938 | | 0.622 | |  |
|  | |  | |  | | med | | 1.380 | | 0.729 | | 1.017 | | 0.956 | | 0.627 | |  |
|  | | **P to all 1.66** | | | |  | |  | | **1.892** | | **1.356** | | **1.443** | | **2.202** | |  |
| 387 | | 86 | |  | | I | | 0.618 | | 0.625 | | 0.903 | | 1.075 | | 0.853 | |  |
|  | |  | |  | | II | | 0.628 | | 0.633 | | 0.958 | | 1.018 | | 0.847 | |  |
|  | |  | |  | | med | | 0.623 | | 0.629 | | 0.931 | | 1.047 | | 0.850 | |  |
|  | | P to all 1.38 | | | |  | |  | | 1.01 | | 1.49 | | 1.68 | | 1.36 | |  |
| 491 | | 87 | |  | | I | | 0.674 | | 0.984 | | 0.899 | | 0.746 | | 0.989 | |  |
|  | |  | |  | | II | | 0.643 | | 0.998 | | 0.871 | | 0.751 | | 0.956 | |  |
|  | |  | |  | | med | | 0.659 | | 0.991 | | 0.885 | | 0.749 | | 0.973 | |  |
|  | | P to all 1.37 | | | |  | |  | | 1.50 | | 1.34 | | 1.14 | | 1.48 | |  |
| 251 | | 88 | |  | | I | | 0.791 | | 1.244 | | 1.432 | | 1.154 | | 1.201 | |  |
|  | |  | |  | | II | | 0.710 | | 1.388 | | 1.554 | | 1.263 | | 1.173 | |  |
|  | |  | |  | | med | | 0.751 | | 1.316 | | 1.493 | | 1.209 | | 1.187 | |  |
|  | | *P to all 1.73* | | | |  | |  | | *1.75* | | *1.99* | | *1.61* | | *1.58* | |  |
| 91 | | 89 | |  | | I | | 1.708 | | 0.925 | | 1.318 | | 1.170 | | 0.571 | |  |
|  | |  | |  | | II | | 1.595 | | 0.951 | | 1.320 | | 1.115 | | 0.571 | |  |
|  | |  | |  | | med | | 1.652 | | 0.938 | | 1.319 | | 1.143 | | 0.571 | |  |
|  | | **P to all 1.66** | | | |  | |  | | **1.761** | | **1.252** | | **1.446** | | **2.892** | |  |
| 283 | | 90 | |  | | I | | 1.330 | | 0.730 | | 1.019 | | 1.021 | | 0.718 | | |
|  | |  | |  | | II | | 1.297 | | 0.759 | | 1.047 | | 1.024 | | 0.679 | | |
|  | |  | |  | | med | | 1.314 | | 0.745 | | 1.033 | | 1.023 | | 0.699 | | |
|  | | **P to all 1.5** | | | |  | |  | | **1.764** | | **1.272** | | **1.285** | | **1.880** | | |
| 487 | | 91 | |  | | I | | 1.669 | | 1.716 | | 1.495 | | 1.109 | | 1.591 | | |
|  | |  | |  | | II | | 1.499 | | 1.808 | | 1.472 | | 1.166 | | 1.508 | | |
|  | |  | |  | | med | | 1.584 | | 1.762 | | 1.484 | | 1.138 | | 1.550 | | |
|  | | P to all 1.07 | | | |  | |  | | 0.899 | | 1.068 | | 1.393 | | 1.022 | | |
| 263 | | 92 | |  | | I | | 0.764 | | 1.300 | | 1.445 | | 1.266 | | 1.361 | | |
|  | |  | |  | | II | | 0.739 | | 1.287 | | 1.452 | | 1.208 | | 1.419 | | |
|  | |  | |  | | med | | 0.752 | | 1.294 | | 1.449 | | 1.237 | | 1.390 | | |
|  | | *P to all 1.79* | | | |  | |  | | *1.72* | | *1.93* | | *1.65* | | *1.85* | | |
| 477 | | 93 | |  | | I | | 1.460 | | 1.557 | | 1.102 | | 1.250 | | 1.463 | | |
|  | |  | |  | | II | | 1.324 | | 1.726 | | 1.071 | | 1.263 | | 1.463 | | |
|  | |  | |  | | med | | 1.392 | | 1.642 | | 1.087 | | 1.257 | | 1.463 | | |
|  | | P to all 0.98 | | | |  | |  | | 1.18 | | 0.78 | | 0.90 | | 1.05 | | |
| 663 | | 94 | |  | | I | | 1.129 | | 1.268 | | 1.352 | | 1.170 | | 1.160 | | |
|  | |  | |  | | II | | 1.061 | | 1.294 | | 1.326 | | 1.179 | | 1.152 | | |
|  | |  | |  | | med | | 1.095 | | 1.281 | | 1.339 | | 1.175 | | 1.156 | | |
|  | | P to all 1.13 | | | |  | |  | | 1.17 | | 1.22 | | 1.07 | | 1.06 | | |
| 631 | | 95 | |  | | I | | 1.089 | | 1.124 | | 1.346 | | 1.292 | | 1.362 | | |
|  | |  | |  | | II | | 0.991 | | 1.172 | | 1.330 | | 1.338 | | 1.340 | | |
|  | |  | |  | | med | | 1.040 | | 1.148 | | 1.338 | | 1.315 | | 1.351 | | |
|  | | P to all 1.24 | | | |  | |  | | 1.10 | | 1.29 | | 1.26 | | 1.30 | | |
| 416 | | 96 | |  | | I | | 0.675 | | 0.691 | | 0.888 | | 1.088 | | 0.913 | | |
|  | |  | |  | | II | | 0.689 | | 0.680 | | 0.883 | | 1.119 | | 0.948 | | |
|  | |  | |  | | med | | 0.682 | | 0.686 | | 0.886 | | 1.104 | | 0.931 | | |
|  | | P to all 1.32 | | | |  | |  | | 1.01 | | 1.30 | | 1.62 | | 1.36 | | |

Spot rank: Rank of spot as assigned by Same Spots Software depending on fold change (normalized volume) comparing the highest to lowest sample

Pick #: Sequence in which spots were excised from gel depending on spot intensity (from lowest to highest)

Roman Numerals: Normalized volume measured in replicate runs one (I) and two (II)

Med: Average of normalized volume measured in replicate runs one (I) and two (II)

Fold Change P to all: Expression level (normalized volume) of patient sample compared to the average of all other samples combined;

Last row of columns also shows fold change comparing patient to sample indicated in header of column (Mother, Father, Control, Standard)

Formatting: Bold: Patient upregulated Italic: Patient downregulated

No special formatting: Fold change “P to all” does not exceed 1.5 fold
