# Supplementary material for: Assessment of the Red Cell Proteome of Young Patients with Unexplained Hemolytic Anemia by Two-Dimensional Differential In-Gel Electrophoresis (DIGE)
Source: PLoS One. 2012 Apr 3;7(4):e34237. doi: 10.1371/journal.pone.0034237 (PMC3317954; doi:10.1371/journal.pone.0034237)
Supplement: Table S14 — Complete list of all spots picked and Proteins identified in HA24. (DOCX) [file pone.0034237.s018.docx]

# Table S14: Complete list of all spots picked and Proteins identified in HA24

| **Spot rank** | **Pick #** | **Spot Location**  **(kD, pI)** | **Coverage**  **(%)** | ***Gene*** | **Protein** | SC |
| --- | --- | --- | --- | --- | --- | --- |
| 7 | 1 | 100 / 7.0 |  |  | Like spot #4 elongation factor 2 (NO ID) |  |
| 22 | 2 | 22 / 8.5 | 25 | *RAB10* | Ras-related protein Rab-10 (22.5kD, pI 8.4) | 5 |
| 14 | 3 | 110 / 6.8 | 22.4 | *XPO7* | Exportin 7 isoform a (125kD, pI 6.3) | 34 |
|  |  |  | 9.5 | *ALAD* | Delta-aminolevulinic acid dehydratase isoform a (39kD, pI 7.6) | 5 |
| 4 | 4 | 95 / 7.5 | 7.2 | *EEF2* | Elongation factor 2 (95kD, pI 6.8) | 6 |
| 41 | 5 | 50 / 7.0 | 36.3 | *PIP4K2A* | Phosphatidylinositol-5-phosphate 4-kinase type-2 alpha | 23 |
|  |  |  |  |  | (46kD, pI 7.0) |  |
| 55 | 6 | 110 / 6.8 | 21.9 | *XPO7* | Exportin 7 isoform a (125kD, pI 6.3) | 30 |
|  |  |  | 12.3 | *ALAD* | Delta-aminolevulinic acid dehydratase isoform a (39kD, pI 7.6) | 5 |
|  |  |  | 4.4 | *TPP2* | Tripeptidyl peptidase II (140kD, pI 6.5) | 3 |
| 52 | 7 | 90 / 6.3 | 23.8 | *ALB* | Uncharacterized protein ALB (71kD, pI 6.7) | 13 |
|  |  |  | 16.2 | *GSN* | Isoform 1 of Gelsolin precursor (85.6kD, pI 6.3) | 11 |
|  |  |  | 6.8 | *TGM2* | Isoform 1 of Protein-glutamine gamma-glutamyltransferase 2 | 5 |
|  |  |  |  |  | (77.3kD, pI 5.2) |  |
|  |  |  | 5.4 | *HSP90AA1* | Heat shock protein 90kDa alpha (cytosolic), class A member 1 | 5 |
|  |  |  |  |  | isoform 1 (98kD, pI 5.2) |  |
|  |  |  | 5.3 | *STAT5B* | Signal transducer and activator of transcription 5B (89.8kD, pI 6.1) | 4 |
| 60 | 8 | 75 / 7.0 | 20.3 | *ALAD* | Delta-aminolevulinic acid dehydratase isoform a (39kD, pI 7.6) | 16 |
|  |  |  | 20 | *NSF* | N-ethylmaleimide-sensitive factor / Vesicle-fusing ATPase | 15 |
|  |  |  |  |  | (82.5kD, pI 7.0) |  |
| 1 | 9 | 60 / 7.0 | 13.1 | *PRDX2* | Peroxiredoxin-2 (21.8kD, pI 6.0) | 20 |
|  |  |  | 7.6 | *FBXO7* | F-box only protein 7 (58kD, pI 6.8) | 8 |
| 50 | 10 | 58 / 6.8 |  |  | NO ID |  |
| 76 | 11 | 25 / 5.5 | 4.4 | *ALDH1A1* | Aldehyde dehydrogenase 1 family, member A1 (55kD, pI 6.7) | 2 |
| 132 | 12 | 52 / 7.0 | 34.1 | *ADSL* | Isoform 1 of Adenylosuccinate lyase (55kD, pI 7.1) | 25 |
|  |  |  | 6.8 | *RUVBL1* | RuvB-like 1 (E. coli) (50.2kD, pI 6.4) | 2 |
|  |  |  | 5.4 | *PIP4K2A* | Phosphatidylinositol-5-phosphate 4-kinase type-2 alpha | 3 |
|  |  |  |  |  | (46kD, pI 7.0) |  |
| 13 | 13 | 45 / 5.0 | 22.3 | *RPSA* | Ribosomal protein SA (33.3kD, pI 4.9) | 17 |
| 20 | 14 | 70 / 6.2 | 43.1 | *ALB* | Uncharacterized protein ALB (71kD, pI 6.7) | 60 |
|  |  |  | 10.8 | *ALDH1A1* | Aldehyde dehydrogenase 1 family, member A1 (55kD, pI 6.7) | 4 |
| 62 | 15 | 27 / 6.5 | 24.6 | *TSN* | Translin (26.1kD, pI 6.4) | 10 |
|  |  |  | 23.9 | *HPRT1* | Hypoxanthine phosphoribosyltransferase 1 (24.6kD, pI 7.0) | 10 |
|  |  |  | 17.4 | *PSMB4* | Proteasome subunit beta type-4 precursor (29.2kD, pI 6.0) | 5 |
| 94 | 16 | 40 / 6.5 | 30.1 | *PURA* | Transcriptional activator protein Pur-alpha (34.9kD, pI 6.4) | 34 |
|  |  |  | 16.7 | *MAPK1* | Mitogen-activated protein kinase 1 (41.4kD, pI 7.0) | 4 |
| 88 | 17 | 37 / 8.0 | 38.3 | *GMPR* | GMP reductase 1 (37.4kD, pI 7.1) | 40 |
|  |  |  | 17.1 | *GMPR2* | Guanosine monophosphate reductase 2 (45kD, pI 8.3) | 6 |
| 64 | 18 | 55 / 7.5 | 27.8 | *CAP1* | Adenylyl cyclase-associated protein 1 (51.9kD, pI 8.1) | 20 |
|  |  |  | 11.9 | *GMPR* | GMP reductase 1 (37.4kD, pI 7.1) | 3 |
| 75 | 19 | 35 / 5.0 | 51.9 | *NAPA* | Alpha-soluble NSF attachment protein (33.2kD, pI 5.4) | 41 |
|  |  |  | 13.9 | *GMPR* | GMP reductase 1 (37.4kD, pI 7.1) | 2 |
|  |  |  | 5.9 | *BAG1* | Isoform 1 of BAG family molecular chaperone regulator 1 | 3 |
|  |  |  |  |  | (38.8kD, pI 8.1) |  |
| 36 | 20 | 30 / 6.5 | 23.4 | *NAPA* | Alpha-soluble NSF attachment protein (33.2kD, pI 5.4) carry over | 8 |
|  |  |  | 19.3 | *BPGM* | Bisphosphoglycerate mutase (30kD, pI 6.5) | 6 |
| 38 | 21 | 25 / 5.8 | 46.7 | *GSTM3* | Glutathione S-transferase mu 3 (brain) (26.5kD, pI 5.5) | 30 |
|  |  |  | 34.1 | *PITHD1* | PITH domain-containing protein 1 (24kD, pI 5.8) | 7 |
|  |  |  | 17.4 | *PSMB4* | Proteasome subunit beta type-4 precursor (29.2kD, pI 6.0) | 3 |
|  |  |  | 16 | *APOA1BP* | Isoform 1 of Apolipoprotein A-I-binding protein precursor | 5 |
|  |  |  |  |  | (31.6kD, pI 7.7) |  |
| 201 | 22 | 40 / 6.5 | 40.1 | *COPS5* | COP9 signalosome complex subunit 5 (37.6kD, pI 6.5) | 24 |
|  |  |  | 29.3 | *GMPR* | GMP reductase 1 (37.4kD, pI 7.1) | 9 |
|  |  |  | 14.9 | *NIF3L1* | Putative uncharacterized protein NIF3L1 (42kD, pI 6.7) | 5 |
|  |  |  | 7.6 | *GSTM3* | Glutathione S-transferase mu 3 (brain) (26.5kD, pI 5.5) | 2 |
| 167 | 23 | 28 / 7.0 | 53.9 | *PGAM1* | Phosphoglycerate mutase 1 (brain) (28.8kD, pI 7.2) | 33 |
|  |  |  | 27.4 | *PSMA7* | Isoform 1 of Proteasome subunit alpha type-7 (28kD, pI 8.5) | 6 |
|  |  |  | 3.9 | *PSMD8* | Proteasome 26S non-ATPase subunit 8 (39.6kD, pI 9.7) | 3 |
| 81 | 24 | 60 / 6.5 | 27.2 | *PGAM1* | Phosphoglycerate mutase 1 (brain) (28.8kD, pI 7.2) | 10 |
|  |  |  | 11.4 | *COPS5* | COP9 signalosome complex subunit 5 (37.6kD, pI 6.5) | 2 |
| 138 | 25 | 45 / 5.0 | 41.7 | *TSTA3* | GDP-L-fucose synthetase (35.8kD, pI 6.6) | 28 |
|  |  |  | 25.1 | *FDPS* | Farnesyl diphosphate synthase (48.2kD, pI 6.2) | 14 |
| 285 | 26 | 40 / 7.0 | 42.7 | *TSTA3* | GDP-L-fucose synthetase (35.8kD, pI 6.6) | 54 |
|  |  |  | 11.1 | *CCT8* | Chaperonin containing TCP1, subunit 8 (theta) (59.7kD, pI 5.6) | 7 |
| 126 | 27 | 55 / 6.0 | 34.5 | *CCT8* | Chaperonin containing TCP1, subunit 8 (theta) (59.7kD, pI 5.6) | 24 |
|  |  |  | 33.7 | *PRDX1* | Peroxiredoxin-1 (22.1kD, pI 8.1) | 8 |
|  |  |  | 12.1 | *USP14* | Ubiquitin carboxyl-terminal hydrolase 14 (56kD, pI 5.3) | 6 |
|  |  |  | 11.7 | *PSMA7* | Isoform 1 of Proteasome subunit alpha type-7 (28kD, pI 8.5) | 2 |
|  |  |  | 7.8 | *TSTA3* | GDP-L-fucose synthetase (35.8kD, pI 6.6) | 2 |
|  |  |  | 5.6 | *EIF5A* | Isoform 2 of Eukaryotic translation initiation factor 5A-1 | 3 |
|  |  |  |  |  | (20.1kD, pI 7.0) |  |
|  |  |  | 4.5 | *FBXO7* | F-box only protein 7 (58kD, pI 6.8) | 4 |
| 49 | 28 | 22 / 8.2 | 58 | *PNP* | Purine Nucleosidee Phosphorylase (33kD, pI 7.2) | 30 |
|  |  |  | 47.7 | *PRDX1* | Peroxiredoxin-1 (22.1kD, pI 8.1) | 11 |
|  |  |  | 32.7 | *PSMD7* | 26S proteasome non-ATPase regulatory subunit 7 (37kD, pI 6.8) | 16 |
|  |  |  | 26.4 | *GMPR* | GMP reductase 1 (37.4kD, pI 7.1) | 10 |
|  |  |  | 15 | *PSMB5* | Proteasome subunit, beta type, 5 (28kD, pI 6.5) | 6 |
|  |  |  | 11.7 | *PSMA7* | Isoform 1 of Proteasome subunit alpha type-7 (28kD, pI 8.5) | 2 |
|  |  |  | 6.2 | *CCT8* | Chaperonin containing TCP1, subunit 8 (theta) (59.7kD, pI 5.6) | 2 |
| 56 | 29 | 30 / 6.5 | 67.9 | *PNP* | Purine Nucleoside Phosphorylase (33kD, pI 7.2) | 52 |
|  |  |  | 19.3 | *PSMD12* | 26S proteasome non-ATPase regulatory subunit 12 | 9 |
|  |  |  |  |  | (52.9kD, pI 7.6) |  |
| 173 | 30 | 50 / 8.0 | 41.4 | *PSMD12* | 26S proteasome non-ATPase regulatory subunit 12 | 40 |
|  |  |  |  |  | (52.9kD, pI 7.6) |  |
|  |  |  | 29.3 | *GMPR* | GMP reductase 1 (37.4kD, pI 7.1) | 10 |
|  |  |  | 25.9 | *PNP* | Purine Nucleoside Phosphorylase (33kD, pI 7.2) | 4 |
|  |  |  | 6.9 | *AP2M1* | AP-2 complex subunit mu-1 (59.6kD, pI 9.5) | 3 |
| 111 | 31 | 37 / 7.5 | 36.8 | *GMPR* | GMP reductase 1 (37.4kD, pI 7.1) | 34 |
|  |  |  | 13.1 | *PSMD8* | Proteasome 26S non-ATPase subunit 8 (39.6kD, pI 9.7) | 9 |
|  |  |  | 9.9 | *PSMD7* | 26S proteasome non-ATPase regulatory subunit 7 (37kD, pI 6.8) | 2 |
| 179 | 32 | 30 / 7.0 | 41.9 | *PSMC5* | 26S protease regulatory subunit 8 (45.6kD, pI 7.5) | 32 |
|  |  |  | 26.6 | *PSMD8* | Proteasome 26S non-ATPase subunit 8 (39.6kD, pI 9.7) | 24 |
|  |  |  | 20.3 | *GMPR* | GMP reductase 1 (37.4kD, pI 7.1) | 4 |
|  |  |  | 12.6 | *PSMD9* | Isoform p27-L of 26S proteasome non-ATPase regulatory SU 9 | 4 |
|  |  |  |  |  | (24.6kD, pI 7.0) |  |
| 210 | 33 | 45 / 8.0 | 51.5 | *NAPA* | Alpha-soluble NSF attachment protein (33.2kD, pI 5.4) | 18 |
|  |  |  | 41.9 | *PSMC5* | 26S protease regulatory subunit 8 (45.6kD, pI 7.5) | 56 |
| 140 | 34 | 33 / 5.8 | 61.7 | *NAPA* | Alpha-soluble NSF attachment protein (33.2kD, pI 5.4) | 33 |
|  |  |  | 58 | *PNP* | Purine Nucleoside Phosphorylase (33kD, pI 7.2) | 42 |
|  |  |  | 5.6 | *NAPRT1* | Nicotinate phosphoribosyltransferase domain containing 1 | 2 |
|  |  |  |  |  | (60.2kD, pI 5.8) |  |
| 47 | 35 | 33 / 6.8 | 65.3 | *PNP* | Purine Nucleoside Phosphorylase (33kD, pI 7.2) | 73 |
|  |  |  | 27.8 | *ASNA1* | ATPase ASNA1 (40.2kD, pI 5.1) | 12 |
|  |  |  | 5.6 | *PSMG1* | Proteasome assembly chaperone 1 (32.8kD, pI 7.2) | 2 |
| 80 | 36 | 40 / 5.0 | 46.8 | *ASNA1* | ATPase ASNA1 (40.2kD, pI 5.1) | 31 |
|  |  |  | 37 | *GLRX3* | Glutaredoxin-3 (37.4kD, pI 5.4) | 16 |
|  |  |  | 24.9 | *PNP* | Purine Nucleoside Phosphorylase (33kD, pI 7.2) | 10 |
|  |  |  | 18.4 | *SUGT1* | Isoform 1 of Suppressor of G2 allele of SKP1 homolog | 5 |
|  |  |  |  |  | (41kD, pI 5.2) |  |
|  |  |  | 10.6 | *CRKL* | V-crk sarcoma virus CT10 oncogene homolog (avian)-like | 2 |
|  |  |  |  |  | (33.7kD, pI 6.7) |  |
| 122 | 37 | 40 / 5.8 | 33.6 | *CCT2* | Chaperonin containing TCP1, subunit 2 (beta) (57.5kD, pI 6.4) | 27 |
|  |  |  | 28.1 | *GLRX3* | Glutaredoxin-3 (37.4kD, pI 5.4) | 14 |
|  |  |  | 9.4 | *PPP2CA/ PPP2CB* | Serine/threonine-protein phosphatase 2A catalytic subunit  Alpha or beta isoform (35.5kD, pI 5.5 or 5.4) | 2 |
|  |  |  | 8 | *ASNA1* | ATPase ASNA1 (40.2kD, pI 5.1) | 2 |
| 145 | 38 | 52 / 6.8 | 41.3 | *CCT2* | Chaperonin containing TCP1, subunit 2 (beta) (57.5kD, pI 6.4) | 52 |
|  |  |  | 32.7 | *PSMD7* | 26S proteasome non-ATPase regulatory subunit 7 (37kD, pI 6.8) | 17 |
|  |  |  | 23.8 | *GMPR* | GMP reductase 1 (37.4kD, pI 7.1) | 11 |
|  |  |  | 13.4 | *DARS* | Aspartyl-tRNA synthetase, cytoplasmic (57kD, pI 6.5) | 7 |
| 305 | 39 | 37 / 7.5 | 42 | *PSMD7* | 26S proteasome non-ATPase regulatory subunit 7 (37kD, pI 6.8) | 42 |
|  |  |  | 33 | *GMPR* | GMP reductase 1 (37.4kD, pI 7.1) | 24 |
|  |  |  | 26.3 | *BPGM* | Bisphosphoglycerate mutase (30kD, pI 6.5) | 11 |
|  |  |  | 21.5 | *CCT2* | Chaperonin containing TCP1, subunit 2 (beta) | 8 |
|  |  |  |  |  | (57.5kD, pI 6.4) carry over |  |
|  |  |  | 7.3 | *GMPR2* | Guanosine monophosphate reductase 2 (45kD, pI 8.3) | 3 |
| 43 | 40 | 30 / 7.5 | 23.4 | *FDPS* | Farnesyl diphosphate synthase (48kD, pI 6.2) | 11 |
|  |  |  | 22 | *BPGM* | Bisphosphoglycerate mutase (30kD, pI 6.5) | 30 |
|  |  |  | 12.6 | *PSMD9* | Isoform p27-L of 26S proteasome non-ATPase | 4 |
|  |  |  |  |  | Regulatory subunit 9 (24.6kD, pI 7.0) |  |
|  |  |  | 11.9 | *GMPR* | GMP reductase 1 (37.4kD, pI 7.1) carry over | 3 |
|  |  |  | 9.9 | *PSMD7* | 26S proteasome non-ATPase regulatory subunit 7 (37kD, pI 6.8) | 3 |
|  |  |  | 6.2 | *SNF8* | Isoform 1 of Vacuolar-sorting protein SNF8 (28.8kD, pI 6.8), | 3 |
| 165 | 41 | 55 / 8.0 | 56.7 | *CCT7* | T-complex protein 1 subunit eta (59.4kD, pI 7.6) | 101 |
|  |  |  | 31.9 | *CCT4* | T-complex protein 1 subunit delta (57.9kD, pI 7.8) | 19 |
|  |  |  | 12.4 | *BPGM* | Bisphosphoglycerate mutase (30kD, pI 6.5) carry over | 2 |
| 284 | 42 | 28 / 8.0 | 57.5 | *PSMA4* | Proteasome subunit alpha type-4 (29.4kD, pI 7.7) | 69 |
|  |  |  | 11.7 | *PSMA7* | Isoform 1 of Proteasome subunit alpha type-7 (28kD, pI 8.5) | 2 |
|  |  |  | 8.1 | *CCT7* | T-complex protein 1 subunit eta (59.4kD, pI 7.6) carry over | 6 |
| 23 | 43 | 30 / 6.8 | 73.7 | *PNP* | Purine Nucleoside Phosphorylase (33kD, pI 7.2) | 78 |
|  |  |  | 21.5 | *PSMA4* | Proteasome subunit alpha type-4 (29.4kD, pI 7.7) | 6 |
| 97 | 44 | 58 / 6.0 | 40.5 | *CCT8* | Chaperonin containing TCP1, subunit 8 (theta) (59.7kD, pI 5.6) | 58 |
|  |  |  | 9.2 | *PNP* | Purin Nucleosidee Phosphorylase (33kD, pI 7.2) | 2 |
|  |  |  | 5.5 | *FBXO7* | F-box protein 7 (58kD, pI 6.8) | 3 |
| 297 | 45 | 30 / 5.5 | 32.9 | *PSMA3* | Isoform 1 of Proteasome subunit alpha type-3 (28.4kD, pI 5.3) | 23 |
|  |  |  | 29.9 | *RANBP1* | Ran-specific GTPase-activating protein (23.3kD, pI 5.3) | 12 |
|  |  |  | 6.2 | *CCT8* | Chaperonin containing TCP1, subunit 8 (theta) | 3 |
|  |  |  |  |  | (59.7kD, pI 5.6) carry over |  |
| 24 | 46 | 45 / 5.8 | 31.7 | *ACTB* | Actin, cytoplasmic 2 (45kD, pI 6.2) | 86 |
|  |  |  | 14.6 | *actin* | Actin (42kD, pI 5.4) NOT UNIQUE PEPTIDE : | 23 |
|  |  |  |  |  | ACTA2 or ACTG2 or ACTC1 or ACTA1 |  |
|  |  |  | 12.8 | *ACTBL2* | Actin, beta-like 2 , DKFZp686D0972 hypothetical protein | 19 |
|  |  |  |  |  | LOC345651 (42kD, pI 5.6) |  |
|  |  |  | 6.9 | *POTEI* | POTE ankyrin domain family, member K, pseudogene, | 17 |
|  |  |  |  |  | (42kD, pI 6.3) |  |
|  |  |  | 2.2 | *LOC653781* | Similar to Prostate, ovary, testis expressed protein on | 4 |
|  |  |  |  |  | chromosome 2 (117kD, pI 6.0) |  |
| 159 | 47 | 22 / 6.5 | 37.4 | *PRDX2* | Peroxiredoxin-2 (21.8kD, pI 6.0*)* | 64 |
|  |  |  | 25.1 | *CCT8* | Chaperonin containing TCP1, subunit 8 (theta) (59.7kD, pI 5.6) | 17 |
|  |  |  | 3.8 | *POTE2* | POTE ankyrin domain family, member K, pseudogene, | 8 |
|  |  |  |  |  | (42kD, pI 6.3) |  |
|  |  |  | 10.1 | *ACTB* | Actin, cytoplasmic 2 (45kD*,* pI 6.2) | 8 |
| 227 | 48 | 55 / 5.8 | 56.7 | *USP14* | Ubiquitin carboxyl-terminal hydrolase 14 (56kD, pI 5.3) | 86 |
|  |  |  | 8.6 | *PRDX2* | Peroxiredoxin-2 (21.8kD, pI 6.0) | 3 |
|  |  |  | 5.6 | *EIF5* | Eukaryotic translation initiation factor 5 (49.2kD, pI 5.6) | 6 |

Spot rank: Rank of spot as assigned by Same Spots Software depending on fold change (normalized volume) comparing the highest to lowest sample

Pick #: Sequence in which spots were excised from gel depending on spot intensity (from lowest to highest)

Spot Location: Approximate location of spot by protein mass (marker on gel in kilo dalton, kD) and isoelectric point (pI) as estimated on focussing strip used

Coverage: Percentage of aminoacid sequence that is covered by unique peptides to identify protein

Gene: HGNC Symbol for coding human gene

Protein: HGNC Symbol for protein identified (including theoretical protein mass and pI)

SC: Spectral count
