# Supplementary material for: Assessment of the Red Cell Proteome of Young Patients with Unexplained Hemolytic Anemia by Two-Dimensional Differential In-Gel Electrophoresis (DIGE)
Source: PLoS One. 2012 Apr 3;7(4):e34237. doi: 10.1371/journal.pone.0034237 (PMC3317954; doi:10.1371/journal.pone.0034237)
Supplement: Table S15 — Complete list of all spots picked in HA24, fold change is comparing patient to all other samples run in the experiment. (DOCX) [file pone.0034237.s019.docx]

Table S15: Complete list of all spots picked in HA24, fold change is comparing patient to all other samples run in the experiment

| Spot rank | Pick # | Fold change |  | Patient | Mother | Father | Sister | Control | Standard |
| --- | --- | --- | --- | --- | --- | --- | --- | --- | --- |
| 7 | 1 |  | I | 2.672 | 0.635 | 1.043 | 0.506 | 0.469 | 0.410 |
|  |  |  | II | 2.771 | 0.699 | 1.055 | 0.586 | 0.430 | 0.392 |
|  |  |  | med | 2.722 | 0.667 | 1.049 | 0.546 | 0.450 | 0.401 |
|  | **P to all 4.37** | |  |  | **4.080** | **2.594** | **4.984** | **6.055** | **6.787** |
| 22 | 2 |  | I | 0.953 | 1.441 | 2.286 | 2.664 | 1.439 | 0.714 |
|  |  |  | II | 0.948 | 1.871 | 2.073 | 2.204 | 1.253 | 0.730 |
|  |  |  | med | 0.951 | 1.656 | 2.180 | 2.434 | 1.346 | 0.722 |
|  | *P to all 1.75* | |  |  | *1.74* | *2.29* | *2.56* | *1.42* | *0.76* |
| 14 | 3 |  | I | 3.559 | 0.680 | 2.097 | 0.878 | 0.997 | 1.077 |
|  |  |  | II | 2.841 | 0.796 | 1.954 | 0.699 | 1.172 | 0.980 |
|  |  |  | med | 3.200 | 0.738 | 2.026 | 0.789 | 1.085 | 1.029 |
|  | **P to all 2.82** | |  |  | **4.336** | **1.580** | **4.058** | **2.951** | **3.111** |
| 4 | 4 |  | I | 3.170 | 0.680 | 1.136 | 0.624 | 0.578 | 0.372 |
|  |  |  | II | 2.968 | 0.745 | 1.136 | 0.544 | 0.501 | 0.402 |
|  |  |  | med | 3.069 | 0.713 | 1.136 | 0.584 | 0.540 | 0.387 |
|  | **P to all 4.57** | |  |  | **4.307** | **2.702** | **5.255** | **5.689** | **7.930** |
| 41 | 5 |  | I | 0.377 | 0.727 | 0.993 | 1.122 | 0.694 | 0.793 |
|  |  |  | II | 0.383 | 0.840 | 0.967 | 0.999 | 0.783 | 0.808 |
|  |  |  | med | 0.380 | 0.784 | 0.980 | 1.061 | 0.739 | 0.801 |
|  | *P to all 1.78* | |  |  | *2.06* | *2.58* | *2.79* | *1.94* | *2.11* |
| 55 | 6 |  | I | 2.410 | 0.839 | 1.763 | 1.051 | 1.138 | 1.258 |
|  |  |  | II | 2.145 | 0.973 | 1.787 | 0.909 | 1.313 | 1.183 |
|  |  |  | med | 2.278 | 0.906 | 1.775 | 0.980 | 1.226 | 1.221 |
|  | **P to all 1.86** | |  |  | **2.514** | **1.283** | **2.324** | **1.858** | **1.866** |
| 52 | 7 |  | I | 1.134 | 0.616 | 0.702 | 1.264 | 0.468 | 0.991 |
|  |  |  | II | 1.163 | 0.571 | 0.731 | 1.241 | 0.514 | 0.975 |
|  |  |  | med | 1.149 | 0.594 | 0.717 | 1.253 | 0.491 | 0.983 |
|  | P to all 1.42 | |  |  | 1.935 | 1.603 | 0.917 | 2.339 | 1.168 |
| 60 | 8 |  | I | 1.301 | 1.115 | 0.872 | 1.569 | 0.934 | 0.632 |
|  |  |  | II | 1.247 | 1.130 | 0.941 | 1.567 | 0.947 | 0.651 |
|  |  |  | med | 1.274 | 1.123 | 0.907 | 1.568 | 0.941 | 0.642 |
|  | P to all 1.23 | |  |  | 1.135 | 1.405 | 0.813 | 1.355 | 1.986 |
| 1 | 9 |  | I | 0.127 | 1.105 | 2.743 | 0.222 | 0.995 | 2.401 |
|  |  |  | II | 0.099 | 1.533 | 1.539 | 0.156 | 1.543 | 1.358 |
|  |  |  | med | 0.113 | 1.319 | 2.141 | 0.189 | 1.269 | 1.880 |
|  | *P to all 12.03* | |  |  | *11.67* | *18.95* | *1.67* | *11.23* | *16.63* |
| 50 | 10 |  | I | 1.349 | 0.921 | 0.640 | 0.515 | 0.615 | 0.496 |
|  |  |  | II | 1.201 | 0.927 | 0.617 | 0.525 | 0.573 | 0.480 |
|  |  |  | med | 1.275 | 0.924 | 0.629 | 0.520 | 0.594 | 0.488 |
|  | **P to all 2.02** | |  |  | **1.380** | **2.029** | **2.452** | **2.146** | **2.613** |
| 76 | 11 |  | I | 0.624 | 1.520 | 1.007 | 0.825 | 0.965 | 0.738 |
|  |  |  | II | 0.859 | 1.372 | 0.919 | 0.753 | 0.985 | 0.578 |
|  |  |  |  | 0.742 | 1.446 | 0.963 | 0.789 | 0.975 | 0.658 |
|  | P to all 1.3 | | med |  | 1.95 | 1.30 | 1.06 | 1.31 | 0.89 |
| 132 | 12 |  | I | 1.736 | 1.315 | 0.993 | 0.856 | 1.018 | 1.033 |
|  |  |  | II | 1.568 | 1.299 | 0.978 | 0.947 | 1.016 | 1.063 |
|  |  |  | med | 1.652 | 1.307 | 0.986 | 0.902 | 1.017 | 1.048 |
|  | **P to all 1.57** | |  |  | **1.264** | **1.676** | **1.833** | **1.624** | **1.576** |
| 13 | 13 |  | I | 2.756 | 0.632 | 1.339 | 0.757 | 0.688 | 0.546 |
|  |  |  | II | 2.577 | 0.631 | 1.334 | 0.823 | 0.648 | 0.583 |
|  |  |  | med | 2.667 | 0.632 | 1.337 | 0.790 | 0.668 | 0.565 |
|  | **P to all 3.34** | |  |  | **4.222** | **1.995** | **3.375** | **3.992** | **4.724** |
| 20 | 14 |  | I | 1.237 | 0.348 | 0.742 | 0.882 | 0.780 | 1.177 |
|  |  |  | II | 1.248 | 0.378 | 0.772 | 0.871 | 0.747 | 1.249 |
|  |  |  | med | 1.243 | 0.363 | 0.757 | 0.877 | 0.764 | 1.213 |
|  | **P to all 1.56** | |  |  | **3.423** | **1.641** | **1.418** | **1.627** | **1.024** |
| 62 | 15 |  | I | 1.860 | 1.248 | 0.754 | 0.738 | 0.926 | 1.045 |
|  |  |  | II | 1.563 | 1.297 | 0.776 | 0.637 | 1.130 | 1.021 |
|  |  |  | med | 1.712 | 1.273 | 0.765 | 0.688 | 1.028 | 1.033 |
|  | **P to all 1.79** | |  |  | **1.345** | **2.237** | **2.489** | **1.665** | **1.657** |
| 94 | 16 |  | I | 0.565 | 1.158 | 1.005 | 1.100 | 1.064 | 0.933 |
|  |  |  | II | 0.625 | 1.020 | 1.031 | 1.296 | 0.921 | 1.065 |
|  |  |  | med | 0.595 | 1.089 | 1.018 | 1.198 | 0.993 | 0.999 |
|  | **P to all 1.78** | |  |  | **1.83** | **1.71** | **2.01** | **1.67** | **1.68** |
| 88 | 17 |  | I | 0.519 | 0.775 | 1.089 | 1.087 | 0.825 | 0.910 |
|  |  |  | II | 0.562 | 0.766 | 1.024 | 1.131 | 0.758 | 0.937 |
|  |  |  | med | 0.541 | 0.771 | 1.057 | 1.109 | 0.792 | 0.924 |
|  | *P to all 1.72* | |  |  | *1.43* | *1.95* | *2.05* | *1.46* | *1.71* |
| 64 | 18 |  | I | 0.640 | 0.904 | 1.160 | 1.541 | 0.868 | 1.534 |
|  |  |  | II | 0.606 | 0.976 | 1.062 | 1.337 | 0.895 | 1.465 |
|  |  |  | med | 0.623 | 0.940 | 1.111 | 1.439 | 0.882 | 1.500 |
|  | *P to all 1.88* | |  |  | *1.51* | *1.78* | *2.31* | *1.41* | *2.41* |
| 75 | 19 |  | I | 1.007 | 1.165 | 0.538 | 0.635 | 0.988 | 1.277 |
|  |  |  | II | 0.957 | 1.182 | 0.620 | 0.605 | 1.054 | 1.306 |
|  |  |  |  | 0.982 | 1.174 | 0.579 | 0.620 | 1.021 | 1.292 |
|  | P to all 0.95 | | med |  | 1.20 | 0.59 | 0.63 | 1.04 | 1.32 |
| 36 | 20 |  | I | 1.552 | 1.080 | 0.525 | 0.543 | 0.829 | 0.572 |
|  |  |  | II | 1.694 | 0.943 | 0.581 | 0.609 | 0.790 | 0.658 |
|  |  |  | med | 1.623 | 1.012 | 0.553 | 0.576 | 0.810 | 0.615 |
|  | **P to all 2.28** | |  |  | **1.605** | **2.935** | **2.818** | **2.005** | **2.639** |
| 38 | 21 |  | I | 0.763 | 0.581 | 1.717 | 0.538 | 1.410 | 0.899 |
|  |  |  | II | 0.660 | 0.555 | 1.460 | 0.563 | 1.498 | 0.787 |
|  |  |  | med | 0.712 | 0.568 | 1.589 | 0.551 | 1.454 | 0.843 |
|  | P to all 1.16 | |  |  | 1.253 | 0.448 | 1.292 | 0.489 | 0.844 |
| 201 | 22 |  | I | 0.598 | 0.794 | 0.991 | 0.993 | 0.724 | 0.936 |
|  |  |  | II | 0.608 | 0.802 | 1.006 | 0.942 | 0.801 | 0.899 |
|  |  |  | med | 0.603 | 0.798 | 0.999 | 0.968 | 0.763 | 0.918 |
|  | P to all 1.47 | |  |  | 1.32 | 1.66 | 1.60 | 1.26 | 1.52 |
| 167 | 23 |  | I | 1.265 | 1.095 | 0.767 | 0.721 | 0.787 | 0.789 |
|  |  |  | II | 1.250 | 1.009 | 0.747 | 0.725 | 0.769 | 0.784 |
|  |  |  | med | 1.258 | 1.052 | 0.757 | 0.723 | 0.778 | 0.787 |
|  | **P to all 1.53** | |  |  | **1.195** | **1.661** | **1.739** | **1.616** | **1.599** |
| 81 | 24 | 2.1 | I | 0.632 | 1.161 | 1.361 | 0.995 | 1.095 | 0.849 |
|  |  |  | II | 0.591 | 1.241 | 1.247 | 0.922 | 1.249 | 0.786 |
|  |  |  | med | 0.612 | 1.201 | 1.304 | 0.959 | 1.172 | 0.818 |
|  | *P all 1.78* | |  |  | *1.96* | *2.13* | *1.57* | *1.92* | *1.34* |
| 138 | 25 |  | I | 0.889 | 0.995 | 1.625 | 1.112 | 1.047 | 1.223 |
|  |  |  | II | 0.843 | 1.030 | 1.509 | 1.005 | 1.181 | 1.236 |
|  |  |  | med | 0.866 | 1.013 | 1.567 | 1.059 | 1.114 | 1.230 |
|  | P to all 1.38 | |  |  | 1.17 | 1.81 | 1.22 | 1.29 | 1.42 |
| 285 | 26 |  | I | 0.684 | 0.805 | 0.961 | 1.015 | 0.948 | 1.053 |
|  |  |  | II | 0.720 | 0.780 | 0.926 | 1.105 | 0.938 | 1.075 |
|  |  |  | med | 0.702 | 0.793 | 0.944 | 1.060 | 0.943 | 1.064 |
|  | P to all 1.37 | |  |  | 1.13 | 1.34 | 1.51 | 1.34 | 1.52 |
| 126 | 27 |  | I | 0.726 | 0.715 | 0.900 | 0.966 | 0.976 | 1.291 |
|  |  |  | II | 0.749 | 0.704 | 0.970 | 1.003 | 0.954 | 1.354 |
|  |  |  | med | 0.738 | 0.710 | 0.935 | 0.985 | 0.965 | 1.323 |
|  | P to all 1.33 | |  |  | 0.96 | 1.27 | 1.33 | 1.31 | 1.79 |
| 49 | 28 |  | I | 1.538 | 1.094 | 0.661 | 0.626 | 0.849 | 0.567 |
|  |  |  | II | 1.545 | 1.024 | 0.722 | 0.665 | 0.742 | 0.611 |
|  |  |  | med | 1.542 | 1.059 | 0.692 | 0.646 | 0.796 | 0.589 |
|  | **P to all 2.13** | |  |  | **1.456** | **2.229** | **2.388** | **1.938** | **2.617** |
| 56 | 29 |  | I | 1.342 | 0.968 | 0.628 | 0.533 | 0.792 | 0.860 |
|  |  |  | II | 1.409 | 0.968 | 0.621 | 0.562 | 0.965 | 0.979 |
|  |  |  | med | 1.376 | 0.968 | 0.625 | 0.548 | 0.879 | 0.920 |
|  | **P to all 1.75** | |  |  | **1.421** | **2.203** | **2.512** | **1.566** | **1.496** |
| 173 | 30 |  | I | 0.610 | 0.962 | 1.049 | 0.950 | 1.042 | 0.939 |
|  |  |  | II | 0.628 | 0.957 | 1.083 | 0.934 | 1.022 | 1.009 |
|  |  |  | med | 0.619 | 0.960 | 1.066 | 0.942 | 1.032 | 0.974 |
|  | *P to all 1.61* | |  |  | *1.55* | *1.72* | *1.52* | *1.67* | *1.57* |
| 111 | 31 |  | I | 0.577 | 0.897 | 1.016 | 1.080 | 1.043 | 1.041 |
|  |  |  | II | 0.612 | 0.836 | 1.018 | 1.209 | 0.904 | 1.113 |
|  |  |  | med | 0.595 | 0.867 | 1.017 | 1.145 | 0.974 | 1.077 |
|  | *P to all 1.71* | |  |  | *1.46* | *1.71* | *1.93* | *1.64* | *1.81* |
| 179 | 32 |  | I | 0.685 | 0.940 | 1.057 | 1.078 | 0.978 | 1.073 |
|  |  |  | II | 0.572 | 1.091 | 0.893 | 0.891 | 1.171 | 0.913 |
|  |  |  | med | 0.629 | 1.016 | 0.975 | 0.985 | 1.075 | 0.993 |
|  | *P to all 1.6* | |  |  | *1.62* | *1.55* | *1.57* | *1.71* | *1.58* |
| 210 | 33 |  | I | 0.561 | 0.889 | 0.931 | 0.887 | 0.980 | 0.859 |
|  |  |  | II | 0.569 | 0.858 | 0.910 | 0.922 | 0.885 | 0.882 |
|  |  |  | med | 0.565 | 0.874 | 0.921 | 0.905 | 0.933 | 0.871 |
|  | *P to all 1.59* | |  |  | *1.55* | *1.63* | *1.60* | *1.65* | *1.54* |
| 140 | 34 | 1.8 | I | 0.802 | 1.102 | 0.726 | 1.007 | 0.908 | 1.334 |
|  |  |  | II | 0.678 | 1.194 | 0.710 | 0.862 | 1.046 | 1.268 |
|  |  |  | med | 0.740 | 1.148 | 0.718 | 0.935 | 0.977 | 1.301 |
|  | P to all 1.37 | |  |  | 1.55 | 0.97 | 1.26 | 1.32 | 1.76 |
| 47 | 35 |  | I | 1.503 | 1.044 | 0.639 | 0.551 | 0.895 | 0.807 |
|  |  |  | II | 1.455 | 0.985 | 0.652 | 0.539 | 0.946 | 0.850 |
|  |  |  | med | 1.479 | 1.015 | 0.646 | 0.545 | 0.921 | 0.829 |
|  | **P to all 2.04** | |  |  | **1.458** | **2.291** | **2.714** | **1.607** | **1.785** |
| 80 | 36 |  | I | 0.813 | 1.143 | 1.255 | 1.058 | 1.077 | 0.608 |
|  |  |  | II | 0.829 | 1.043 | 1.313 | 1.105 | 1.189 | 0.600 |
|  |  |  | med | 0.821 | 1.093 | 1.284 | 1.082 | 1.133 | 0.604 |
|  | P to all 1.27 | |  |  | 1.33 | 1.56 | 1.32 | 1.38 | 0.74 |
| 122 | 37 |  | I | 0.611 | 0.933 | 1.159 | 1.045 | 0.814 | 0.753 |
|  |  |  | II | 0.621 | 0.847 | 1.154 | 1.176 | 0.755 | 0.756 |
|  |  |  | med | 0.616 | 0.890 | 1.157 | 1.111 | 0.785 | 0.755 |
|  | *P to all 1.52* | |  |  | *1.44* | *1.88* | *1.80* | *1.27* | *1.22* |
| 145 | 38 |  | I | 0.741 | 0.682 | 0.967 | 1.053 | 0.894 | 1.209 |
|  |  |  | II | 0.712 | 0.661 | 0.980 | 1.042 | 0.941 | 1.206 |
|  |  |  | med | 0.727 | 0.672 | 0.974 | 1.048 | 0.918 | 1.208 |
|  | P to all 1.33 | |  |  | 0.92 | 1.34 | 1.44 | 1.26 | 1.66 |
| 305 | 39 |  | I | 0.810 | 1.057 | 1.212 | 1.150 | 1.105 | 1.134 |
|  |  |  | II | 0.751 | 1.094 | 1.105 | 1.110 | 1.205 | 1.100 |
|  |  |  | med | 0.781 | 1.076 | 1.159 | 1.130 | 1.155 | 1.117 |
|  | P to all 1.44 | |  |  | 1.38 | 1.48 | 1.45 | 1.48 | 1.43 |
| 43 | 40 |  | I | 1.704 | 1.097 | 0.640 | 0.689 | 0.767 | 0.698 |
|  |  |  | II | 1.655 | 1.076 | 0.571 | 0.720 | 0.835 | 0.648 |
|  |  |  | med | 1.680 | 1.087 | 0.606 | 0.705 | 0.801 | 0.673 |
|  | **P to all 2.17** | |  |  | **1.546** | **2.774** | **2.384** | **2.097** | **2.496** |
| 165 | 41 |  | I | 0.887 | 0.785 | 1.227 | 1.516 | 1.030 | 1.134 |
|  |  |  | II | 0.796 | 0.834 | 1.284 | 1.288 | 1.094 | 1.152 |
|  |  |  | med | 0.842 | 0.810 | 1.256 | 1.402 | 1.062 | 1.143 |
|  | P to all 1.35 | |  |  | 0.96 | 1.49 | 1.67 | 1.26 | 1.36 |
| 284 | 42 |  | I | 0.652 | 0.843 | 0.982 | 1.008 | 0.886 | 1.002 |
|  |  |  | II | 0.681 | 0.803 | 0.962 | 0.965 | 0.883 | 1.026 |
|  |  |  | med | 0.667 | 0.823 | 0.972 | 0.987 | 0.885 | 1.014 |
|  | P to all 1.4 | |  |  | 1.23 | 1.46 | 1.48 | 1.33 | 1.52 |
| 23 | 44 |  | I | 1.486 | 0.986 | 0.592 | 0.444 | 1.008 | 0.867 |
|  |  |  | II | 1.506 | 0.956 | 0.581 | 0.460 | 1.028 | 0.966 |
|  |  |  | med | 1.496 | 0.971 | 0.587 | 0.452 | 1.018 | 0.917 |
|  | **P to all 1.9** | |  |  | **1.541** | **2.551** | **3.310** | **1.470** | **1.632** |
| 97 | 44 |  | I | 0.644 | 0.657 | 0.986 | 1.075 | 0.946 | 1.296 |
|  |  |  | II | 0.655 | 0.682 | 0.971 | 0.982 | 1.013 | 1.313 |
|  |  |  | med | 0.650 | 0.670 | 0.979 | 1.029 | 0.980 | 1.305 |
|  | *P to all 1.53* | |  |  | *1.03* | *1.51* | *1.58* | *1.51* | *2.01* |
| 297 | 45 |  | I | 0.699 | 0.794 | 0.927 | 0.960 | 0.890 | 1.065 |
|  |  |  | II | 0.705 | 0.807 | 0.906 | 0.974 | 0.881 | 1.046 |
|  |  |  | med | 0.702 | 0.801 | 0.917 | 0.967 | 0.886 | 1.056 |
|  | P to all 1.32 | |  |  | 1.14 | 1.31 | 1.38 | 1.26 | 1.50 |
| 24 | 46 |  | I | 1.823 | 0.570 | 0.710 | 1.003 | 0.582 | 0.764 |
|  |  |  | II | 1.902 | 0.561 | 0.670 | 1.038 | 0.588 | 1.002 |
|  |  |  | med | 1.863 | 0.566 | 0.690 | 1.021 | 0.585 | 0.969 |
|  | **P to all 2.49** | |  |  | **3.294** | **2.699** | **1.825** | **3.184** | **1.922** |
| 159 | 47 |  | I | 1.207 | 1.238 | 0.723 | 0.772 | 0.845 | 0.808 |
|  |  |  | II | 1.338 | 1.118 | 0.723 | 0.756 | 0.761 | 0.861 |
|  |  |  | med | 1.273 | 1.178 | 0.723 | 0.764 | 0.803 | 0.969 |
|  | P to all 1.48 | |  |  | 1.080 | 1.760 | 1.666 | 1.585 | 1.313 |
| 227 | 48 |  | I | 0.715 | 0.958 | 1.116 | 1.125 | 0.928 | 1.033 |
|  |  |  | II | 0.700 | 0.948 | 1.106 | 1.146 | 0.919 | 1.014 |
|  |  |  | med | 0.708 | 0.953 | 1.111 | 1.136 | 0.924 | 1.024 |
|  | P to all 1.45 | |  |  | 1.35 | 1.57 | 1.60 | 1.31 | 1.45 |

Spot rank: Rank of spot as assigned by Same Spots Software depending on fold change (normalized volume) comparing the highest to lowest sample

Pick #: Sequence in which spots were excised from gel depending on spot intensity (from lowest to highest)

Roman Numerals: Normalized volume measured in replicate runs one (I) and two (II)

Med: Average of normalized volume measured in replicate runs one (I) and two (II)

Fold Change P to all: Expression level (normalized volume) of patient sample compared to the average of all other samples combined;

Last row of columns also shows fold change comparing patient to sample indicated in header of column (Mother, Father, Control, Sister, Standard)

Fromatting : **Bold**: Patient upregulated *Italic*: Patient downregulated

No special formatting: Fold change “P to all” does not exceed 1.5 fold
